# Tailoring the Oxidative Chemistry of Hydroxycinnamyl Alcohols: New and Green Access Routes to Lignans and Lignin Functional Derivatives

**DOI:** 10.1021/acs.jafc.6c09238

**Published:** 2026-09-15

**Authors:** Carmen Ruoppolo, Maria Laura Alfieri, Alessandra Napolitano, Lucia Panzella

**Affiliations:** Department of Chemical Sciences, 9307 University of Naples Federico II, Via Cintia 4, I-80126 Naples, Italy

**Keywords:** Hydroxycinnamyl alcohols, Oxidative chemistry, Solid-state, Lignans

## Abstract

The oxidative reactivity of coniferyl alcohol (CA), sinapyl alcohol (SA), and *p*-coumaryl alcohol (PCA), the main lignin precursors, was systematically investigated under enzymatic and non-enzymatic conditions in both solution and solid-state. For CA, aqueous oxidation promoted the formation of β-O-4 and β-5 dimers, whereas organic solvents favored selective β-5 dimer formation. Remarkably, solvent-free mechanochemical oxidation with silver oxide provided access to the trimeric guaiacylglycerol-α,β-bisconiferyl derivative. Comparative studies showed that SA readily formed β-β dimer and β-O-4 dimers, whereas PCA remained largely unreactive. Co-oxidation of CA and SA favored a cross-coupling reaction yielding a β-5 heterodimer. Finally, oxidation of CA or SA in the presence of nucleophiles (*e.g.*, lysine, glutathione) afforded new conjugation products, which could be exploited as precursors of water-soluble lignin polymers. Overall, these results provide insights into the oxidative chemistry of lignin monomers and establish a versatile platform for the design of lignin-inspired building blocks and functional materials.

## Graphical abstract

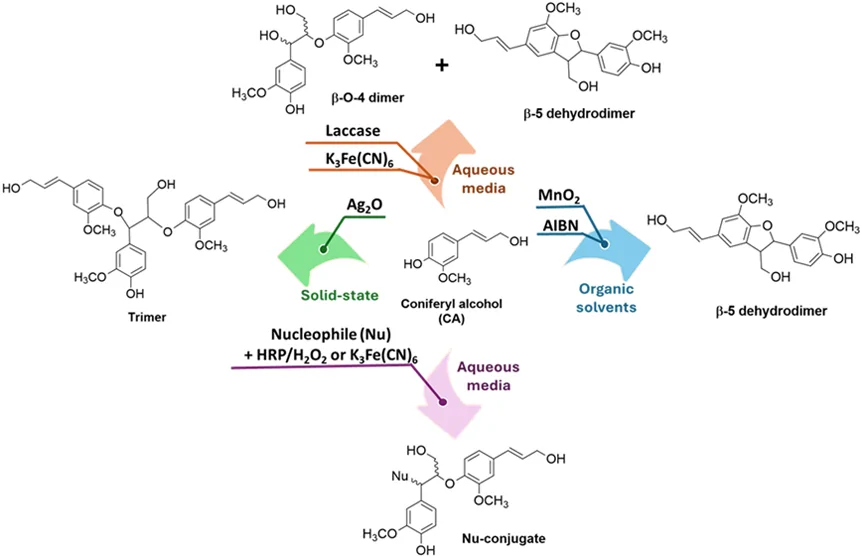

## Introduction

Natural phenolic compounds represent a versatile class of biomolecules that have attracted increasing attention in the last decades owing to their easy availability, structural diversity, and wide range of functional properties. Among them, lignin occupies a prominent position, since it composes up to a third of the plant cellular wall and is the most abundant biopolymer on earth after cellulose.^1–5^ Beyond its essential role in plant cell walls, its functional properties including antioxidant, UV-shielding, anticancer and antimicrobial activities, as well as its potential as a renewable source of aromatic building blocks, make lignin and lignin-inspired materials highly appealing for applications in the food, biomedical, cosmetic, and energy sectors.^6–16^

Despite its abundance and unique functionalities, the direct exploitation of natural lignin remains challenging. In fact, its structural complexity, arising not only from the variability of plant species, age, and growing conditions, but also from the specific extraction procedures employed which often induce significant structural modifications, limits its processability and complicates its integration into advanced materials.^17–21^ Critical steps toward the exploitation of the full potential of lignin are therefore not only the elucidation of its molecular structure, with particular emphasis on the identification and quantification of the functional groups and type of bonds present, but also the possibility to get access to compounds with a higher degree of structural homogeneity, exhibiting more defined physico-chemical properties.^22,23^

From a biosynthetic viewpoint, lignin originates from the oxidative polymerization of three major hydroxycinnamyl alcohols, that is coniferyl alcohol (CA), sinapyl alcohol (SA), and *p*-coumaryl alcohol (PCA) (Figure 1), which differ in the methoxylation degree of the aromatic ring. These alcohols are connected through numerous C–O–C and C–C linkages in the final polymer, such as β-O-4, α-O-4, 4-O-5, β-5, β-1 and β-β bonds.^24–33^ Understanding the oxidative coupling pathways of these monolignols is therefore central to unravel the complex structure and biosynthetic process of lignin. In addition, the study of the oxidative chemistry of these compounds can provide access to novel nature-inspired compounds, which may display manifold advantages over their natural counterparts.^34–38^

**Figure 1:**
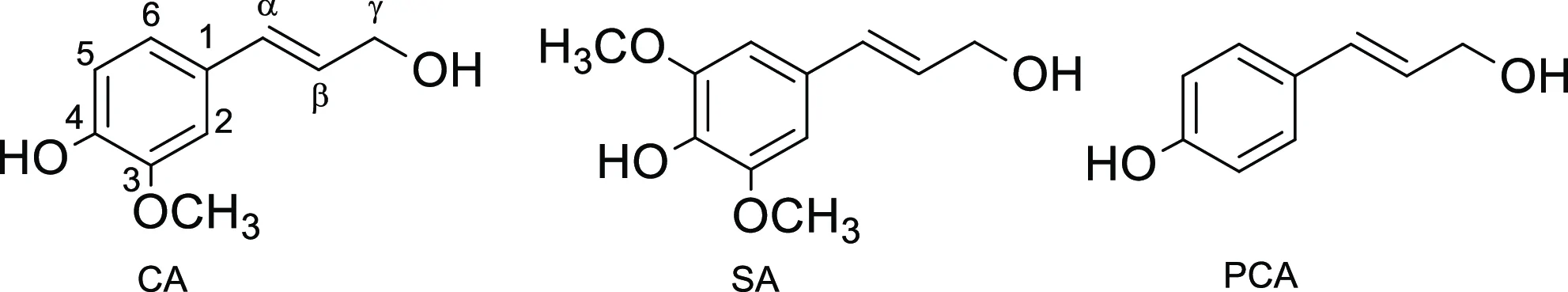
Structures of the main monomeric precursors of lignin: coniferyl alcohol (CA), sinapyl alcohol (SA), and *p*-coumaryl alcohol (PCA).

Although the oxidative chemistry of hydroxycinnamyl alcohols has been extensively investigated,^24,39–44^ a comprehensive comparison of the three principal monolignols under a common set of oxidation conditions has received limited attention. Herein, we systematically investigated the oxidative chemistry of CA, with the dual aim of gaining a deeper insight into the polymerization modes of this compound in lignin biosynthesis and, at the same time, developing new pathways to access to novel bioinspired compounds. Oxidation was carried out using both enzymatic and non-enzymatic systems in different solvents and under solid-state conditions. The exploration was then extended to the other two main lignin precursors, SA and PCA under the same oxidizing conditions adopted for CA, enabling a direct comparison of their reactivity patterns and opening opportunities for the selective synthesis of lignin-derived architectures and functional conjugates.

## Experimental Section

### General Protocol for Hydroxycinnamyl Alcohol Oxidation in Aqueous Solution

To a solution of CA, SA or PCA (0.027 mmol, 4.0–5.7 mg, pre-dissolved in 1 mL of methanol) in 50 mM sodium phosphate buffer (pH 6.8, final concentration 1 mM) horseradish peroxidase (HRP) (1 U/mL) and 0.2 molar eqs of hydrogen peroxide (H_2_O_2_) were added. The reaction mixture was taken under stirring at room temperature and periodically analyzed by LC-UV-MS. Full experimental details of all oxidation conditions are provided in the Supporting Information.

For preparative purposes the reaction of CA was run starting from 150 mg of hydroxycinnamyl alcohol with 2 molar eqs of potassium ferricyanide (K_3_Fe­(CN)_6_) (549 mg) in 50 mM sodium phosphate buffer (pH 6.8) (843 mL). After 1 h the mixture was extracted with ethyl acetate (3 × 500 mL). The combined organic layers were dried over sodium sulfate and taken to dryness. The residue thus obtained was fractionated by preparative TLC (eluent chloroform/methanol/acetic acid 9.5:0.5:0.01 v/v/v) to afford two products with *R*_f_ values of 0.20 and 0.25, which were identified by LC-MS and NMR analysis as the guaiacylglycerol-β-coniferyl ethers (*erythro* and *threo*, **1** and **2**) (5 mg, 3% w/w yield) and the β-5 *trans*-dehydrodimer (**3**) (44 mg, 29% w/w yield), respectively.

### Guaiacylglycerol-β-Coniferyl Ethers (**1**, **2**)

ESI–/MS: [M-H]^‑^ *m*/*z* 375; ^1^H NMR (acetone-*d*_6_) δ (ppm) (1:1 *erythro:threo* mixture): 3.53 (1H, dd, *J* = 11.6, 5.6 Hz), 3.66-3.72 (2H, m), 3.80 (1H, dd, *J* = 11.6, 5.6 Hz), 3.84 (6H, s), 3.87 (3H, s), 3.92 (3H, s), 4.22 (4H, brdd, *J* = 5.6, 1.6 Hz), 4.31 (1H, m), 4.42 (1H, d, *J* = 3.6 Hz), 4.51 (1H, d, *J* = 4.8 Hz), 4.90 (2H, m), 6.29 (1H, dt, *J* = 8.4, 3.2 Hz), 6.33 (1H, dt, *J* = 8.4, 3.2 Hz), 6.52 (1H, dt, *J* = 8.8, 1.6 Hz), 6.56 (1H, dt, *J* = 8.8, 1.6 Hz), 6.78 (2H, m), 6.92 (5H, m), 7.08 (1H, d, 1.6 Hz), 7.11 (2H, d, 1.6 Hz), 7.13 (2H, d, 8.4 Hz), 7.42 (1H, s), 7.46 (1H, s).

### CA β-**5** *trans*-Dehydrodimer (**3**)

ESI–/MS: [M-H]^‑^ *m*/*z* 357; ^1^H NMR (methanol-*d*_4_) δ (ppm): 3.48 (1H, m), 3.81 (3H, s), 3.83 (2H, m), 3.86 (3H, s), 4.20 (2H, d, 6.0 Hz), 5.52 (1H, d, 6.4 Hz), 6.23 (1H, dt, 15.6, 6.0 Hz), 6.54 (1H, d, 15.6 Hz), 6.77 (1H, d, 8.0 Hz), 6.82 (1H, dd, 8.0, 2.0 Hz), 6.95 (3H, m); ^13^C NMR (methanol-*d*_4_) δ (ppm): 53.7 (CH), 54.9 (-OCH_3_), 55.3 (-OCH_3_), 62.4 (CH_2_), 63.4 (CH_2_), 87.9 (CH), 109.1 (CH), 110.6 (CH), 114.7 (CH), 115.1 (CH), 118.3 (CH), 126.1 (CH), 128.9 (C), 130.6 (CH), 131.1 (C), 133.1 (C), 144.0 (C), 146.1 (C), 147.7 (C), 147.8 (C).

In the case of SA, for preparative purposes the reaction was run starting from 100 mg of hydroxycinnamyl alcohol with 1 molar eq of K_3_Fe­(CN)_6_ (157 mg) in 50 mM sodium phosphate buffer (pH 6.8) (476 mL). After 1 h the mixture was extracted with ethyl acetate (3 × 500 mL). The combined organic layers were dried over sodium sulfate and taken to dryness. The residue thus obtained was fractionated by preparative TLC (eluent chloroform/methanol/acetic acid 9.5:0.5:0.01 v/v/v) to afford the β-β dimer syringaresinol (**6**) (*R*_f_ = 0.33, 17 mg, 17% w/w yield).

### Syringaresinol (**6**)

ESI–/MS: [M-H]^‑^ *m*/*z* 417; ^1^H NMR (acetone-*d*_6_): 3.12 (2H, m), 3.84 (12H, s), 3.87 (2H, m), 4.25 (2H, m), 4.69 (2H, d, *J* = 4.0), 6.70 (4H, s).

In separate experiments, 5 mg of each alcohol (CA, SA and PCA) dissolved in 1 mL of methanol, were added to 26 mL of 50 mM sodium phosphate buffer (pH 6.8), followed by 9 mg of K_3_Fe­(CN)_6_ (1 molar eq). The reaction mixture was taken under stirring at room temperature and periodically analyzed by LC-UV-MS. In other experiments the oxidation reaction was performed:

- in the presence of 3 molar eqs of K_3_Fe­(CN)_6_ (27 mg);
- in the presence of HRP (3 U/mL) and 0.6 molar eqs of H_2_O_2_.

For preparative purposes the reaction of CA and SA was run starting from 50 mg of each alcohol with 3 U/mL of HRP (3.8 mg) and 0.6 molar eqs of H_2_O_2_ (17 μL) in 50 mM sodium phosphate buffer (pH 6.8) (278 mL). After 1 h the mixture was extracted with ethyl acetate (3 × 300 mL). The combined organic layers were dried over sodium sulfate and taken to dryness. The residue thus obtained was fractionated by preparative TLC (eluent chloroform/methanol/acetic acid 9.5:0.5:0.01 v/v/v) to afford simulanol (**9**) (*R*_f_ = 0.2, 8 mg, 8% w/w yield) in pure form.

### Simulanol (**9**)

ESI–/MS: [M-H]^‑^ *m*/*z* 387; ^1^H NMR (acetone-*d*_6_): 3.55 (1H, m), 3.79 (6H, s), 3.87 (3H, s), 3.87 (2H, m), 4.19 (2H, td, *J* = 5.6, 1.6 Hz), 5.55 (1H, d, *J* = 6.8 Hz), 6.24 (1H, dt, *J* = 15.6, 5.6 Hz), 6.52 (1H, dt, 15.6, 1.6 Hz), 6.74 (2H, s), 6.94 (1H, s), 6.97 (1H, s), 7.23 (1H, s).

### General Protocol for the Hydroxycinnamyl Alcohol Oxidation in Organic Solvents

To a solution of CA, SA or PCA (0.027 mmol, 4.0-5.7 mg) in 27 mL of methanol (final concentration 1 mM) 2,2-diphenyl-1-picrylhydrazyl (DPPH) (1 molar eq, 9-13 mg) was added. The reaction mixture was kept under stirring at room temperature and periodically analyzed by LC-UV-MS. Full experimental details of all oxidation conditions are provided in the Supporting Information.

In the case of SA, for preparative purposes the reaction was run starting from 50 mg of hydroxycinnamyl alcohol with 0.8 molar eqs of iron chloride (FeCl_3_) (30.8 mg) in a 5:1 v/v dioxane/water mixture (3.3 mL). After 1 h, the reaction mixture was treated as described above and then fractionated by preparative TLC (eluent chloroform/methanol/acetic acid 9.5:0.5:0.01 v/v/v) to give the syringylglycerol-β-sinapyl ethers (**7** and **8**) (*R*_f_ = 0.25, 4 mg, 8% w/w yield).

### Syringylglycerol-β-Sinapyl Ethers (**7**, **8**)

ESI–/MS: [M-H]^‑^ *m*/*z* 435; ^1^H NMR (acetone-*d*_6_) δ (ppm) (2.5:1 *erythro:threo* mixture): 3.35 (1H, m, minor diastereoisomer), 3.45 (1H, m, major diastereoisomer), 3.66 (1H, m, minor diastereoisomer), 3.81 (6H, s, major diastereoisomer), 3.83 (6H, s, minor diastereoisomer), 3.85 (1H, m, major diastereoisomer), 3.90 (6H, s, major diastereoisomer), 3.92 (6H, s, minor diastereoisomer), 3.95 (1H, m, minor diastereoisomer) 4.18 (1H, m, major diastereoisomer), 4.24 (2H, m, both diastereoisomers), 4.34 (1H, m, both diastereoisomers), 4.99 (1H, m, both diastereoisomers), 6.39 (1H, dt, *J* = 16.0 Hz, 5.6 Hz, both diastereoisomers), 6.56 (1H, d, *J* = 16.0 Hz, both diastereoisomers), 6.72 (2H, s, major diastereoisomer), 6.77 (2H, s, minor diastereoisomer), 6.82 (2H, s, both diastereoisomers).

### Solid-State Oxidation of Hydroxycinnamyl Alcohols

5 mg of CA, SA or PCA were placed in a 5 mL grinding jar made of stainless steel with two 2 mm-diameter stainless steel grinding balls in the presence of K_3_Fe­(CN)_6_ (1-5 molar eq). The mixture was milled for 30 min in a mini-mill Pulverisette 23 (Fritsch) operating at a frequency of 50 oscillation/s at room temperature, after that the powder was dissolved in methanol or acetone and analyzed by LC-UV-MS. Full experimental details of all oxidation conditions are provided in the Supporting Information.

In the case of CA, for preparative purposes the reaction was run starting from 50 mg of alcohol with 2 molar eqs of silver­(I) oxide (Ag_2_O) (129 mg). After 30 min milling, the powder was recovered in acetone (10 mL), centrifuged (5000 rpm, 2 min, 4°C) to remove residual oxidant and dried under vacuum. The residue was then redissolved in acetone and fractionated by preparative TLC (eluent chloroform/ethyl acetate 6:4 v/v) to give guaiacylglycerol-a,ß-bisconiferyl ether (**5**) (*R*_f_ = 0.09, 6.0 mg, 12% w/w yield).

### Guaiacylglycerol-a,ß-Bisconiferyl Ether (**5**)

ESI–/MS: [M-H]^−^ m/z 537; ^1^H NMR (acetone-*d*_6_) δ (ppm): 3.82 (3H, s), 3.83 (3H, s), 3.87 (1H, m), 3.91 (3H, s), 3.94 (1H, dd, 11.2, 5.2 Hz), 4.19 (2H, td, 5.6, 5.6, 1.6 Hz), 4.22 (2H, td, 5.6, 1.6 Hz), 4.58 (1H, brq), 5.48 (1H, d, 5.6 Hz), 6.24 (1 H, dt, 16.0, 5.6 Hz), 6.29 (1 H, dt, 16.0, 5.6 Hz), 6.48 (1 H, dt, 16.0, 1.6 Hz), 6.53 (1 H, dt, 16.0, 1.6 Hz), 6.77-6.80 (3 H, m), 6.89 (1H, dd, 8.0, 2.4 Hz), 6.96 (1H, dd, 8.0, 2.0 Hz), 7.01 (1 H, d, 8.0 Hz), 7.06 (1 H, d, 2.4 Hz), 7.07 (1 H, d, 2.4 Hz), 7.21 (1 H, d, 2.0 Hz), 7.5 (1H, s); ^13^C NMR (acetone-*d*_6_) δ (ppm): 55.3 (-OCH_3_), 55.5 (-OCH_3_), 60.9 (CH_2_), 62.4 (CH_2_), 80.2 (CH), 84.5 (CH), 109.9 (CH), 110.1 (CH), 111.3 (CH), 114.4 (CH), 116.2 (CH), 118.2 (CH), 119.1 (CH), 119.3 (CH), 120.7 (CH), 128.4 (CH), 128.6 (CH), 129.0 (CH), 129.6 (C), 131.2 (C), 131.9 (C), 146.4 (C), 146.9 (C), 147.3 (C), 147.8 (C), 150.3 (C), 150.9 (C).

## Results and Discussion

### Preparation of the Hydroxycinnamyl Alcohols

Given the high costs of commercially available CA, SA, and PCA on a gram scale, the compounds were synthesized by adopting a simple literature protocol with slight modifications.^45^ The procedure involved the simultaneous protection and activation of the corresponding carboxylic acids, followed by reduction with sodium borohydride and subsequent deprotection with ammonium hydroxide (Figure 2). This strategy afforded the desired hydroxycinnamyl alcohols in good yields (55-70% w/w) and with a satisfactory degree of purity (>90%), as confirmed by NMR (Figure S1) and LC-UV-MS analysis (Figures S2 and S3), without the need for purification steps.

**Figure 2:**
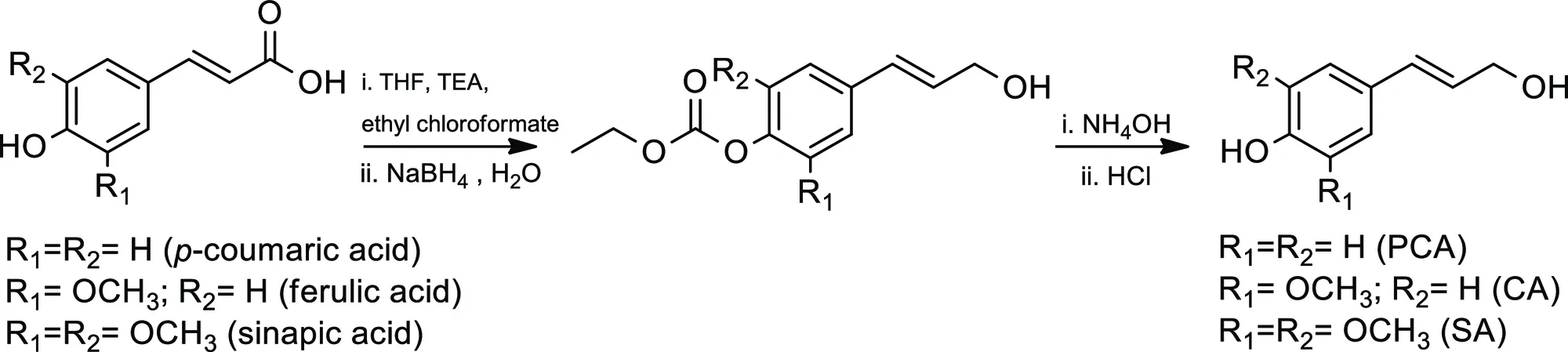
Synthesis of the hydroxycinnamyl alcohols coniferyl alcohol (CA), sinapyl alcohol (SA), and *p*-coumaryl alcohol (PCA).

### Investigation of the Oxidative Chemistry of CA in Aqueous Solution

The oxidation of CA was initially carried out under biomimetic conditions using the HRP/H_2_O_2_ system, which is well known to be involved in lignin biosynthesis.^46–49^ The most promising results, in terms of mass balance between substrate consumption and product formation, were obtained at 1 mM phenol concentration in 50 mM phosphate buffer at pH 6.8, with 1 U/mL HRP and 0.4 molar eqs of H_2_O_2_. LC-UV-MS analysis of the reaction mixture indicated, after 1 h, a 38% consumption of the starting phenol and the formation of three main products (indicated as **1-3** in Figure S4). Mass spectra of products **1** and **2** showed a pseudomolecular ion peak [M-H]^‑^ at *m*/*z* 375 (Figure S4), while product **3** exhibited a pseudomolecular ion peak [M-H]^‑^ at *m*/*z* 357 (Figure S4), both consistent with dimeric derivatives of CA. Whereas several CA dimeric structures with a molecular weight of 358 Da have been reported in literature,^39,50–53^ the molecular weight of 376 Da is uniquely associated with the β-O-4 dimers guaiacylglycerol-β-coniferyl ethers,^52^ that differ from simple dimeric products for the presence of an additional hydroxyl group. The presence of two stereogenic centers accounts for the occurrence of two diastereoisomers (*erythro* and *threo*, each as a pair of enantiomers), in agreement with the chromatographic profile (Figure S4) showing two isomeric products (**1** and **2**).

To enhance the yields of formation of products **1-3** for their subsequent purification and structural characterization, in another series of experiments the possibility of using other oxidizing systems was explored. LC-UV-MS analysis of the reaction mixture of CA (1 mM) with 2 molar eqs of K_3_Fe­(CN)_6_ showed after 1 h a higher phenol consumption (55%) and an increased formation of products **1-3** (Figure 3), compared with the reaction carried out using the HRP/H_2_O_2_ system (Figure S4).

**Figure 3:**
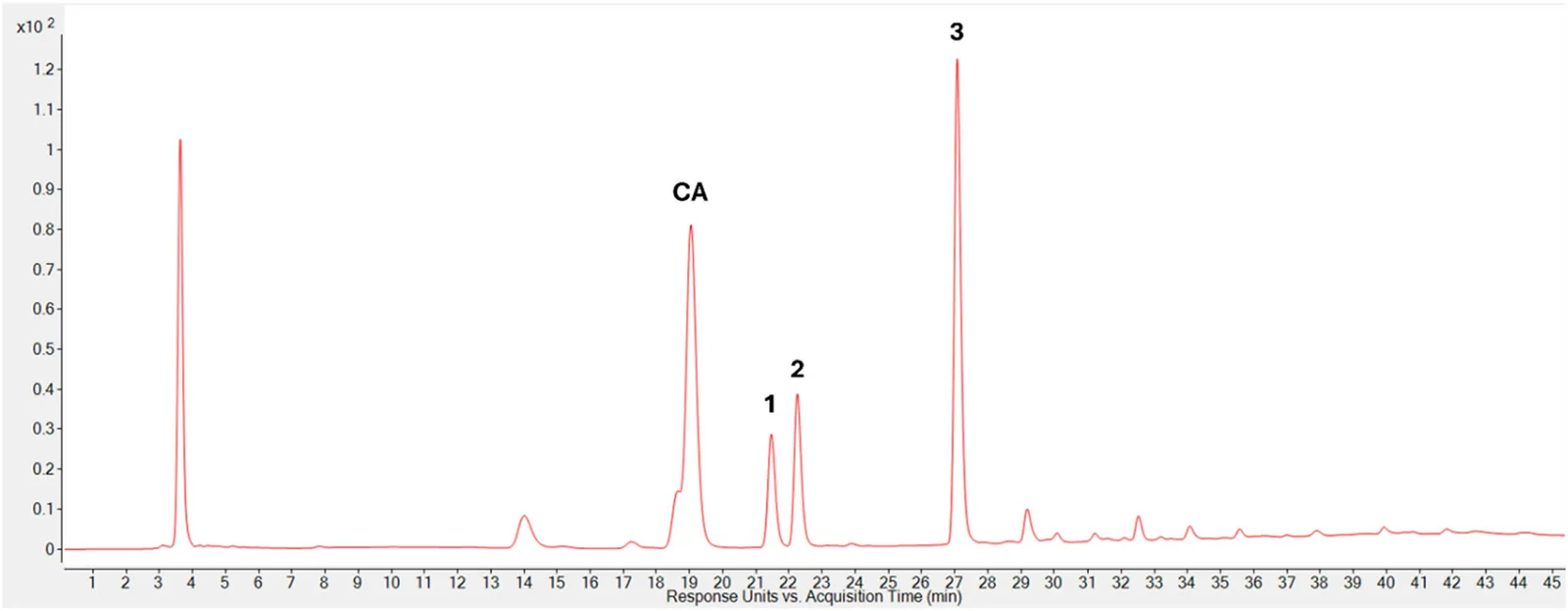
HPLC profile of the reaction mixture of coniferyl alcohol (CA) in the presence of 2 molar eqs of K_3_Fe­(CN)_6_ at pH 6.8 after 1 h. Analysis was performed under the following conditions: Eclipse Plus C18 column (150 × 4.6 mm^2^, 5 μm). Eluent: 0.1% formic acid (solvent A)/methanol (solvent B): 30% B (0–10 min), from 30 to 80% B (10–35 min). Detection wavelength: 254 nm. CA indicates residual coniferyl alcohol. Compounds **1** and **2** are the β-O-4-coupled dimers whereas compound **3** is the β-5-coupled dimer of CA as shown in Figure 4.

On this basis, the reaction was repeated on a larger scale, and the products were isolated by simple TLC fractionation of the ethyl acetate extractable fraction and characterized by NMR analysis. This latter confirmed the identification of products **1** and **2** as the stereoisomeric guaiacylglycerol-β-coniferyl ethers,^52,54^ characterized by the presence of a β-O-4 linkage between the two phenolic units (Figures 4 and 5). Compound **3** was instead identified as the β-5 dehydrodimer with a *trans* configuration of the dihydrobenzofuran ring (Figures 4 and 5) by complete NMR analysis (Figures S5–S9), in comparison with data reported in literature.^52,54^

**Figure 4:**
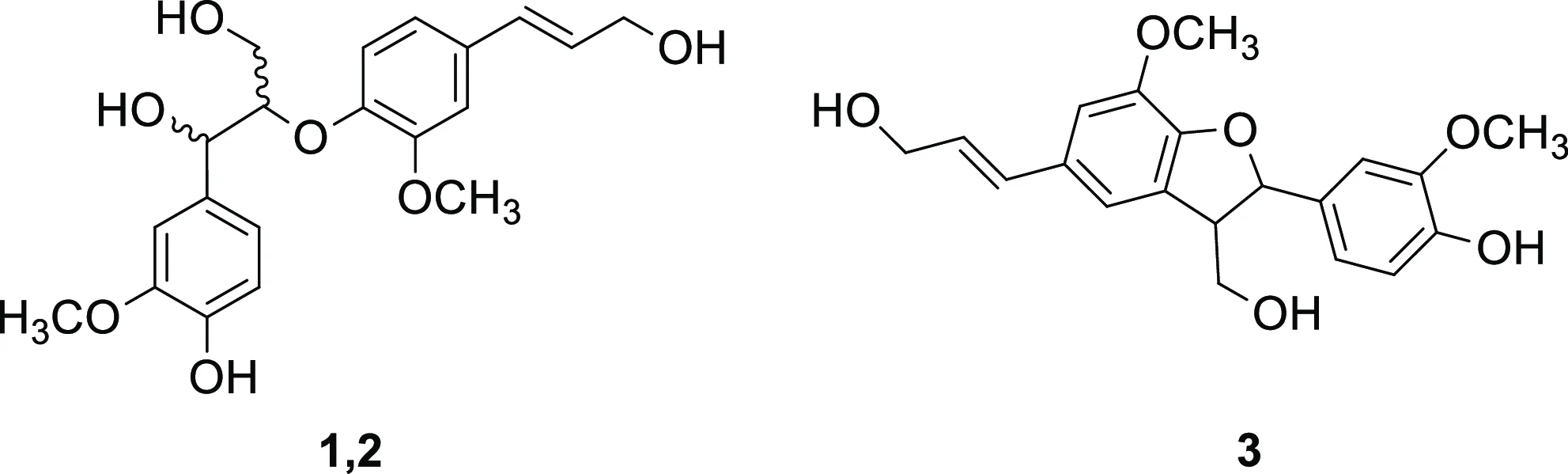
Structures of the main products of coniferyl alcohol (CA) oxidation induced by K_3_Fe­(CN)_6_.

**Figure 5:**
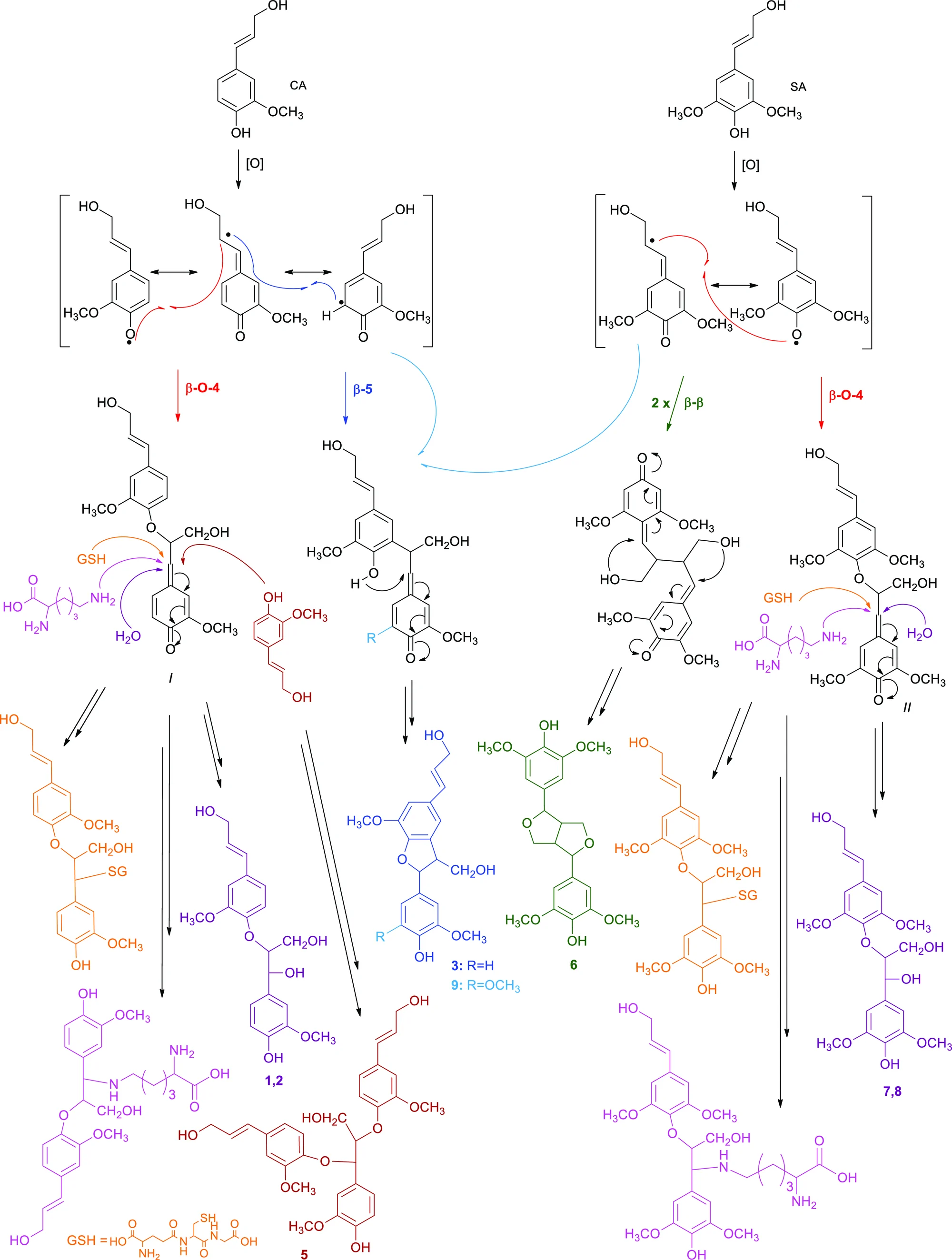
Proposed pathways for the formation of coniferyl alcohol (CA) and sinapyl alcohol (SA) oxidation products.

To further expand knowledge of the oxidation chemistry of CA, in another series of experiments its reactivity in the presence of other oxidizing systems was investigated. Initially, the oxidation reaction catalyzed by laccase, an enzyme also potentially involved in lignin biosynthesis and characterized by a lower optimal operating pH compared to HRP,^55–58^ was examined. In particular, the reaction was carried out using commercial laccase from *Trametes versicolor* at pH 3.0, at a CA concentration of 1 mM as above. The reaction mixture was monitored by LC-UV-MS analysis, and after 1 h formation of products **1-3** was detected. Notably, however, while with the HRP/H_2_O_2_ and K_3_Fe­(CN)_6_ systems at neutral pH the formation of the β-5 dehydrodimer **3** appeared to be favored over that of the β-O-4 dimers **1** and **2**, under acidic conditions these latter predominated, as clearly highlighted by the superimposed chromatographic profiles of the reaction mixtures shown in Figure S10.

To determine whether the different product distribution observed was attributable to a specific catalytic activity of laccase, additional experiments were performed in which the oxidation of CA was carried out at pH 3.0 using cerium­(IV) ammonium nitrate (CAN) as the oxidizing agent. Interestingly, also in this case, after 1 h formation of products **1** and **2** in higher amounts relative to product **3** was observed, as evidenced by the nearly perfect overlap of the chromatographic profiles of the two reaction mixtures run under acidic conditions (Figure S11).

Based on the experimental evidence collected, pH seems to significantly affect the CA dimerization pathway proposed in Figure 5. This observation can be rationalized considering that the rearomatization step proposed for the β-5 dimerization pathway may be disfavored under acidic conditions thereby promoting β-O-4 bond formation (Figure S12).

### Investigation of the Oxidative Chemistry of CA in Organic Solvents

To further explore alternative oxidation pathways of CA, additional experiments were performed in organic solvents using different oxidizing systems. LC-UV-MS analysis of the reaction mixture (carried out in methanol) of CA (1 mM) with DPPH (1 molar eq), a stable radical known to easily react with electron or hydrogen atom donors such as phenolic compounds, revealed after 1 h an almost complete consumption (94%) of the starting phenol, accompanied by the formation of two main products, one identified as the β-5 dehydrodimer **3** and another one (indicated as **4** in Figure S13) exhibiting a pseudomolecular ion peak [M-H]^‑^ at *m*/*z* 389, consistent with a CA dimer (more likely a mixture of coeluting diastereoisomeric dimers) analogous to the previously described β-O-4 dimers **1** and **2**, but arising from addition of methanol instead of water to the putative quinone methide intermediate *I* (see Figure 5). To verify this hypothesis, the reaction was repeated under the same conditions but in acetonitrile, a non-nucleophilic solvent, and as expected no detectable amounts of compound **4** were observed. Of course, under these non-aqueous conditions, formation of the β-O-4 dimers **1** and **2** was not observed, underscoring that their formation strictly requires the presence of water as nucleophile.

In further experiments, oxidation of CA was carried out in the presence of 2,2′-azobis­(isobutyronitrile) (AIBN), a compound able to act as a radical initiator upon thermal decomposition.^59^ Specifically, 1 molar eq of AIBN was added to a 1 mM solution of CA in acetonitrile. The mixture was kept under stirring at 60 °C and periodically monitored by LC-UV-MS analysis, which revealed a slower progression of the oxidation reaction compared to the above-described oxidizing systems. Nevertheless, after 24 h, a significant consumption of CA was observed (>95%), along with the formation of **3** as the major product (Figure S14). In a similar way, when manganese dioxide (MnO_2_) in dichloromethane^60^ was used as the oxidant, a complete consumption of CA was achieved after 96 h, with the exclusive formation of the β-5 dehydrodimer **3** (Figure S15). These conditions therefore provide a highly selective access route to this compound.

Finally, in other experiments the use of Ag_2_O as oxidizing agent was also evaluated following a protocol reported in literature^52^ in which CA was oxidized in the presence of 1.5 molar eqs of Ag_2_O in an acetone/sodium citrate buffer system at pH 2.5. However, no significant consumption of CA was detected by LC-UV-MS analysis, even at prolonged reaction times or upon increasing the amount of Ag_2_O.

### Investigation of the Oxidative Chemistry of CA at Solid-State

In another set of experiments, the solid-state reactivity of CA was investigated in the presence of the same oxidizing agents previously employed in solution, but in the absence of solvents by using mechanochemistry. This versatile synthetic methodology can promote distinct reactivity and selectivity compared to conventional solution-based reactions, thereby accessing otherwise unexplored chemical space. Moreover, mechanochemistry offers additional advantages such as improved control, shorter reaction times and the possibility of conducting reactions in the absence of potentially toxic organic solvents.^61–67^ Accordingly, the reactions were performed under solvent-free conditions, although organic solvents were subsequently employed for work-up and purification when required.

The oxidative chemistry of CA was initially investigated in a small-scale laboratory ball mill at a frequency of 50 oscillation/s in the presence of 1 molar eq of K_3_Fe­(CN)_6_. However, LC-UV-MS analysis revealed only minimal consumption of the starting material. To enhance the oxidizing activity of ferricyanide, which is typically more effective under neutral or alkaline conditions, the reaction was repeated in the presence of 5 molar eqs of sodium bicarbonate. Under these conditions, significant consumption (*ca.* 70%) of CA was observed after 30 min, accompanied by the formation of a major product indicated as **5** in Figure S16. This compound was characterized by a pseudomolecular ion peak [M-H]^‑^ at *m*/*z* 537 (Figure S16) and a distinct fragmentation peak [M-H]^‑^ at *m*/*z* 357, consistent with a trimeric derivative of CA which was absent in all the reaction mixtures carried out in solution. Other minor products, among which **3**, were characterized by pseudomolecular ion peaks [M-H]^‑^ at *m*/*z* 357, and, as expected, compounds **1** and **2** were not detected, further confirming the crucial role of water as nucleophile in directing this type of oxidative coupling.

When the solid-state reaction was carried out using CAN as oxidant, no products with well-defined chromatographic properties were detected, despite the almost complete consumption of CA, suggesting that under these conditions the oxidation proceeds without any product accumulation.

Conversely, in the presence of 1 molar eq of DPPH or MnO_2_, a product distribution very similar to that obtained with potassium ferricyanide/sodium bicarbonate was observed.

Remarkably, the most promising outcome was achieved when Ag_2_O (1 molar eq) was employed as oxidant. Indeed, in stark contrast to the lack of reactivity observed in solution, under solid-state conditions CA underwent complete conversion, affording almost exclusively product **5** (Figure 6).

**Figure 6:**
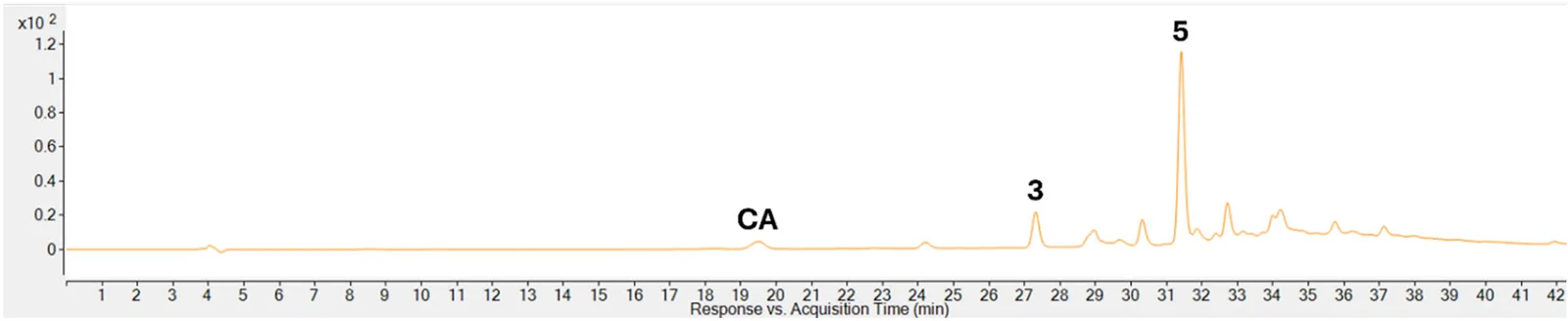
HPLC profile of the reaction mixture obtained by solid-state oxidation of coniferyl alcohol (CA) for 30 min in the presence of Ag_2_O (1 molar eq). Analysis was performed under the following conditions: Eclipse Plus C18 column (150 × 4.6 mm^2^, 5 μm). Eluent: 0.1% formic acid (solvent A)/methanol (solvent B): 30% B (0–10 min), from 30 to 80% B (10–35 min). Detection wavelength: 254 nm. CA indicates residual coniferyl alcohol. Compound **3** is the β-5-coupled dimer whereas compound **5** is the trimeric derivative of CA as shown in Figures 4 and 7, respectively.

After several attempts, a purification procedure to obtain the trimeric derivative in sufficient amount for structural characterization was developed. Specifically, 50 mg of CA were reacted with 2 molar eqs of Ag_2_O in the ball mill, and after 30 min the resulting powder was recovered in acetone and then subjected to centrifugation, to remove insoluble residual oxidant and products thereof, and fractionation by preparative TLC, which allowed the isolation of product **5** in pure form, enabling its complete NMR characterization. It should be noted that, when the reaction mixture was taken up in methanol instead of acetone, LC-MS analysis revealed a progressive degradation of **5** into **4** (the CA dimer conjugated with methanol), along with free CA.

The ^1^H NMR spectrum of **5** run in acetone-*d*_6_ (Figure S17) displayed the characteristic signals of an oxygenated propanol chain, that is a doublet at 5.48 ppm, a quartet at 4.58 ppm, and two signals at 3.94 and 3.87 ppm corresponding to two diastereotopic geminal protons. In the low-field region, signals attributable to nine protons typical of methoxyphenolic units were observed, along with two sets of signals characteristic of the propenoic side chain of CA and, at lower field, a single resonance corresponding to a phenolic OH group. Based on these data, on the complete analysis of the ^13^C and 2D NMR spectra (Figures S18–S21), and on the comparison with literature data,^52^ compound **5** was assigned the structure of guaiacylglycerol-α,β-bisconiferyl ether (Figure 7). It is worth noting that, similarly to guaiacylglycerol-β-coniferyl ether, product **5** can theoretically exist as two diastereoisomers (each as a pair of enantiomers). However, in this case, NMR analysis clearly indicated the presence of a single stereoisomer, specifically the *erythro* form in agreement with literature data.^52^

**Figure 7:**
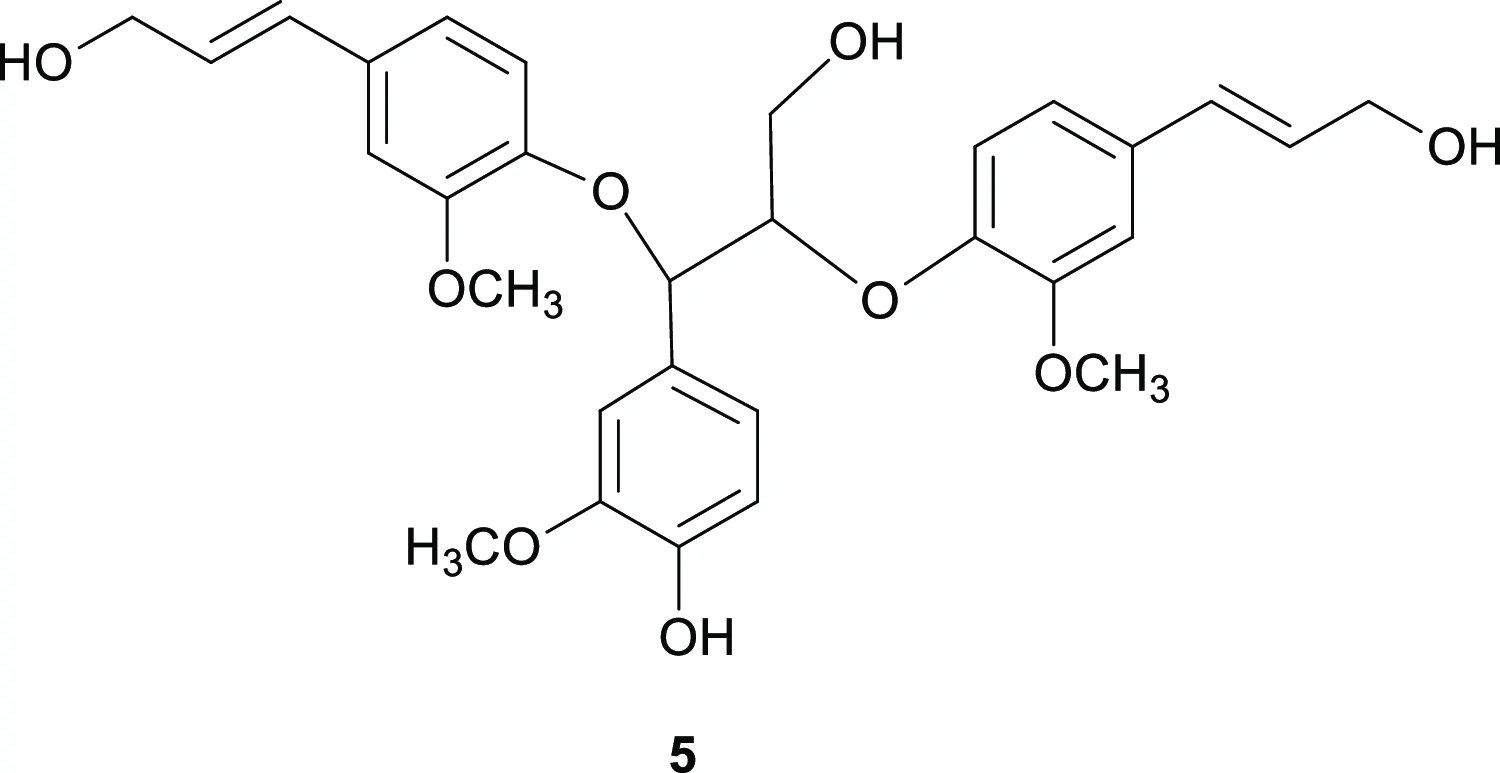
Structure of the guaiacylglycerol-α,β-bisconiferyl ether (**5**).

The formation of compound **5** can be tentatively rationalized through a pathway analogous to that proposed for the formation of the β-O-4 dimers **1** and **2** (Figure 5). Under solvent-free conditions, CA would represent the only species available to act as a nucleophile toward the putative quinone methide intermediate *I*, through is phenolic OH group. The proposed structure of compound **5** is also consistent with its marked instability in methanol (see above), since the CA unit at the α-position may act as an excellent leaving group in the presence of other nucleophilic species (Figure S22). Its elimination should be also facilitated by the push exerted by the free phenolic OH group. Moreover, when compound **5** was kept in phosphate buffer at pH 6.8, that is under the conditions leading to the formation of **1** and **2** as described above, a 61% consumption was observed within 1 h, accompanied by the concomitant release of CA and formation of **1** and **2** (Figure S23).

### Investigation of the Oxidative Chemistry of PCA and SA

Given the relevant role of PCA and especially SA as lignin precursors, their oxidative chemistry was also investigated to gain mechanistic insights into the different oxidative coupling pathways that ultimately lead to lignin.

Under the same conditions employed for CA, PCA displayed a very limited reactivity in solution, even in the presence of an excess of the oxidizing reagents. On the contrary a very high PCA consumption (>80%) was observed under solid-state conditions in the presence of Ag_2_O or sodium bicarbonate/K_3_Fe­(CN)_6_ mixture. However, only traces of chromatographable reaction products (mainly dimeric compounds) were detected, and therefore these reactions were not further investigated.

As far as SA is concerned, it was found to be very reactive both in solution and under solid-state conditions. As an example, LC-UV-MS analysis of the reaction mixture of SA with HRP/H_2_O_2_, K_3_Fe­(CN)_6_ or laccase indicated a consumption of the phenolic compound of *ca.* 58%, 98% and 95% in that order after 1 h, with the formation of a main product indicated as **6** in Figure 8. This compound was characterized by a pseudomolecular ion peak [M-H]^‑^ at *m*/*z* 417 (Figure S24), consistent with a dimeric derivative of SA. Surprisingly, no β-O-4 dimers analogous to products **1** and **2** of CA were detected under these conditions.

**Figure 8:**
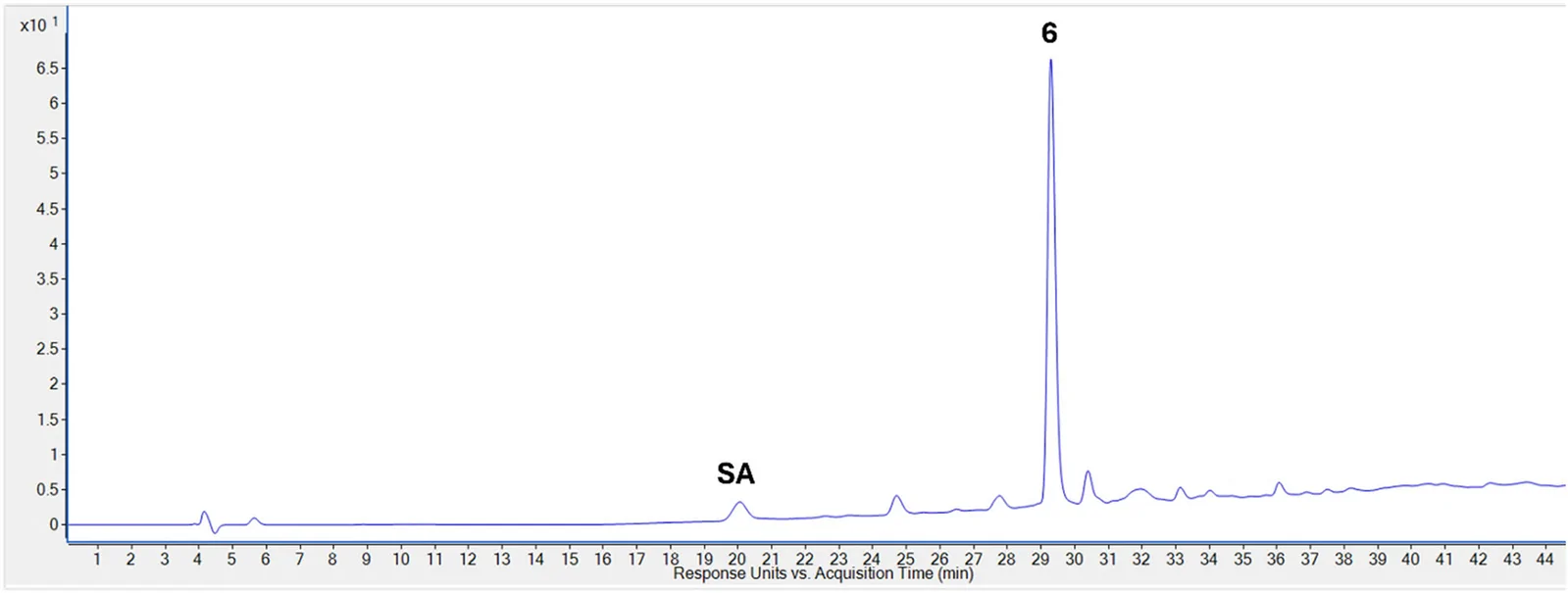
HPLC profile of the reaction mixture of sinapyl alcohol (SA) in the presence of K_3_Fe­(CN)_6_ (1 molar eq) in phosphate buffer at pH 6.8 after 1 h. Analysis was performed under the following conditions: Eclipse Plus C18 column (150 × 4.6 mm^2^, 5 μm). Eluent: 0.1% formic acid (solvent A)/methanol (solvent B): 30% B (0–10 min), from 30 to 80% B (10–35 min). Detection wavelength: 254 nm. SA indicates residual sinapyl alcohol. Compound **6** is the β-β-coupled dimer of SA as shown in Figure 9.

To get an insight into the structure of compound **6**, the reaction of SA with K_3_Fe­(CN)_6_ was repeated on a larger scale and the product was isolated by TLC fractionation of the ethyl acetate extractable fraction of the reaction mixture. ^1^H NMR analysis, in comparison with that reported in literature,^68^ allowed to formulate **6** as the β-β-coupled dimer syringaresinol (Figures 5 and 9). Notably, no significant amounts of a β-β-coupled dimer was detected in the case of CA under any of the reaction conditions explored.

**Figure 9:**
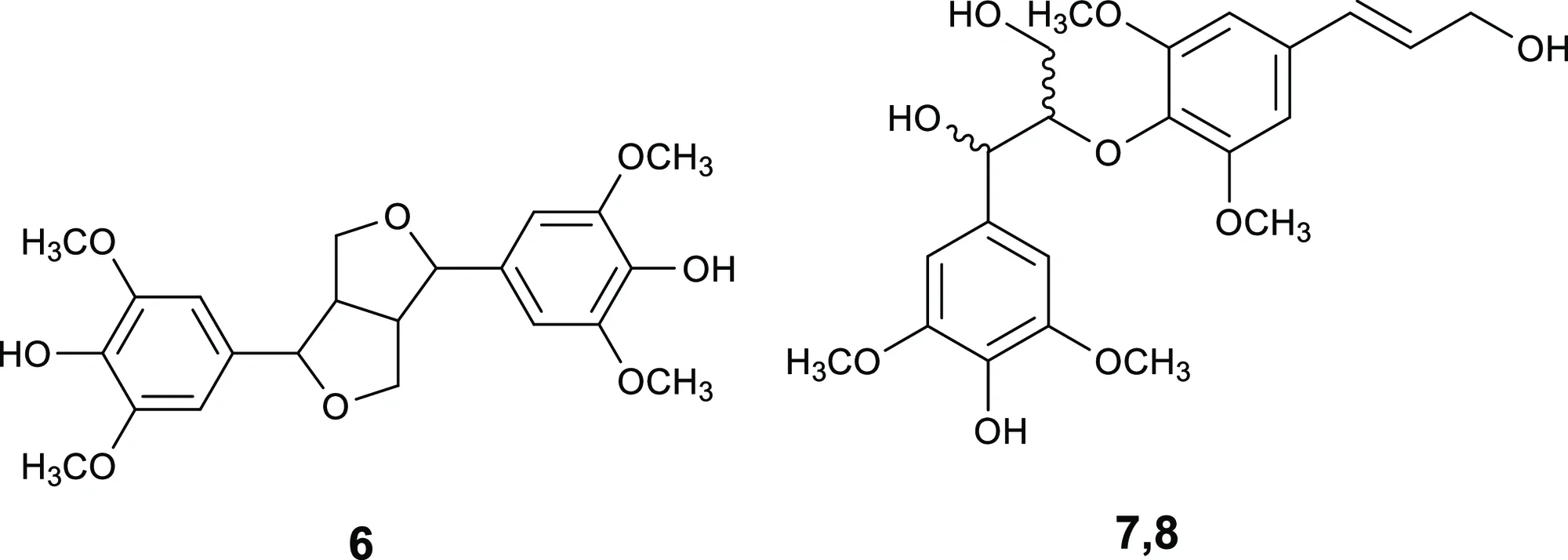
Structures of syringaresinol (**6**) and syringylglycerol-β-sinapyl ethers (**7** and **8**).

These findings highlight how structural differences among lignin precursors may strongly affect their oxidative coupling pathways. The very low reactivity of PCA may reflect the absence of methoxy substituents on the aromatic ring, which reduces electron density and radical stabilization, thereby hindering oxidation. In contrast, the presence of an additional methoxy group in SA compared to CA may not only increase its reactivity under oxidative conditions but also induce different coupling routes, preventing formation not only of β-5 linkages (as expected based on the substitution pattern of SA) but surprisingly also of the β-O-4 linkages. This observation suggests that the wide occurrence of β-O-4 linkages in natural lignin must be attributed to the primary involvement of CA-related units in the biosynthetic process.

Further exploring the oxidative chemistry of SA, no products with well-defined chromatographic properties were detected when oxidation was run in organic solvents with MnO_2_ or AIBN, or under solid-state conditions with Ag_2_O or the K_3_Fe­(CN)_6_/sodium bicarbonate system, despite the almost complete consumption of the starting phenol.

Notably, however, when SA was reacted at a concentration of 62 mM for 1 h in a 5:2 v/v dioxane/water or acetone/water mixture in the presence of FeCl_3_ (0.8 molar eqs) following a protocol previously reported in literature,^69^ LC-UV-MS analysis showed the formation, besides of **6**, of two main products (indicated as **7** and **8** in Figure S25) both exhibiting a pseudomolecular ion peak [M-H]^‑^ at *m*/*z* 435 (Figure S25) consistent with β-O-4-type dimers analogous to **1** and **2**. ^1^H NMR analysis of compounds **7** and **8**, isolated as a 2.5:1 mixture from a larger scale reaction, confirmed their assignment as the stereoisomeric siryngylglycerol-β-sinapyl ethers (Figures 5 and 9). Based on literature data^68^ it was possible to assign the *erythro* configuration to the main diastereoisomer.

Formation of compounds **7** and **8** only in the presence of dioxane or acetone has been ascribed to the strong contribution of the inductive effect exerted by the methoxyl groups in low polar solvent mixtures.^69^ However, it is worth noting that when the reaction was conducted at 1 mM SA concentration in the presence of FeCl_3_, or when K_3_Fe­(CN)_6_ was used as the oxidant in the dioxane/water or acetone/water mixtures, no formation (or only trace amount) of the β-O-4-type dimers was observed. On the other hand, oxidation of 62 mM CA in the presence of FeCl_3_ in the 5:2 v/v dioxane/water mixture led to the formation of only low amounts of products **1** and **2**. All these findings thus suggest that not only the chemical structure of the precursor but also the reaction environment can strongly affect the structure of the resulting lignin polymer.

Finally, in order to investigate how the three lignin precursors behave when oxidized together, and to more closely reproduce the natural scenario of lignin biosynthesis, in other experiments the three hydroxycinnamyl alcohols CA, PCA, and SA were oxidized together in solution at pH 6.8 with K_3_Fe­(CN)_6_. Initially, the reaction was performed with each alcohol at a concentration of 1 mM in the presence of 1 mM oxidant. In agreement with the observations described above, LC-UV-MS analysis after 1 h revealed a significant consumption of SA (76%), whereas CA and PCA were only slightly affected, showing a 5% and 3% consumption, respectively.

The experiment was thus repeated using 3 mM K_3_Fe­(CN)_6_. Under these conditions, while PCA remained essentially unreacted (only 2% consumption), a complete and marked (*ca.* 80%) consumption was observed for SA and CA respectively, accompanied by the formation of the SA β-β-coupled dimer syringaresinol (**6**) and of a new product (indicated as **9** in Figure S26), characterized by a pseudomolecular ion peak [M-H]^‑^ at *m*/*z* 387 (Figure S26), indicative of a heterodimer derived from CA and SA. A comparable product distribution was obtained when the oxidation was restricted to CA and SA, given the lack of reactivity of PCA, and under more biomimetic conditions using the HRP/H_2_O_2_ system.

These findings suggest that when the two precursors CA and SA are oxidized together, cross-coupling pathways are favored over homocoupling, at least under the conditions investigated, thereby providing a possible contribution to the structural heterogeneity observed in natural lignins, where mixed linkages between different monolignols are commonly found.

^1^H NMR analysis, in comparison with literature data,^54^ of compound **9** isolated by TLC fractionation of a larger scale reaction mixture, enabled its structural assignment as a β-5 type dimer (simulanol) (Figure 10). The formation of this dimer is consistent with the oxidative cross-coupling pathway shown in Figure 5, in which the β-carbon of the SA radical selectively couples with the unsubstituted C-5 position of the aromatic ring of the CA radical.

**Figure 10:**
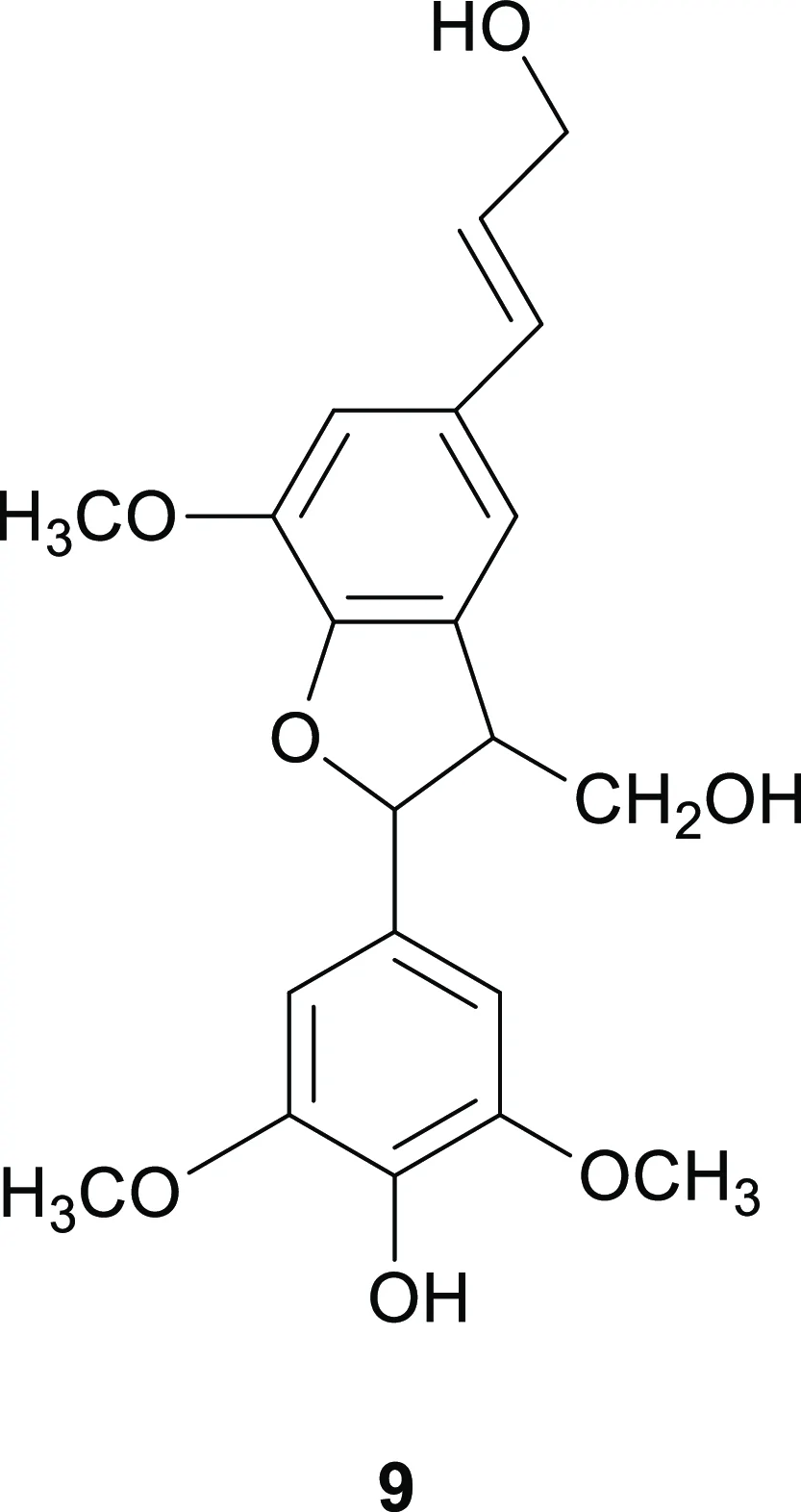
Structure of simulanol (**9**).

### Exploration of the Oxidative Chemistry of CA and SA in the Presence of Nucleophiles

The formation of β-O-4 dimers and trimer of CA (compounds 1, 2, 4, and 5), as well as of the β-O-4 dimer of SA (compounds 7 and 8), is consistent with the strong reactivity of the oxidation intermediates of both compounds, mostly the putative quinone methide intermediates I and II (Figure 5), toward nucleophiles. This reactivity offers an attractive opportunity to design novel derivatives of CA and SA which could ultimately give rise to functional, lignin-inspired materials with potential value-added properties. In this context, the study was therefore extended to the oxidative coupling reactions of CA and SA with nitrogen- and sulfur-based nucleophiles, namely hexamethylenediamine (HMDA), lysine and glutathione (GSH). The choice of these nucleophiles was motivated by their chemical and functional features. For instance, HMDA was selected as a simple aliphatic diamine, providing two primary amino groups that could potentially enable subsequent cross-linking reactions. Lysine, a naturally occurring amino acid, was selected as a biobased nucleophile capable of introducing additional amino functionalities and potentially enhancing the water solubility of the resulting derivatives. GSH was selected as a representative natural sulfur-containing nucleophile, which could also contribute to the hydrophilic character of the resulting conjugates. The specific aim was therefore to obtain new, water-soluble and functional lignin precursors, bearing *e.g.,* free amino and/or thiol groups available for further functionalization or derivatization.

When CA was oxidized in the presence of HMDA (5 molar eqs) and K_3_Fe­(CN)_6_ (1 molar eq), a 90% consumption of CA was observed after 1 h. Besides the formation of the β-5 dimer **3** and minor amounts of the β-O-4 dimers **1** and **2**, two additional products eluting at 6.5 and 9.5 min were detected, both showing pseudomolecular ion peaks [M-H]^‑^ at *m*/*z* 473 consistent with CA-HMDA conjugates analogous to the water or methanol conjugates **1**, **2**, and **4** (Figure S27).

A similar result was obtained when the reaction was run in the presence of lysine (10 molar eqs) and K_3_Fe­(CN)_6_ (1 molar eq), leading to the formation of four products eluting between 8 and 17 min (Figure 11), all exhibiting pseudomolecular ion peaks [M-H]^‑^ at *m*/*z* 503 (Figure S28) compatible with CA-lysine conjugates (Figure 5). The formation of four products suggests the involvement of both amino groups of lysine in the coupling. In agreement with this hypothesis, when the reaction was conducted using Nα-acetyl-l-lysine methyl ester, a lysine derivative protected on the α-amino group, only two products were detected, eluting at 13.5 and 18.5 min and exhibiting pseudomolecular ion peaks compatible with a conjugate formed between CA and the hydrolyzed lysine derivative at the ester moiety as a result of the strong alkaline conditions adopted ([M-OCH_3_ + H_2_O -H]^‑^ at *m*/*z* 545) (Figure S29).

**Figure 11:**
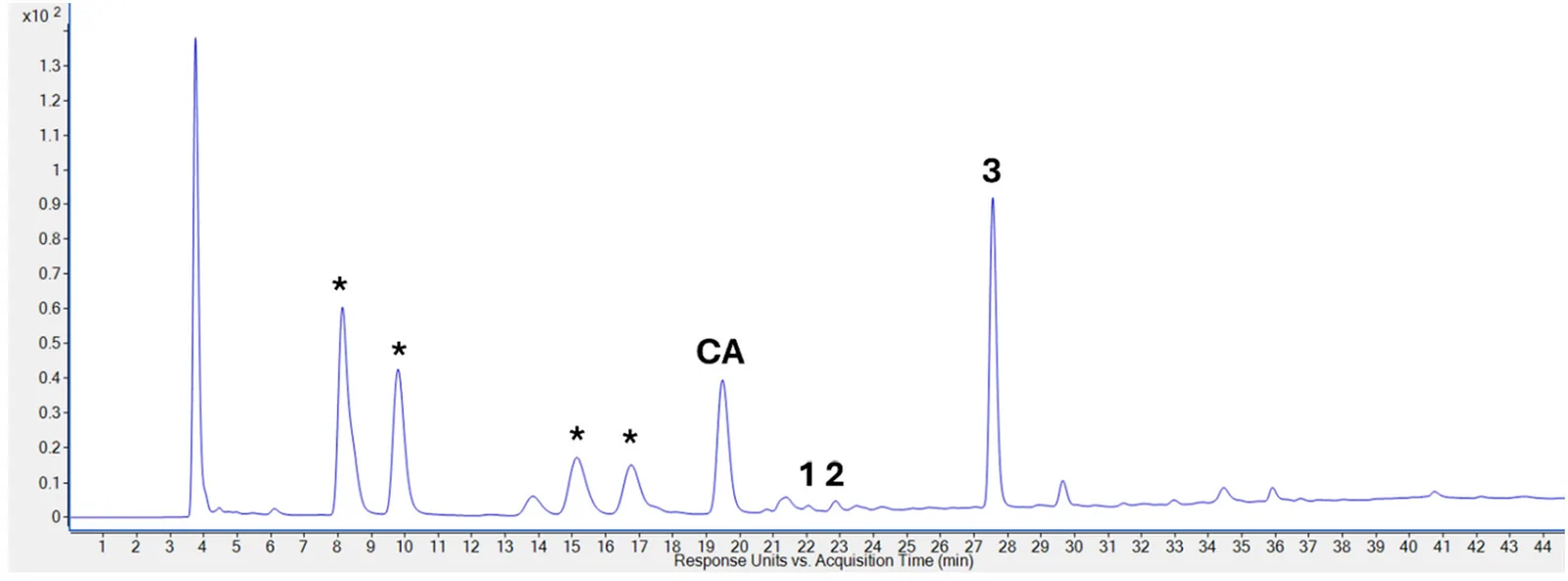
HPLC profile of the reaction mixture of coniferyl alcohol (CA) in the presence of lysine (10 molar eqs) and K_3_Fe­(CN)_6_ (1 molar eq). Analysis was performed under the following conditions: Eclipse Plus C18 column (150 × 4.6 mm^2^, 5 μm). Eluent: 0.1% formic acid (solvent A)/methanol (solvent B): 30% B (0–10 min), from 30 to 80% B (10–35 min). Detection wavelength: 254 nm. Asterisks indicate the CA-lysine conjugates. CA indicates residual coniferyl alcohol. Compounds **1** and **2** are the β-O-4-coupled dimers whereas compound **3** is the β-5-coupled dimer of CA as shown in Figure 4.

When GSH (5 molar eqs) was used as nucleophile with the HRP/H_2_O_2_ oxidizing system, a 70% consumption of CA was observed after 1 h, along with the formation of the β-5 dimer **3** and two additional products coeluting at *ca.* 22 min, characterized by a pseudomolecular ion peak [M-H]^‑^ at *m*/*z* 664 consistent with a CA-GSH conjugate (Figure S30, Figure 5).

Comparable reactivity was observed for SA. In particular, in the presence of lysine (10 molar eqs) and K_3_Fe­(CN)_6_ (1 molar eq), SA showed a 66% consumption after 1 h and afforded, besides the syringaresinol **6**, four minor products eluting between 14 and 19 min, displaying pseudomolecular ion peaks [M-H]^‑^ at *m*/*z* 563 all consistent with SA-lysine conjugates (Figure S31, Figure 5). In the case of GSH, using the HRP/H_2_O_2_ oxidizing system, a 50% consumption of SA was observed, together with the formation of two products eluting at 23 and 24 min with a pseudomolecular ion peak [M-H]^‑^ at *m*/*z* 724 in line with diastereoisomeric SA-GSH conjugates (Figures 5 and 12 and Figure S32).

**Figure 12:**
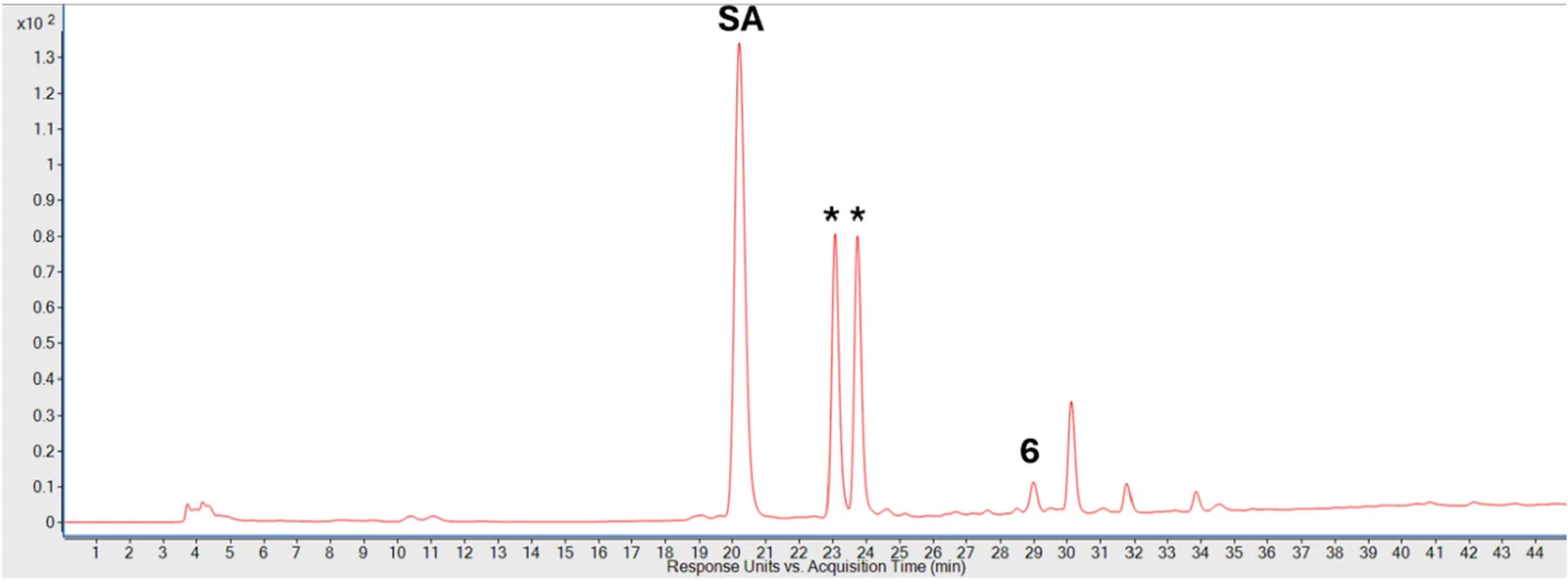
HPLC profile (UV detection at 254 nm) of the reaction mixture of sinapyl alcohol (SA) in the presence of GSH (5 molar eqs) and HRP (1 U/mL)/H_2_O_2_ (0.2 molar eqs). Analysis was performed under the following conditions: Eclipse Plus C18 column (150 × 4.6 mm^2^, 5 μm). Eluent: 0.1% formic acid (solvent A)/methanol (solvent B): 30% B (0–10 min), from 30 to 80% B (10–35 min). Detection wavelength: 254 nm. SA indicates residual sinapyl alcohol. Asterisks indicate the SA-GSH conjugates. Compound **6** is the β-β-coupled dimer of SA as shown in Figure 9.

Although these products have not yet been isolated and fully characterized, their formation demonstrates the ability of CA and SA oxidation intermediates to react with nucleophiles, paving the way toward the synthesis of modified lignin precursors. Such conjugates may not only yield water-soluble lignin-like polymers upon further oxidation, but could also serve as cross-linkable building blocks, owing to the presence of free amino groups.

## An Overview of the Proposed Pathways for the Oxidation of CA and SA under the Conditions Explored in this Study is Reported in Figure 5

In conclusion, by combining conventional and mechanochemical oxidation approaches, this study provides not only a comprehensive overview of the oxidative pathways of CA, SA, and PCA for a deeper understanding of the complex lignin biosynthetic process, but also novel information to specifically access lignan compounds. Indeed, for coniferyl alcohol, it has been demonstrated that it is possible to achieve controlled access to specific products by proper selection of the oxidant and the reaction medium.

Particularly relevant is the observation that when CA and SA were oxidized together, oxidative cross-coupling was favored over homocoupling, leading to the formation of a CA-SA heterodimer. This finding may contribute to explain the structural heterogeneity of natural lignins, where different monolignols are connected through a variety of interunit linkages.

Noteworthy is also the investigation of the solid-state oxidative reactivity of coniferyl alcohol, highlighting the potential of mechanochemistry as a green and efficient alternative to conventional solvent-based processes, selectively affording a trimeric guaiacylglycerol-α,β-bisconiferyl ether derivative that is not accessible under solution conditions.

Moreover, the successful formation of different nucleophile-coniferyl alcohol/sinapyl alcohol conjugates offers new perspectives for the synthesis of lignin-inspired compounds with tailored functionality and solubility. Overall, these advances open new versatile and sustainable avenues for the development of lignin-inspired functional materials.

## Supplementary Material



## Notes

Materials and methods; experimental details; and LC-UV-MS and NMR analyses (PDF)

## Abbreviations

CA: coniferyl alcohol
SA: sinapyl alcohol
PCA: *p*-coumaryl alcohol
DPPH: 2,2-diphenyl-1-picrylhydrazyl
GSH: glutathione
H_2_O_2_: hydrogen peroxide
K_3_Fe­(CN)_6_: potassium ferricyanide
FeCl_3_: iron chloride
HRP: horseradish peroxidase
CAN: cerium­(IV) ammonium nitrate
Ag_2_O: silver­(I) oxide
AIBN: 2,2′-azobis­(isobutyronitrile)
MnO_2_: manganese dioxide

## References

[1] Boerjan W., Ralph J., Baucher M. (2003). Lignin Biosynthesis. Annu. Rev. Plant Biol..

[2] Liu Q., Luo L., Zheng L. (2018). Lignins: Biosynthesis
and Biological Functions in Plants. Int. J.
Mol. Sci..

[3] Ana L. M., Rogelio S., Xose Carlos S., Rosa Ana M. (2022). Cell Wall Composition
Impacts Structural Characteristics of the Stems and Thereby the Biomass
Yield. J. Agric. Food Chem..

[4] Nishimura H., Kamiya A., Nagata T., Katahira M., Watanabe T. (2018). Direct Evidence
for α Ether Linkage between Lignin and Carbohydrates in Wood
Cell Walls. Sci. Rep..

[5] Saake, B. ; Lehnen, R. L. Ullmann’s Encyclopedia of Industrial Chemistry 2007.

[6] Yan L., Huertas-Alonso A. J., Liu H., Dai L., Si C., Sipponen M. H. (2025). Lignin Polymerization: Towards High-Performance Materials. Chem. Soc. Rev..

[7] Liu H., Guan Y., Yan L., Zheng Y., Si C., Dai L. (2024). The Development of Lignin towards a Natural and Sustainable Platform
for Optical Materials. Green Chem..

[8] Zhou H., Yan L., Tang D., Xu T., Dai L., Li C., Chen W., Si C. (2024). Solar-Driven
Drum-Type Atmospheric
Water Harvester Based on Bio-Based Gels with Fast Adsorption/Desorption
Kinetics. Adv. Mater..

[9] Guan Y., Liu H., Han Y., Zhou Y., Yan L., Dai L., Si C. (2024). Unlocking
the Photothermal Conversion Capacity of Lignin and Lignin-Derived
Materials. Chem. Eng. J..

[10] Gaikwad K. K. A. (2024). Lignin
as a UV Blocking, Antioxidant, and Antimicrobial Agent for Food Packaging
Applications. Biomass Conv. Bioref..

[11] Zubair M., Rauf Z., Fatima S., Ullah A. (2024). Lignin-Derived Bionanocomposites
as Functional Food Packaging Materials. Sustainable
Food Technol..

[12] Wu T., Sugiarto S., Yang R., Sathasivam T., Weerasinghe U. A., Chee P. L., Yap O., Nyström G., Kai D. (2025). From 3D to 4D Printing of Lignin
towards Green Materials and Sustainable
Manufacturing. Mater. Horiz..

[13] Grappa R., Venezia V., Basta L., Alfieri M. L., Panzella L., Verrillo M., Silvestri B., Commodo M., Luciani G., Costantini A. (2024). Green Synthesis
of Stable Lignin Nanoparticles with
Reversible Swelling Behavior and Enhanced Multifunctional Features. ACS Sustainable Chem. Eng..

[14] Shan Z., Jiang B., Wang P., Wu W., Jin Y. (2025). Sustainable
Lignin-Based Composite Hydrogels for Controlled Drug Release and Self-Healing
in Antimicrobial Wound Dressing. Int. J. Biol.
Macromol..

[15] Liu R., Li S., Liu S., Li J., Chen Z., James T. D. (2025). Convenient
Production of Photothermal Recycling Phosphorescent Materials from
Cellulose and Lignin. Angew. Chem., Int. Ed..

[16] Pang T., Xue Z., Wang G., Li J., Sui W., Si C. (2025). Non-Carbonized
Pd Single-Atom Catalyst Supported on Lignin-Functionalized Phenolic
Resin for Potent Catalytic Transfer Hydrogenation of Lignin-Derived
Aldehydes. Angew. Chem., Int. Ed..

[17] Abolore R. S., Jaiswal S., Jaiswal A. K. (2025). A Comprehensive Review on Sustainable
Lignin Extraction Techniques, Modifications, and Emerging Applications. Ind. Crop. Prod..

[18] Hu M., Yu Y., Li X., Wang X., Liu Y. (2023). The Dawn of Aqueous
Deep Eutectic Solvents for Lignin Extraction. Green Chem..

[19] Gómez-Cruz I., Sosa F., Alfieri M. L., Esposito R., Panzella L., Labidi J., Goodfellow B., Castro E., Silvestre A. J. D., da Costa Lopes A. M. (2025). Evaluating the Integration of Olive
Tree Pruning Fractionation with Ternary Eutectic Solvent after Aqueous
Pre-Extraction or Dilute Acid Pre-Treatment. Sep. Purif. Technol..

[20] Sheridan E., Filonenko S., Volikov A., Sirviö J. A., Antonietti M. (2024). A Systematic Study on the Processes of Lignin Extraction
and Nanodispersion to Control Properties and Functionality. Green Chem..

[21] Bourmaud C. L., Bertella S., Rico A. B., Karlen S. D., Ralph J., Luterbacher J. S. (2024). Quantification of Native Lignin Structural Features
with Gel-Phase 2D-HSQC0 Reveals Lignin Structural Changes During Extraction. Angew. Chem., Int. Ed..

[22] Ralph J., Lapierre C., Boerjan W. (2019). Lignin Structure
and Its Engineering. Curr. Opin. Biotechnol..

[23] Lahive C. W., Kamer P. C. J., Lancefield C. S., Deuss P. J. (2020). An Introduction
to Model Compounds of Lignin Linking Motifs; Synthesis and Selection
Considerations for Reactivity Studies. ChemSusChem.

[24] Ralph J., Lundquist K., Brunow G., Lu F., Kim H., Schatz P. F., Marita J. M., Hatfield R. D., Ralph S. A., Christensen J. H., Boerjan W. (2004). Lignins: Natural Polymers from Oxidative
Coupling of 4-Hydroxyphenyl- Propanoids. Phytochem.
Rev..

[25] Vanholme R., Demedts B., Morreel K., Ralph J., Boerjan W. (2010). Lignin Biosynthesis
and Structure. Plant Physiol..

[26] Zakzeski J., Bruijnincx P. C. A., Jongerius A. L., Weckhuysen B. M. (2010). The Catalytic
Valorization of Lignin for the Production of Renewable Chemicals. Chem. Rev..

[27] Morreel K., Dima O., Kim H., Lu F., Niculaes C., Vanholme R., Dauwe R., Goeminne G., Inzé D., Messens E., Ralph J., Boerjan W. (2010). Mass Spectrometry-Based
Sequencing of Lignin Oligomers. Plant Physiol..

[28] Dixon R. A., Barros J. (2019). Lignin Biosynthesis:
Old Roads Revisited and New Roads
Explored. Open Biol..

[29] Morreel K., Ralph J., Kim H., Lu F., Goeminne G., Ralph S., Messens E., Boerjan W. (2004). Profiling of Oligolignols
Reveals Monolignol Coupling Conditions in Lignifying Poplar Xylem. Plant Physiol..

[30] Erdtman, H. Lignins: Occurrence, Formation, Structure and Reactions. In J. Polym. Sci., Part B: Polym. Lett.; Sarkanen, K. V. ; Ludwig, C. H. , Eds.; John Wiley & Sons, Inc.: New York, 1972; Vol. 10, pp 228–230.

[31] Li Q., Hu C., Li M., Truong P., Li J., Lin H. S., Naik M. T., Xiang S., Jackson B. E., Kuo W., Wu W., Pu Y., Ragauskas A. J., Yuan J. S. (2021). Enhancing the Multi-Functional
Properties of Renewable Lignin Carbon Fibers via Defining the Structure–Property
Relationship Using Different Biomass Feedstocks. Green Chem..

[32] de
Oliveira Gorgulho Silva C., Bapat N. A., Bourmaud C. L., Nørskov Jensen C., de Bueren J. B., Luterbacher J., Meyer A. S., van Erven G., van Berkel W. J. H., Kabel M. A., W Agger J. (2026). Oxygenation and Oxidation of Lignin
Model Dimers by Fungal Ortho-Methoxyphenolases. J. Am. Chem. Soc..

[33] Bapat N. A., Bourmaud C. L., Rolland M., Luterbacher J. S. (2026). A Simple
Chlorination Pathway to Access Native-Like β-O-4 Lignin and
Monolignols. J. Agric. Food Chem..

[34] Antenucci S., Panzella L., Farina H., Ortenzi M. A., Caneva E., Martinotti S., Ranzato E., Burlando B., d’Ischia M., Napolitano A., Verotta L. (2016). Powering Tyrosol Antioxidant Capacity
and Osteogenic Activity by Biocatalytic Polymerization. RSC Adv..

[35] Alfieri M. L., Moccia F., D’Errico G., Panzella L., d’Ischia M., Napolitano A. (2020). Acid Treatment Enhances the Antioxidant Activity of
Enzymatically Synthesized Phenolic Polymers. Polymers.

[36] Alfieri M. L., Gonçalves C., R Almeida J., Correia-da-Silva M., Panzella L., Napolitano A. (2024). Sulfated Phenolic
Polymers as Non-Toxic
Antifouling Agents. Eur. Polym. J..

[37] Alfieri M. L., Panzella L., Duarte B., Goncalves-Monteiro S., Marques F., Morato M., Correia-Da-Silva M., Verotta L., Napolitano A. (2021). Sulfated Oligomers of Tyrosol: Toward
a New Class of Bioinspired Nonsaccharidic Anticoagulants. Biomacromolecules.

[38] Giummarella N., Gioia C., Lawoko M. (2018). A One-Pot Biomimetic
Synthesis of
Selectively Functionalized Lignins from Monomers: A Green Functionalization
Platform. Green Chem..

[39] Reale S., Attanasio F., Spreti N., De Angelis F. (2010). Lignin Chemistry:
Biosynthetic Study and Structural Characterisation of Coniferyl Alcohol
Oligomers Formed In Vitro in a Micellar Environment. Chem. - Eur. J..

[40] Okusa K., Miyakoshi T., Chen C. L. (1996). Comparative Studies on Dehydrogenative
Polymerization of Coniferyl Alcohol by Laccases and Peroxidases: Part
1. Preliminary Results. Holzforschung.

[41] Ralph J. (2010). Hydroxycinnamates
in Lignification. Phytochem. Rev..

[42] Nishimoto T., Matsushita Y. (2025). Reactivity
of p-Hydroxyphenyl Units in Enzymatic Co-Oxidative
Coupling of p-Coumaryl and Coniferyl Alcohols. J. Agric. Food Chem..

[43] de
Oliveira Gorgulho Silva C., Sun P., Barrett K., Sanders M. G., van Berkel W. J. H., Kabel M. A., Meyer A. S., Agger J. W. (2024). Polyphenol Oxidase Activity on Guaiacyl and Syringyl
Lignin Units. Angew. Chem., Int. Ed..

[44] Yamaguchi A., Senda K., Urabe D., Kishimoto T. (2026). Initial Stage
of Guaiacyl–Syringyl Lignin Formation from Coniferyl and Sinapyl
Alcohols. J. Agric. Food Chem..

[45] Panzella L., Greca M. D., Longobardo L. (2018). A Facile Preparation
of Hydroxycinnamyl
Alcohols With Simultaneous Protection of Phenol Groups as Carbonate. ChemistrySelect.

[46] Méchin V., Baumberger S., Pollet B., Lapierre C. (2007). Peroxidase Activity
Can Dictate the in Vitro Lignin Dehydrogenative Polymer Structure. Phytochemistry.

[47] Sasaki S., Nishida T., Tsutsumi Y., Kondo R. (2004). Lignin Dehydrogenative
Polymerization Mechanism: A Poplar Cell Wall Peroxidase Directly Oxidizes
Polymer Lignin and Produces in Vitro Dehydrogenative Polymer Rich
in β-O-4 Linkage. FEBS Lett..

[48] Warinowski T., Koutaniemi S., Kärkönen A., Sundberg I., Toikka M., Simola L. K., Kilpeläinen I., Teeri T. H. (2016). Peroxidases Bound
to the Growing Lignin Polymer Produce
Natural like Extracellular Lignin in a Cell Culture of Norway Spruce. Front. Plant Sci..

[49] Halliwell B. (1978). Lignin Synthesis:
The Generation of Hydrogen Peroxide and Superoxide by Horseradish
Peroxidase and Its Stimulation by Manganese (II) and Phenols. Planta.

[50] Chioccara F., Poli S., Rindone B., Pilati T., Brunow G., Pietikäinen P., Setälä H., Trætteberg M., Nasiri A., Tsuda T. (1993). Regio- and Diastereo-Selective Synthesis
of Dimeric Lignans Using Oxidative Coupling. Acta Chem. Scand..

[51] Holmgren A., Zhang L., Henriksson G. (2008). Monolignol
Dehydrogenative Polymerization
in Vitro in the Presence of Dioxane and a Methylated β-B′
Dimer Model Compound. Holzforschung.

[52] Quideau S., Ralph J. A. (1994). Biomimetic Route
to Lignin Model Compounds via Silver
(I) Oxide Oxidation: 1. Synthesis of Dilignols and Non-Cyclic Benzyl
Aryl Ethers. Holzforschung.

[53] Peng J., Lu F., Ralph J. (1998). The DFRC Method
for Lignin Analysis. 4. Lignin Dimers
Isolated from DFRC-Degraded Loblolly Pine Wood. J. Agric. Food Chem..

[54] Sally, A. ; Ralph, J. R. ; Lu, F. NMR Database of Lignin and Cell Wall Model Compounds. 2024 https://www.glbrc.org/databases_and_software/nmrdatabase/.

[55] Zhao Q., Nakashima J., Chen F., Yin Y., Fu C., Yun J., Shao H., Wang X., Wang Z. Y., Dixon R. A. (2013). Laccase
Is Necessary and Nonredundant with Peroxidase for Lignin Polymerization
during Vascular Development in Arabidopsis. Plant Cell.

[56] Wang J., Feng J., Jia W., Chang S., Li S., Li Y. (2015). Lignin Engineering through Laccase Modification: A Promising Field
for Energy Plant Improvement. Biotechnol. Biofuels.

[57] O’Malley D. M., Whetten R., Bao W., Chen C. L., Sederoff R. R. (1993). The Role
of of Laccase in Lignification. Plant J..

[58] Kishimoto T., Hiyama A., Toda H., Urabe D. (2022). Effect of PH on the
Dehydrogenative Polymerization of Monolignols by Laccases from Trametes
Versicolor and Rhus Vernicifera. ACS Omega.

[59] Krishnamachari V., Levine L. H., Paré P. W. (2002). Flavonoid
Oxidation by the Radical
Generator AIBN: A Unified Mechanism for Quercetin Radical Scavenging. J. Agric. Food Chem..

[60] Panzella L., Manini P., Napolitano A., d’Ischia M. (2004). Free Radical
Oxidation of (E)-Retinoic Acid by the Fenton Reagent: Competing Epoxidation
and Oxidative Breakdown Pathways and Novel Products of 5,6-Epoxyretinoic
Acid Transformation. Chem. Res. Toxicol..

[61] Margetić D. (2023). A Synopsis
of Ball Milling Organic Synthesis in the Last 25 Years. Curr. Org. Chem..

[62] Liu R., He X., Liu T., Wang X., Wang Q., Chen X., Lian Z. (2024). Organic Reactions
Enabled by Mechanical Force-Induced Single Electron
Transfer. Chem. - Eur. J..

[63] Viggiano S., Alfieri M. L., Panzella L., Crescenzi O., Napolitano A. (2024). Disclosing Novel Melanogenesis Pathways: Formation
of Unexpected Biphenyl-Type Dimers through Radical–Radical
Coupling by Solid-State Oxidation of the Melanin Biosynthetic Precursor
5,6-Dihydroxyindole. Bioorg. Chem..

[64] Basoccu F., De Luca L., Porcheddu A. (2024). Mechanochemistry
in Organic Synthesis:
An Italian Journey through Innovations. Eur.
J. Org. Chem..

[65] Naidu B. R., Sruthi T., Mitty R., Venkateswarlu K. (2023). Catalyst-Free
Mechanochemistry as a Versatile Tool in Synthetic Chemistry: A Review. Green Chem..

[66] Fantozzi N., Volle J. N., Porcheddu A., Virieux D., García F., Colacino E. (2023). Green Metrics in Mechanochemistry. Chem. Soc. Rev..

[67] Arfelis S., Martín-Perales A. I., Nguyen R., Pérez A., Cherubin I., Len C., Malpartida I., Bala A., Fullana-i-Palmer P. (2024). Linking Mechanochemistry with the
Green Chemistry Principles: Review Article. Heliyon.

[68] Kishimoto T., Takahashi N., Hamada M., Nakajima N. (2015). Biomimetic Oxidative
Coupling of Sinapyl Acetate by Silver Oxide: Preferential Formation
of β-O-4 Type Structures. J. Agric. Food
Chem..

[69] Tanahashi M., Takeuchi H., Higuchi T. (1976). Dehydrogenative Polymerization of
3,5-Disubstituted p-Coumaryl Alcohols. Wood
Res..

