# Supplementary material for: Tailoring the Oxidative Chemistry of Hydroxycinnamyl Alcohols: New and Green Access Routes to Lignans and Lignin Functional Derivatives
Source: J Agric Food Chem. 2026 Sep 15;74(37):29376–87. doi: 10.1021/acs.jafc.6c09238 (PMC13614303; doi:10.1021/acs.jafc.6c09238)
Supplement: Supplementary file 1 [file jf6c09238_si_001.pdf]

Supporting Information

Tailoring the Oxidative Chemistry of Hydroxycinnamyl Alcohols: New and Green Access Routes to Lignans and Lignin Functional Derivatives

Carmen Ruoppolo, Maria Laura Alfieri, Alessandra Napolitano and Lucia Panzella*

Department of Chemical Sciences, University of Naples Federico II, Via Cintia 4, I-80126, Naples, Italy

*Email: marialaura.alfieri@unina.it

Experimental Section

Materials and methods

Ferulic acid, *p*-coumaric acid, sinapic acid, ethyl chloroformate, anhydrous tetrahydrofuran (THF), triethylamine (TEA), sodium borohydride ($NaBH_4$), ammonium hydroxide (30% v/v), horseradish peroxidase (HRP), hydrogen peroxide (H_2O_2) (30% v/v), potassium ferricyanide ($K_3Fe(CN)_6$), laccase from *Trametes versicolor*, cerium(IV) ammonium nitrate (CAN), 2,2-diphenyl-1-picrylhydrazyl (DPPH), 2,2'-azobisisobutyronitrile (AIBN), manganese dioxide (MnO_2), silver(I) oxide (Ag_2O), sodium bicarbonate, iron chloride ($FeCl_3$), anhydrous sodium sulfate, lysine, glutathione (GSH), hexamethylenediamine (HMDA) and Nα-acetyl-L-lysine methyl ester hydrochloride were purchased from Sigma Aldrich.

UV-Vis spectra were recorded on a Jasco V-730 spectrophotometer.

LC-UV-MS analysis were run in negative ion mode using an Agilent 1260/6230DA ESI-TOF instrument under the following conditions: 35 psig nebulizer pressure; drying gas (nitrogen) fluxed at 5 L/min at a temperature of 325 °C; capillary voltage 3500 V; fragmenter voltage 175 V. An Eclipse Plus C18 column (150 × 4.6 mm, 5 µm) at a flow rate of 0.4 mL/min using binary gradient elution conditions as follows was used: 0.1% formic acid (solvent A)/methanol (solvent B) at 30% B in the first 10 min and from 30 % to 80%, from 10 to 35 min. UV detection wavelength was set at 254 nm.

NMR spectra were recorded in methanol-d_4 or acetone-d_6 at 400 MHz on a Bruker spectrometer. Chemical shifts are given in ppm.

Analytical and preparative TLC were carried out on silica gel plates (0.25 and 0.50 mm, respectively) from Merck using dichloromethane/methanol 98:2 v/v (eluent I) or chloroform/methanol/acetic acid 9.5:0.5:0.01 v/v/v (eluent II) as eluents.

Synthesis of hydroxycinnamyl alcohols

To 5 mmol of hydroxycinnamic acid (ferulic, sinapic or *p*-coumaric acid) (0.97-1.12 g) dissolved in anhydrous THF (50 mL) (final concentration 0.1 M), TEA was added (10 mmol, 1.39 mL) and the mixture was kept under stirring at room temperature. After 5 min, the mixture was cooled to 0 °C and ethyl chloroformate (10 mmol, 956 μL) was added dropwise over 10 min. Stirring was continued at room temperature for additional 30 min, after that the mixture was filtered to remove the insoluble material that was washed once with THF. To the combined filtrate and washing, kept in an open flask at 0 °C under stirring, an aqueous solution (7 mL) of $NaBH_4$ (20 mmol, 760 mg) was added dropwise. Stirring was prolonged for 20-30 min until no more gas evolved from the reaction mixture and after THF removal at a rotary evaporator, the mixture was extracted with ethyl acetate (3 × 50 mL). The combined organic layers were washed with water until neutral and dried on anhydrous sodium sulfate to get, after solvent removal, the protected hydroxycinnamyl alcohol in pure form, as assessed by TLC analysis (eluent I) (90-95 % w/w yield).

The protected hydroxycinnamyl alcohol (1 mmol) was then dissolved in 10 mL of methanol and treated dropwise with 90 mmol (4.5 mL) of a 30% v/v aqueous ammonia solution. After approximately 6 h, complete consumption of the starting material was confirmed by TLC (eluent II). The reaction mixture was cooled in an ice bath, acidified to pH 4-5 with 2 M HCl, and extracted with ethyl acetate (3 × 30 mL), and the combined organic layers were washed with water until neutral and dried with anhydrous sodium sulfate. After solvent removal, the hydroxycinnamyl alcohol (coniferyl alcohol (CA), sinapyl alcohol (SA), and *p*-coumaryl alcohol (PCA)) was obtained in pure form (NMR and LC-UV-MS evidence) in 55-70% w/w yield.

All solvents were recovered and reused, when possible, to minimize waste generation.

General protocol for hydroxycinnamyl alcohol oxidation in aqueous solution

To a solution of CA, SA or PCA (0.027 mmol, 4.0-5.7 mg, pre-dissolved in 1 mL of methanol) in 50 mM sodium phosphate buffer (pH 6.8, final concentration 1 mM) HRP (1 U/mL) and 0.2 molar eqs of H_2O_2 were added. The reaction mixture was taken under stirring at room temperature and periodically analyzed by LC-UV-MS.

In other experiments the oxidation reaction was performed:

- in the presence of HRP (1 U/mL) and 0.4-0.8 molar eqs of H_2O_2;
- in the presence of $K_3Fe(CN)_6$ (1-5 molar eqs);
- in the presence of CAN (1 molar eq) in sodium phosphate (25 mM)/sodium citrate (25 mM) buffer (pH 3.0);
- in the presence of laccase (0.5 U/mL) in sodium phosphate (25 mM)/sodium citrate (25 mM) buffer (pH 3.0);
- in the presence of $K_3Fe(CN)_6$ (1 molar eq) and HMDA (5 molar eqs) in 50 mM sodium phosphate buffer (pH 9.0);
- in the presence of $K_3Fe(CN)_6$ (1 molar eq) and lysine (10 molar eqs) in 50 mM sodium phosphate buffer (pH 9.0);
- in the presence of $K_3Fe(CN)_6$ (1 molar eq) and Nα-acetyl-L-lysine methyl ester (10 molar eqs) in 50 mM sodium phosphate buffer (pH 9.0);
- in the presence of HRP (1 U/mL), H_2O_2 (0.2 or 0.4 molar eqs) and GSH (5 molar eqs) in 50 mM sodium phosphate buffer (pH 6.8).

General protocol for the hydroxycinnamyl alcohol oxidation in organic solvents

To a solution of CA, SA or PCA (0.027 mmol, 4.0-5.7 mg) in 27 mL of methanol (final concentration 1 mM) DPPH (1 molar eq, 9-13 mg) was added. The reaction mixture was kept under stirring at room temperature and periodically analyzed by LC-UV-MS.

In other experiments the oxidation reaction was performed:

- in the presence of DPPH (1 molar eq) in acetonitrile (27 mL) (final substrate concentration 1 mM);
- in the presence of AIBN (1 molar eq) in acetonitrile (27 mL) (final substrate concentration 1 mM) at 60 °C;
- in the presence of MnO_2 (1-4 molar eqs) in dichloromethane (2.7-27 mL) (final concentration 1-10 mM);
- in the presence of Ag_2O (1-8 molar eqs) in 1:2 v/v acetone/2 M sodium citrate buffer (pH=2.5) (final concentration 185 mM);
- in the presence of $FeCl_3$ (0.8 molar eqs) in a 5:2 v/v dioxane/water or acetone/water mixture (final substrate concentration 1 or 62 mM);
- in the presence of $K_3Fe(CN)_6$ (0.8-4 molar eqs) in a 5:2 v/v dioxane/water or acetone/water mixture (final substrate concentration 62 mM).

Solid-state oxidation of hydroxycinnamyl alcohols

5 mg of CA, SA or PCA were placed in a 5 mL grinding jar made of stainless steel with two 2 mm-diameter stainless steel grinding balls in the presence of $K_3Fe(CN)_6$ (1-5 molar eq). The mixture was milled for 30 min in a mini-mill Pulverisette 23 (Fritsch) operating at a frequency of 50 oscillation/s at room temperature, after that the powder was dissolved in methanol or acetone and analyzed by LC-UV-MS.

In other experiments the oxidation reaction was performed:

- in the presence of $K_3Fe(CN)_6$ (1 molar eq) and sodium bicarbonate (5 molar eqs);
- in the presence of CAN (1 molar eq);
- in the presence of DPPH (1 molar eq);
- in the presence of MnO_2 (1 molar eq);
- in the presence of Ag_2O (1-5 molar eqs).

A)

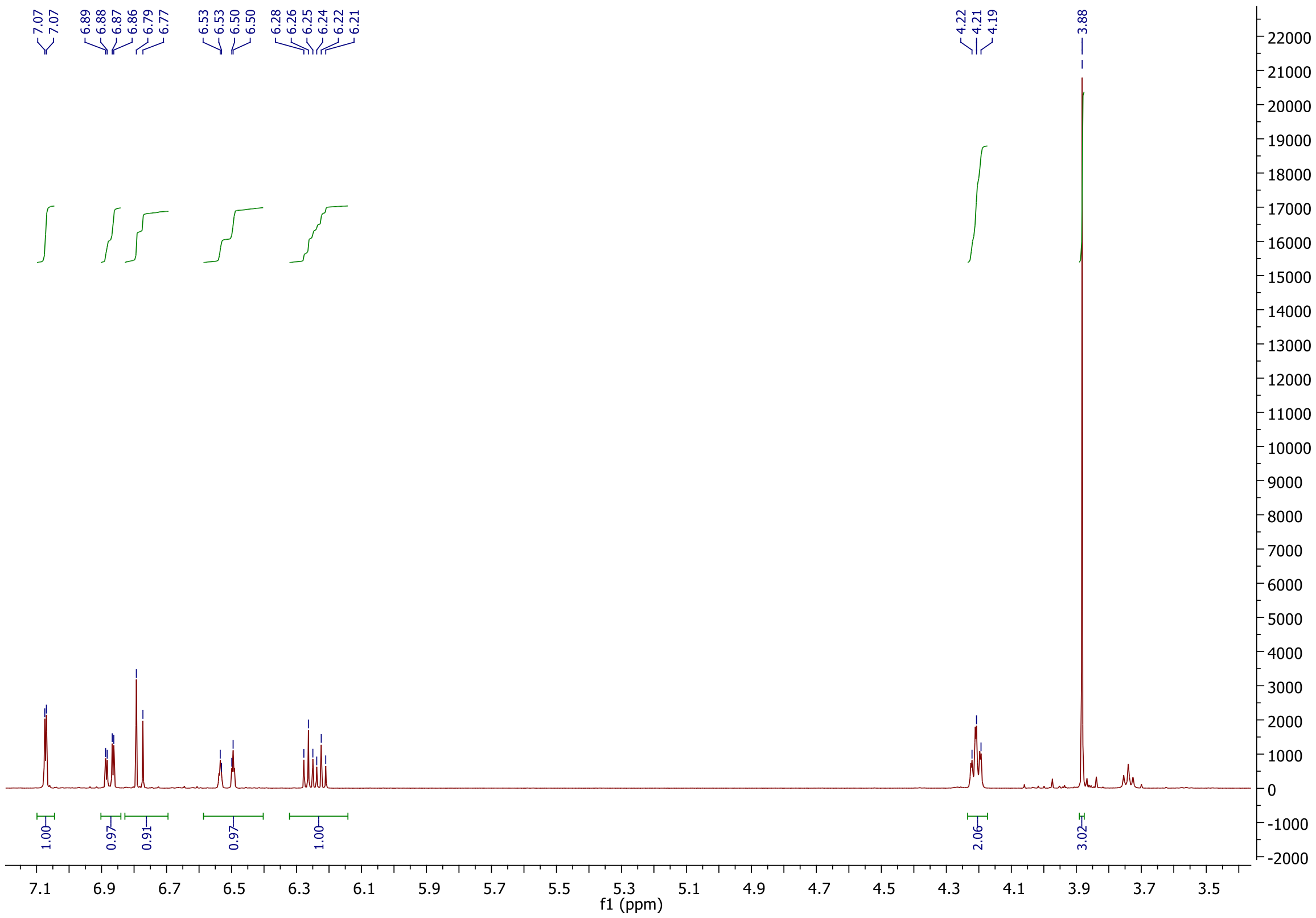

B)

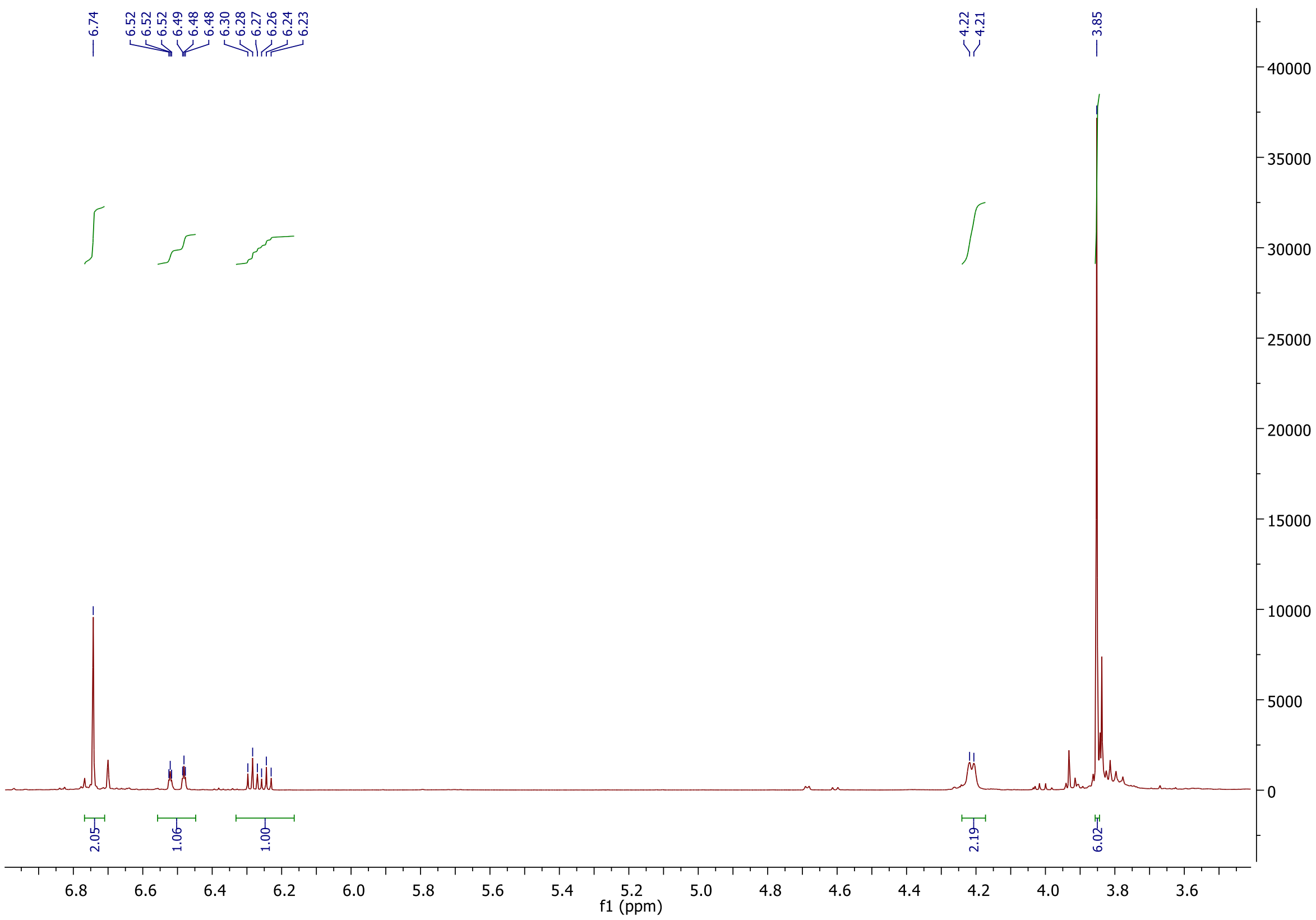

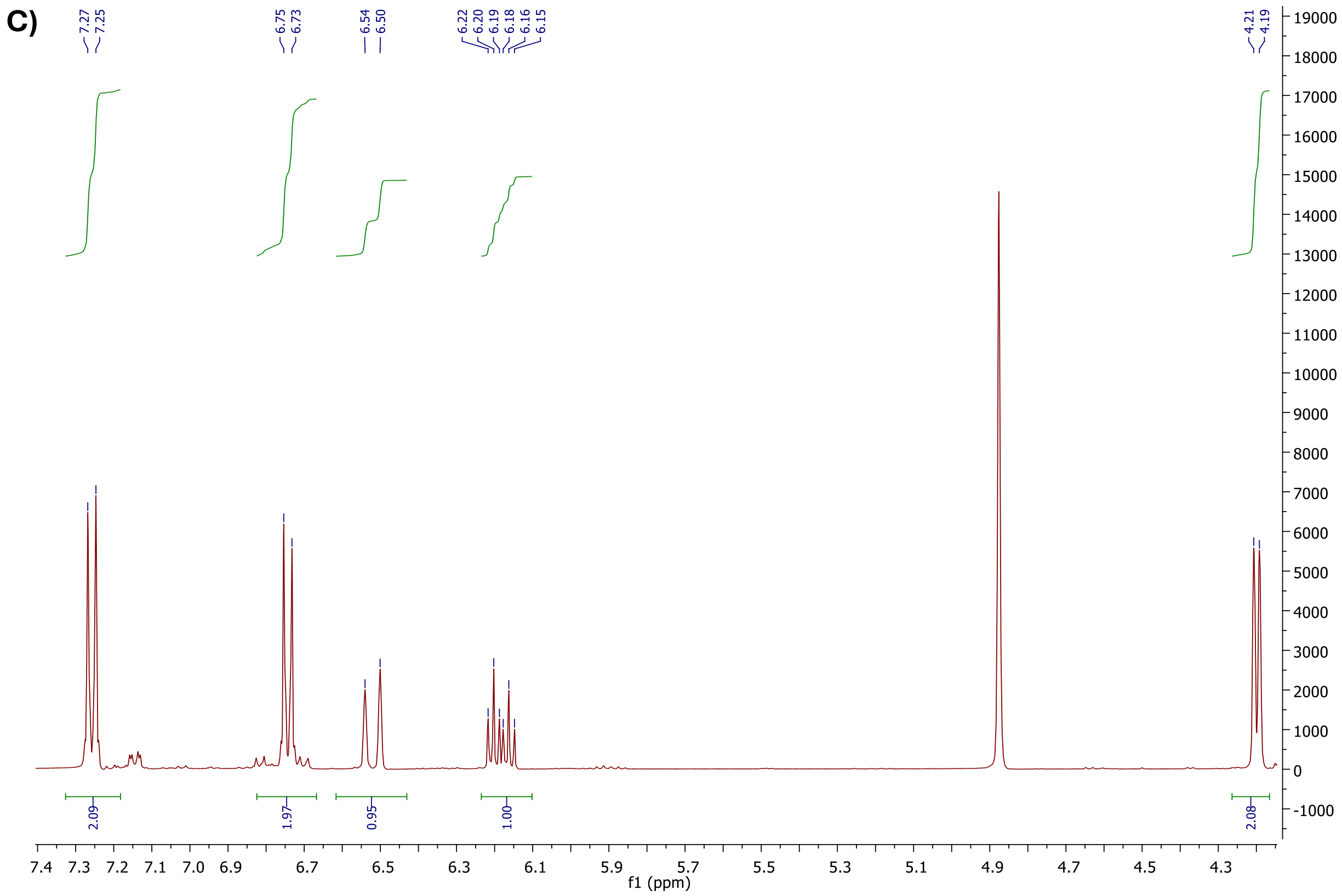

Figure S1. ^{1}H NMR spectra of coniferyl alcohol (CA) (A) in acetone-*d₆*, sinapyl alcohol (SA) (B) in acetone-*d₆* and *p*-coumaryl alcohol (PCA) (C) in methanol-*d₄*.

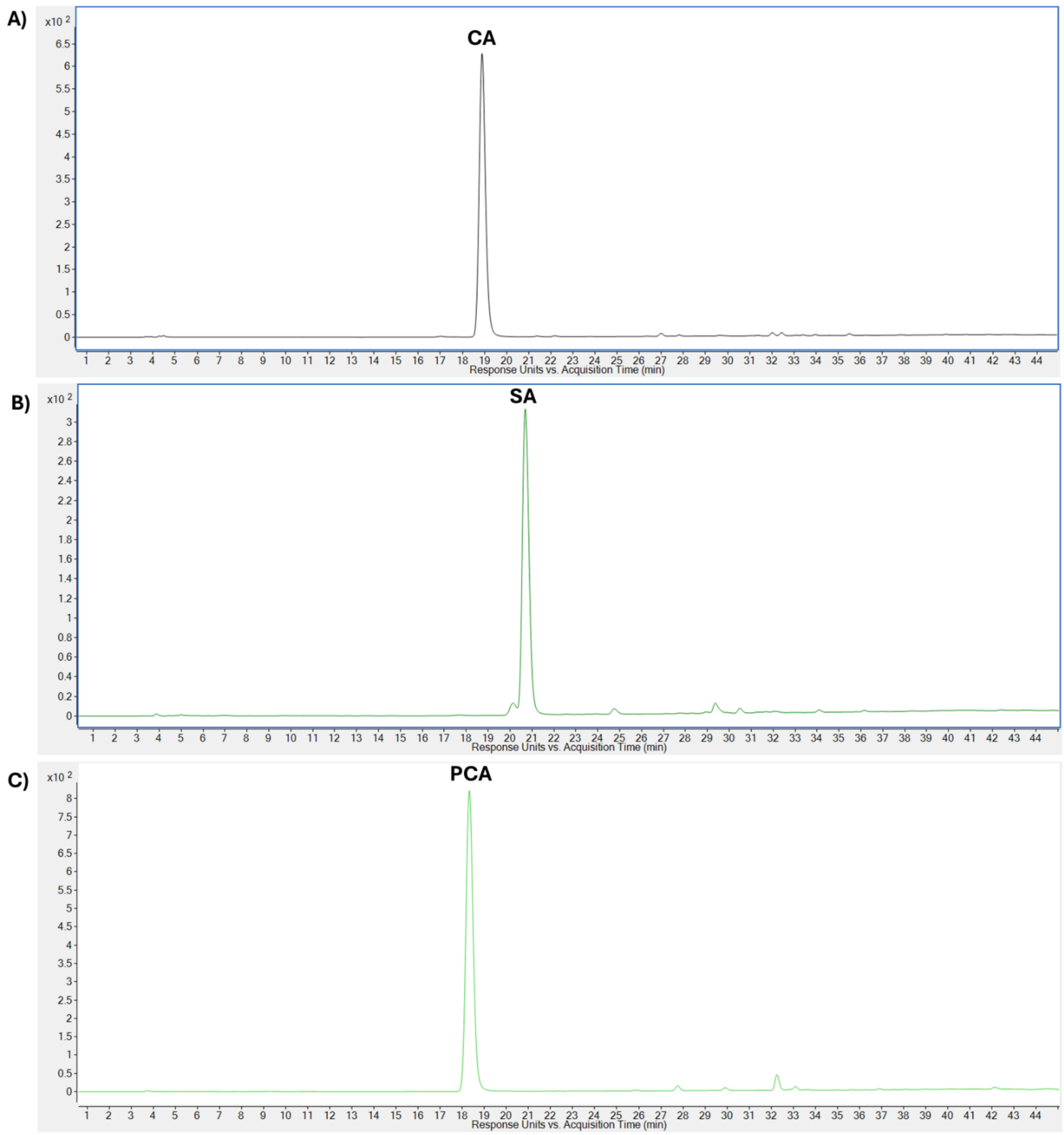

Figure S2. HPLC profiles of synthesized coniferyl alcohol (CA) (A), sinapyl alcohol (SA) (B) and *p*-coumaryl alcohol (PCA) (C). Analysis was performed under the following conditions: Eclipse Plus C18 column (150 × 4.6 mm, 5 µm). Eluent: 0.1% formic acid (solvent A)/methanol (solvent B): 30% B (0-10 min), from 30 to 80% B (10-35 min). Detection wavelength: 254 nm.

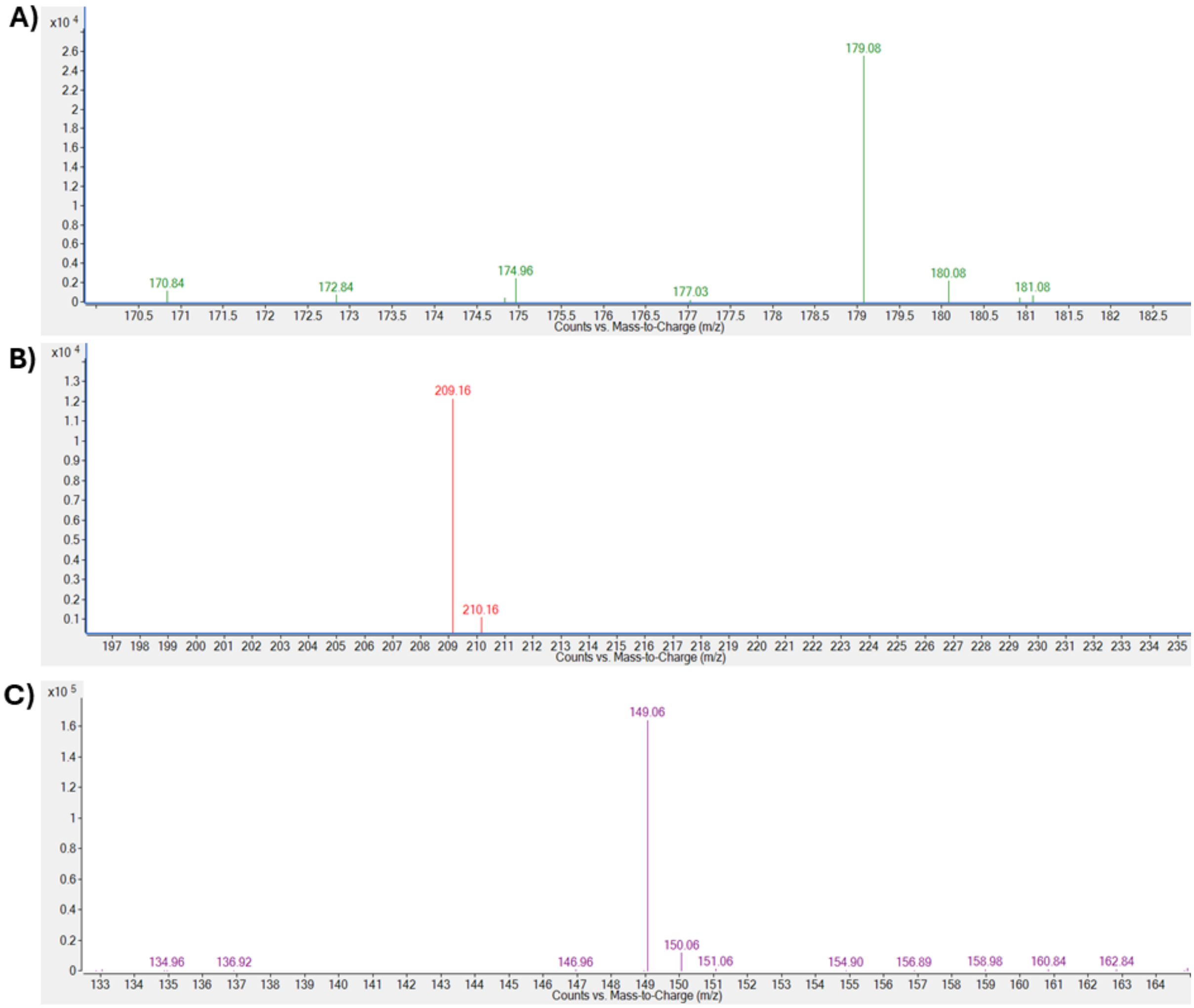

Figure S3. Mass spectra (negative ion mode) of synthesized coniferyl alcohol (CA) (A), sinapyl alcohol (SA) (B) and *p*-coumaryl alcohol (PCA) (C).

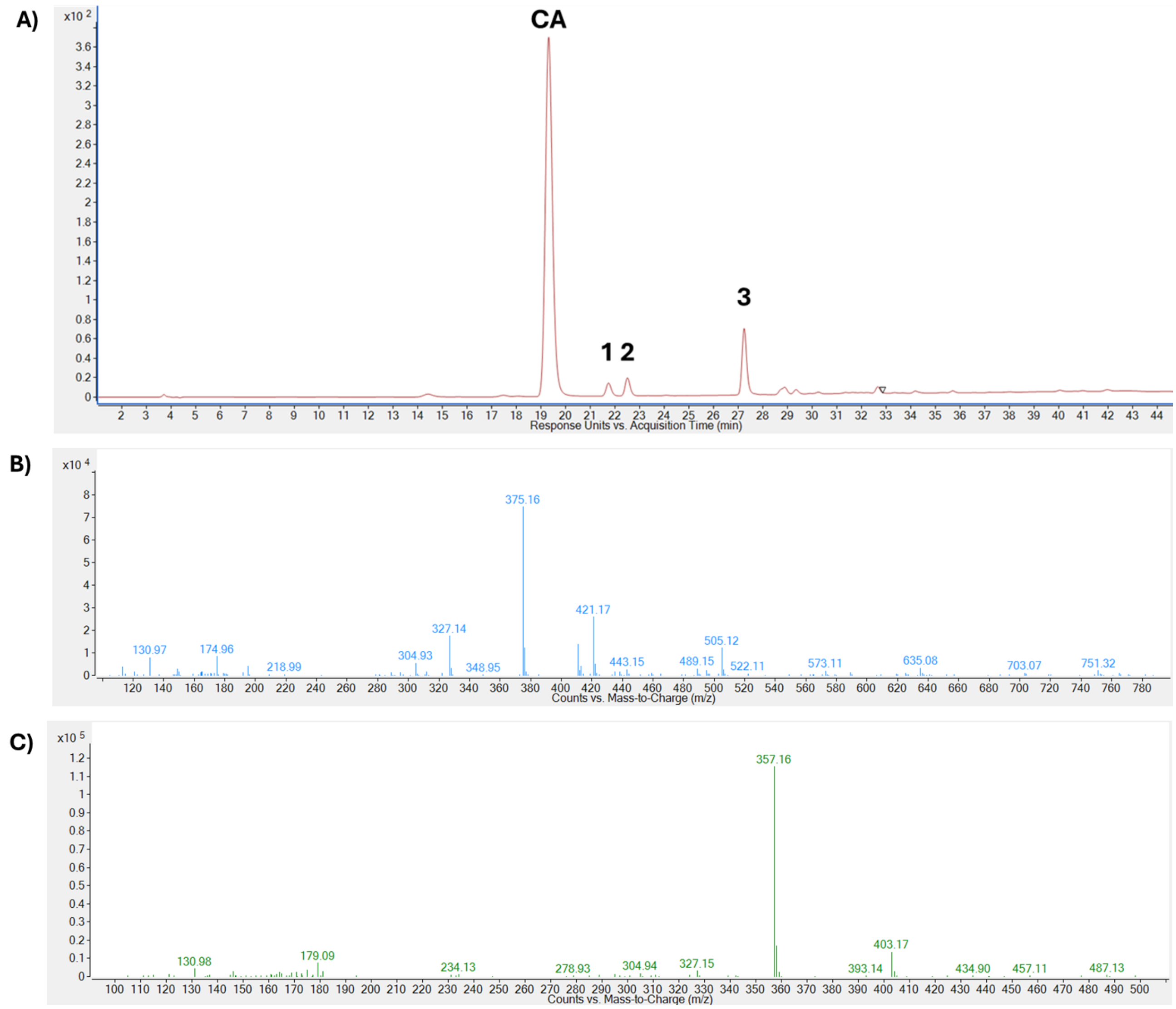

Figure S4. A) HPLC profile of the reaction mixture of coniferyl alcohol (CA) in the presence of HRP (1 U/mL) and H_2O_2 (0.4 molar eqs) after 1 h. Analysis was performed under the following conditions: Eclipse Plus C18 column (150 × 4.6 mm, 5 µm). Eluent: 0.1% formic acid (solvent A)/methanol (solvent B): 30% B (0-10 min), from 30 to 80% B (10-35 min). Detection wavelength: 254 nm. CA indicates residual coniferyl alcohol. Compounds **1** and **2** are the β-O-4-coupled dimers whereas compound **3** is the β-5-coupled dimer of CA as shown in Figure 4. Mass spectra (negative ion mode) of compounds **1** and **2** (B) and **3** (C).

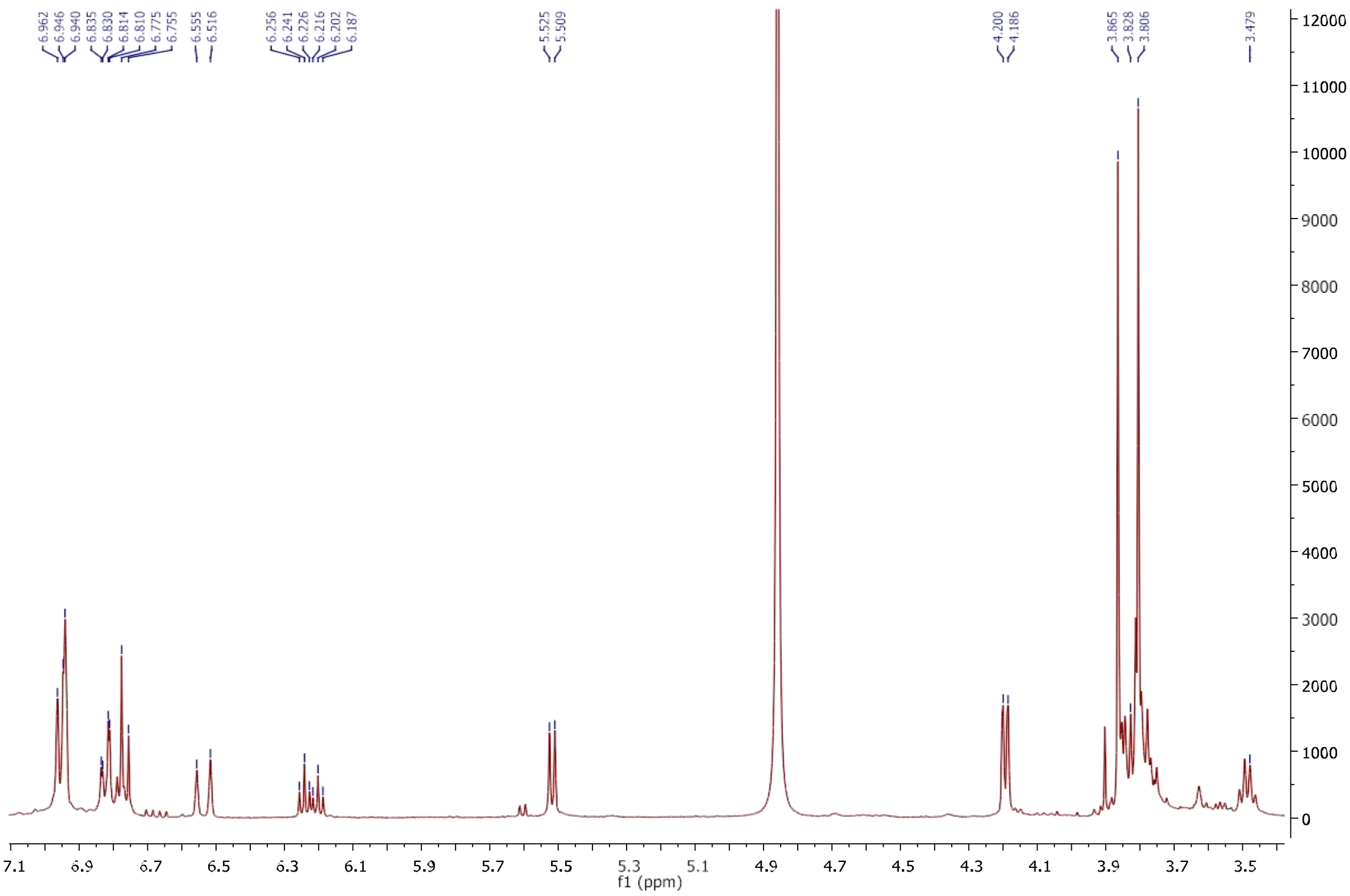

Figure S5. ^{1}H NMR spectrum of **3** in methanol-d_4.

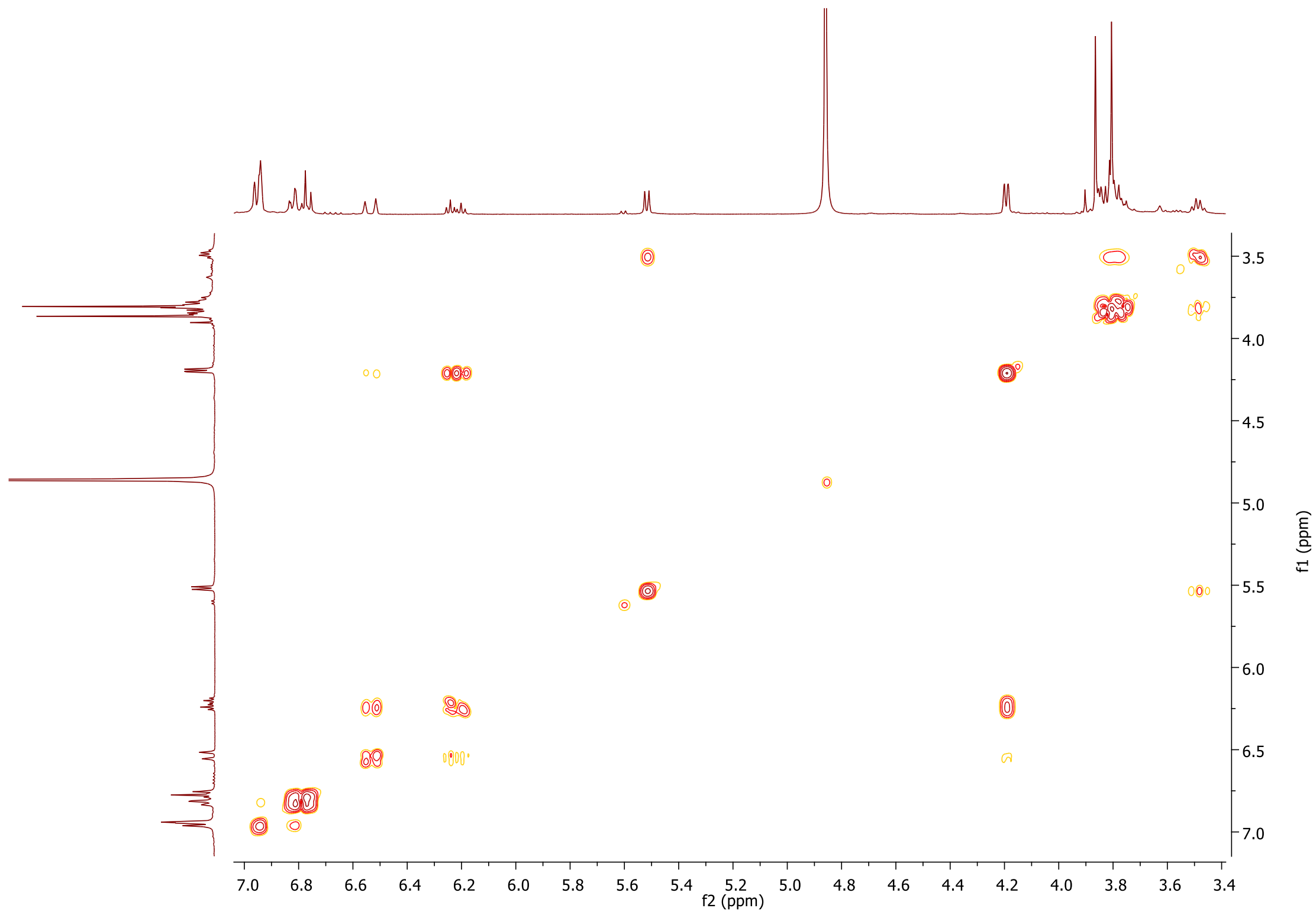

Figure S6. ^{1}H,^{1}H COSY spectrum of **3** in methanol-d_4.

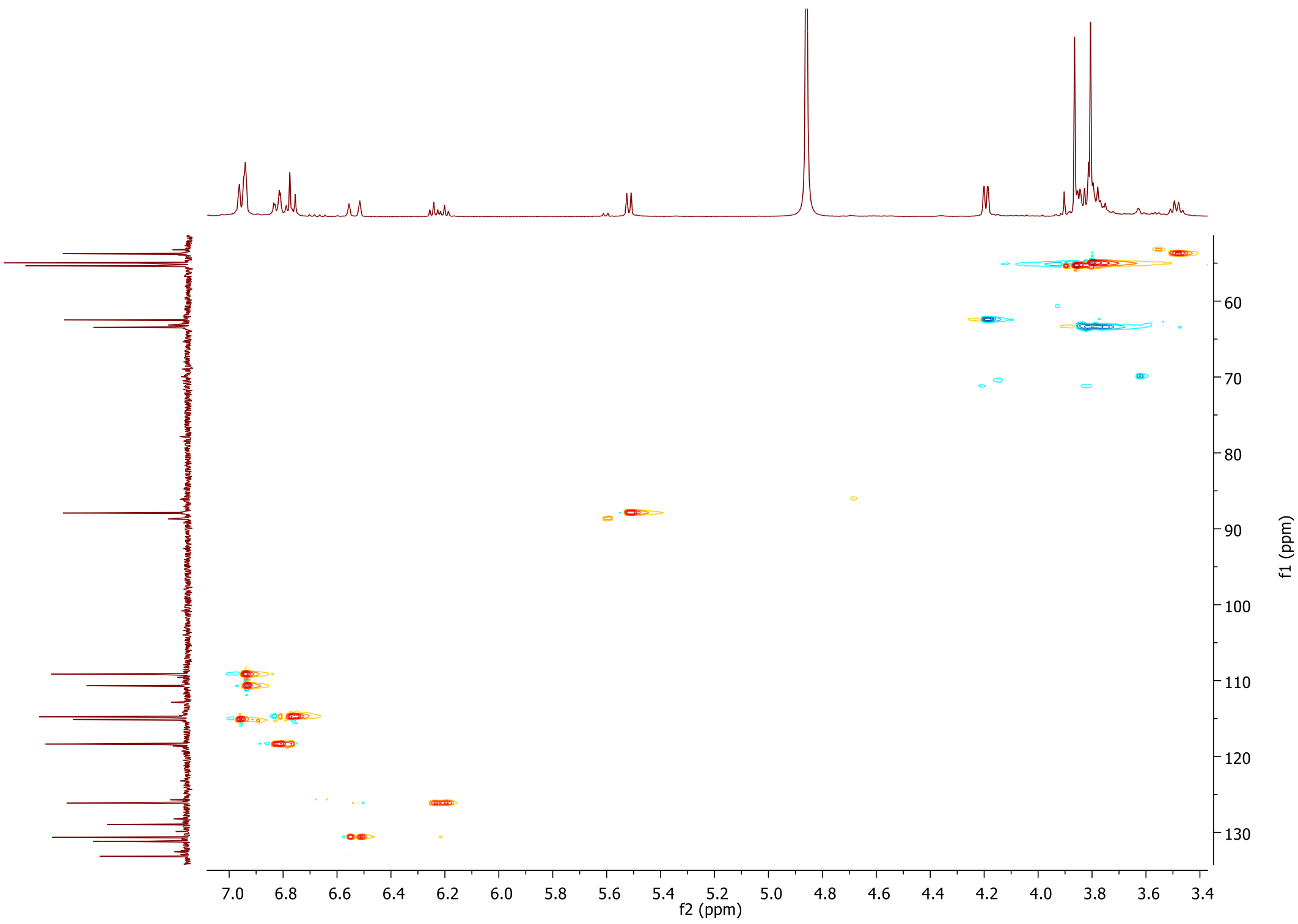

Figure S7. $^{1}H,^{13}C$ HSQC spectrum of **3** in methanol-d_4.

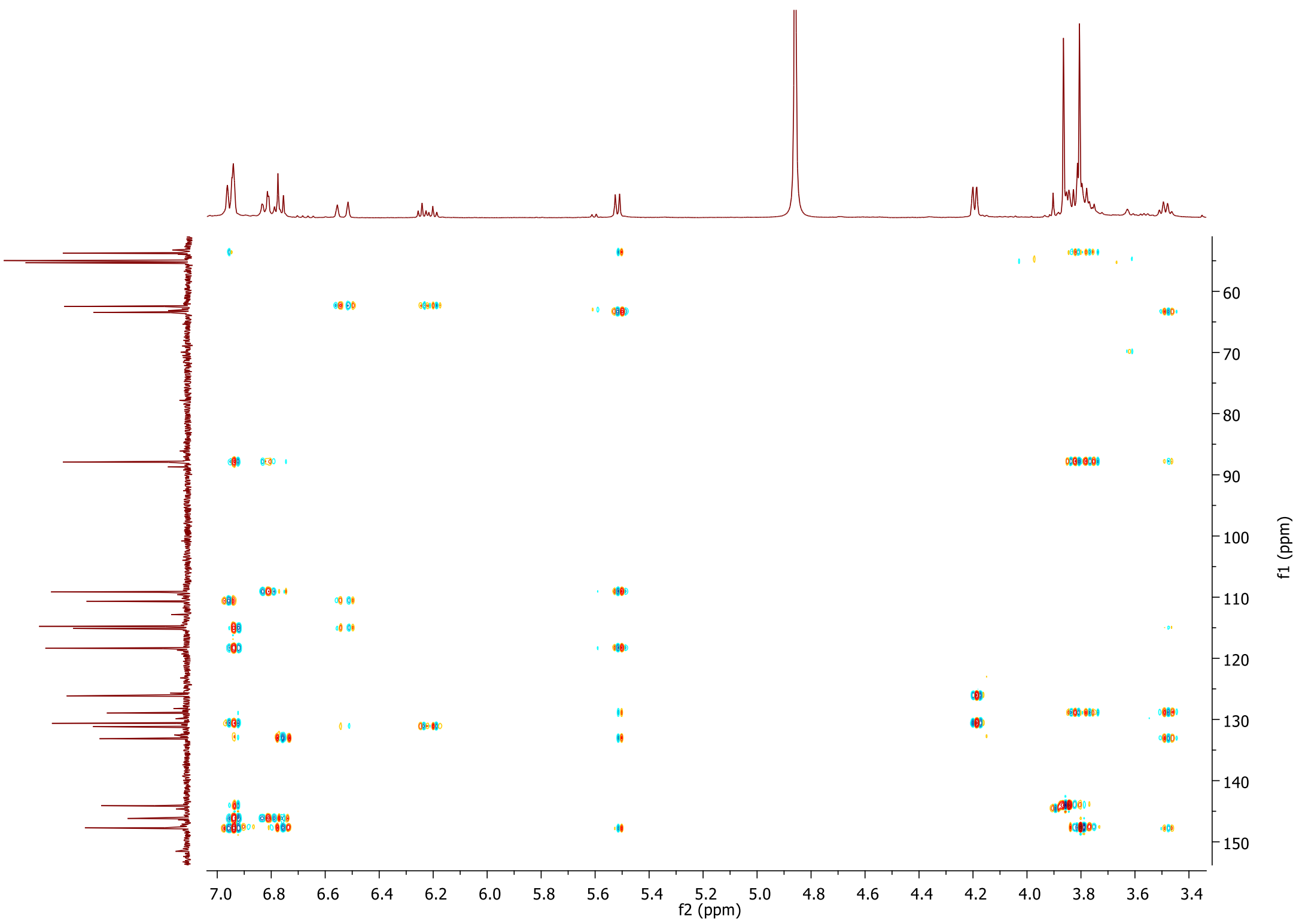

Figure S8. $^{1}H,^{13}C$ HMBC spectrum of **3** in methanol-d_4.

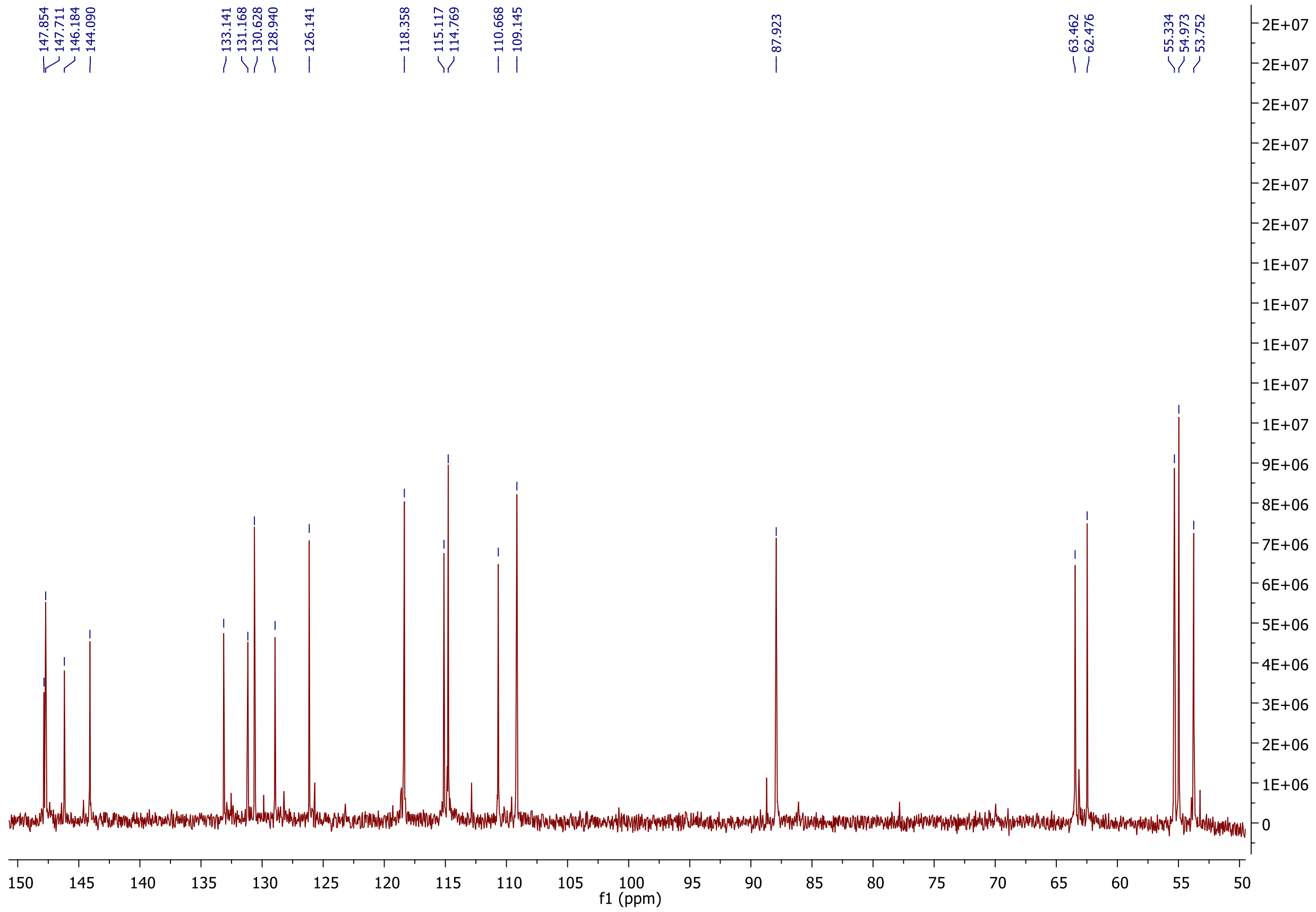

Figure S9. ^{13}C NMR spectrum of **3** in methanol-d_4.

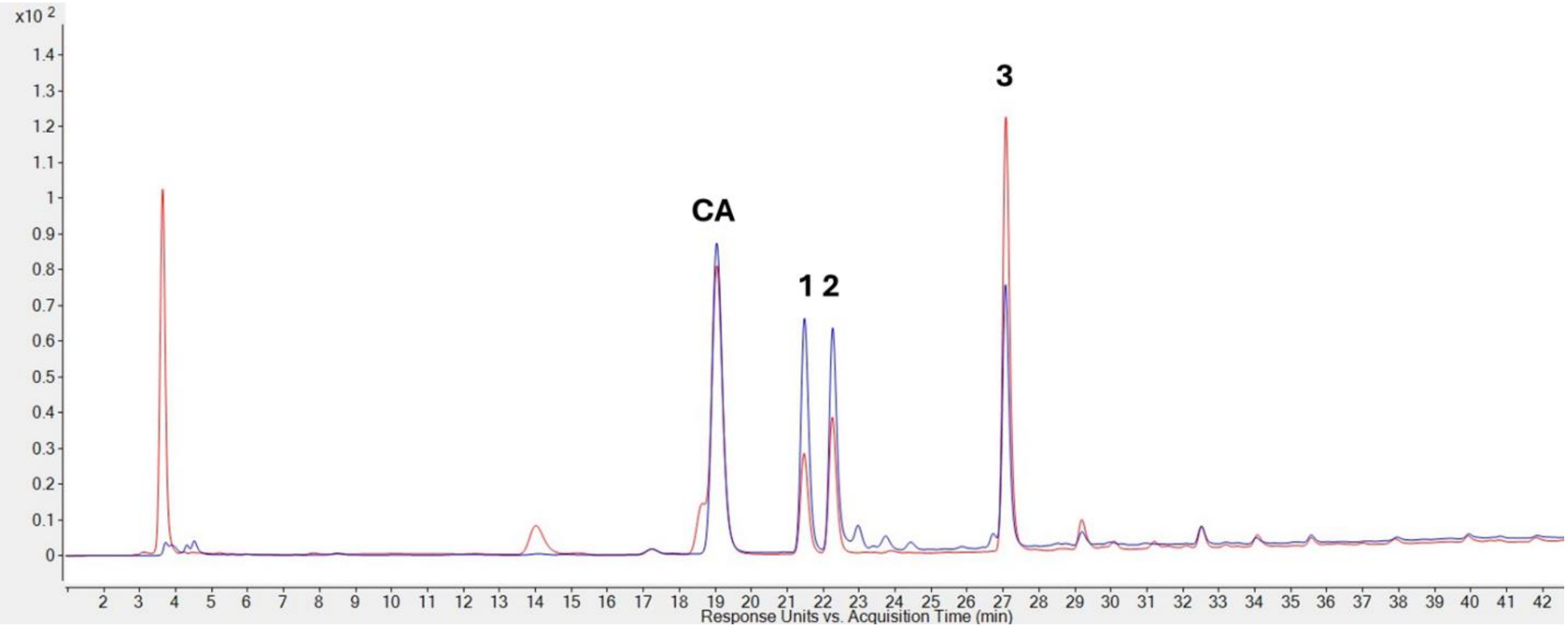

Figure S10. Superimposed HPLC profiles of the reaction mixtures of coniferyl alcohol (CA) in the presence of 2 molar equivalents of $K_3Fe(CN)_6$ at pH 6.8 (red line) or 0.5 U/mL of laccase at pH 3.0 (blue line) after 1 h. Analysis was performed under the following conditions: Eclipse Plus C18 column (150 × 4.6 mm, 5 µm). Eluent: 0.1% formic acid (solvent A)/methanol (solvent B): 30% B (0-10 min), from 30 to 80% B (10-35 min). Detection wavelength: 254 nm. CA indicates residual coniferyl alcohol. Compounds **1** and **2** are the β-O-4-coupled dimers whereas compound **3** is the β-5-coupled dimer of CA as shown in Figure 4.

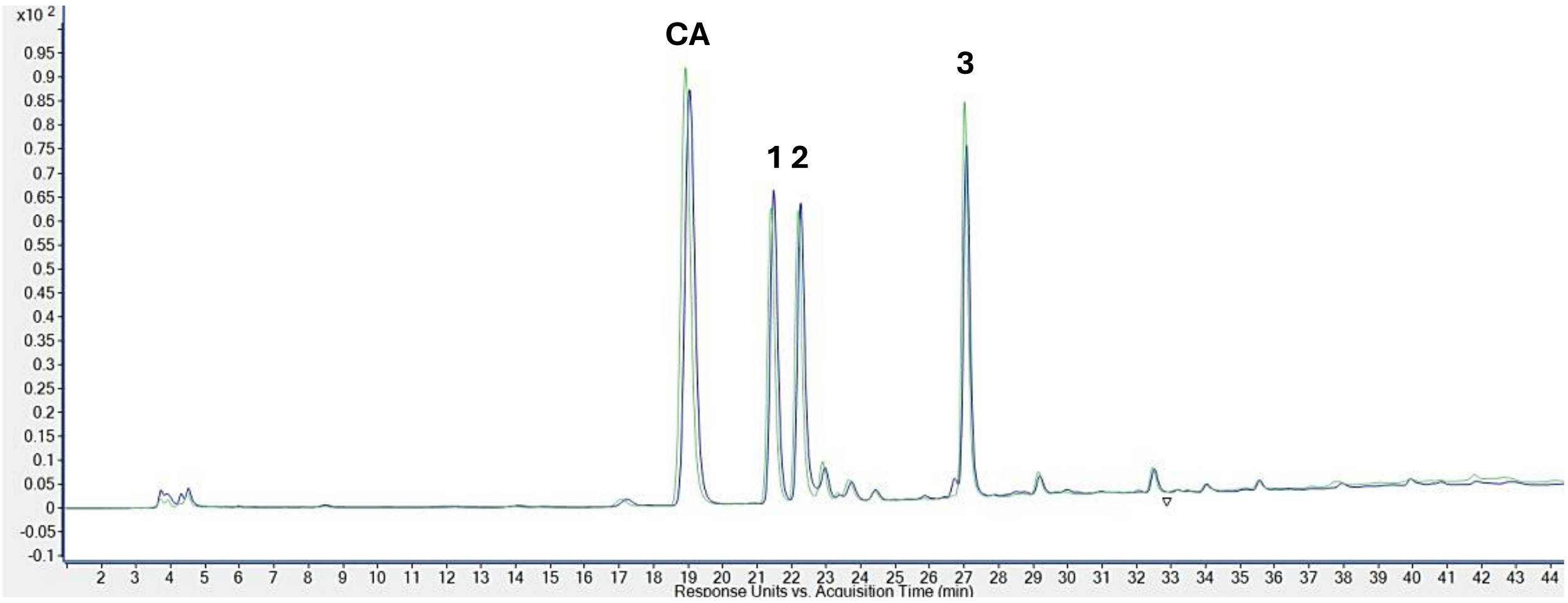

Figure S11. Superimposed HPLC profiles of the reaction mixtures of coniferyl alcohol (CA) in the presence of 1 molar equivalent of CAN (blue line) or 0.5 U/mL of laccase (green line) at pH 3.0 after 1 h. Analysis was performed under the following conditions: Eclipse Plus C18 column (150 × 4.6 mm, 5 µm). Eluent: 0.1% formic acid (solvent A)/methanol (solvent B): 30% B (0-10 min), from 30 to 80% B (10-35 min). Detection wavelength: 254 nm. CA indicates residual coniferyl alcohol. Compounds **1** and **2** are the β-O-4-coupled dimers whereas compound **3** is the β-5-coupled dimer of CA as shown in Figure 4.

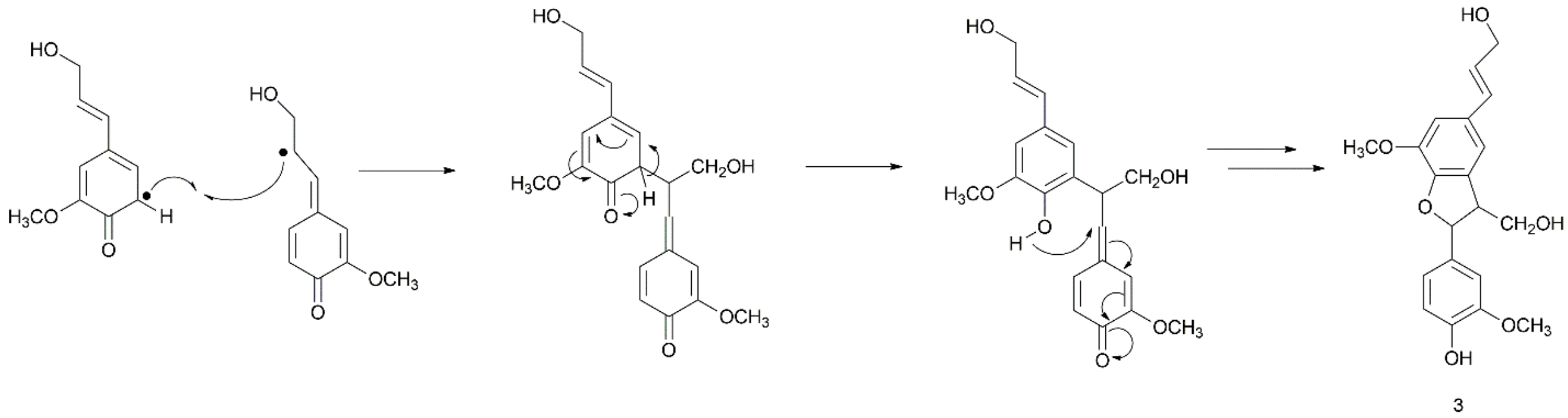

Figure S12. Proposed pathway for the formation of **3**.

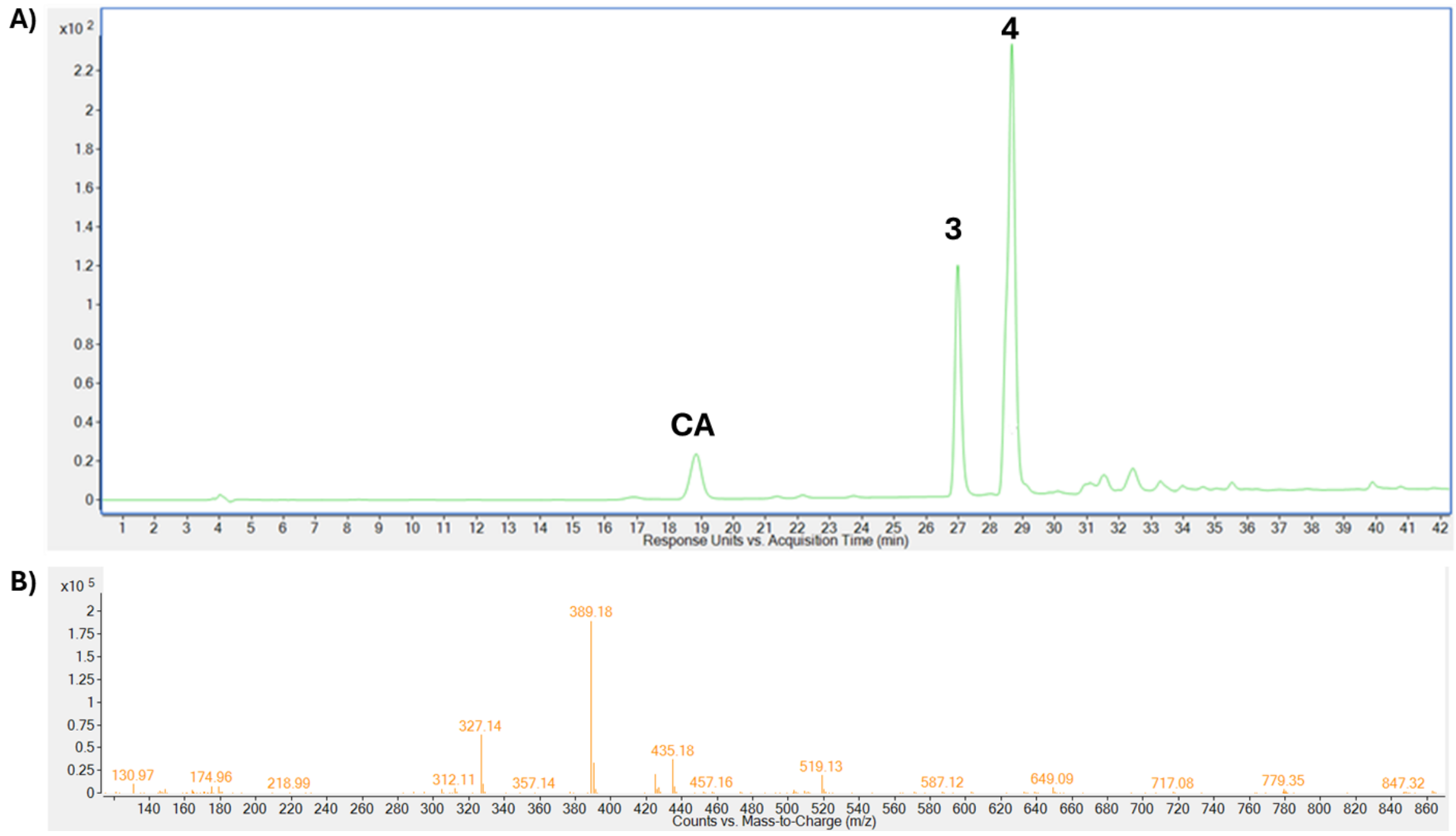

Figure S13. A) HPLC profile of the reaction mixture of coniferyl alcohol (CA) with DPPH in methanol after 1 h. Analysis was performed under the following conditions: Eclipse Plus C18 column (150 × 4.6 mm, 5 µm). Eluent: 0.1% formic acid (solvent A)/methanol (solvent B): 30% B (0-10 min), from 30 to 80% B (10-35 min). Detection wavelength: 254 nm. CA indicates residual coniferyl alcohol. Compound **3** is the β-5-coupled dimer whereas compound **4** is consistent with a CA dimer arising from addition of methanol instead of water. B) Mass spectrum (negative ion mode) of compound **4**.

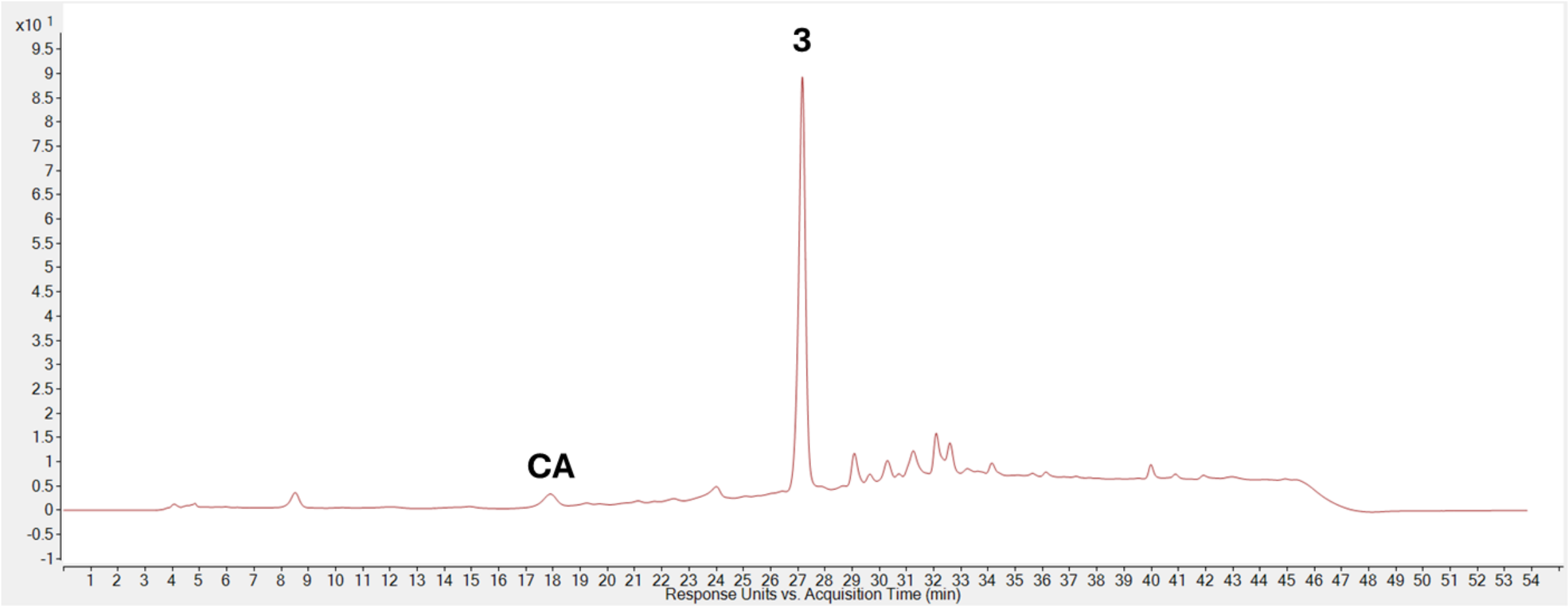

Figure S14. HPLC profile of the reaction mixtures of coniferyl alcohol (CA) with AIBN in acetonitrile after 24 h at 60 °C. Analysis was performed under the following conditions: Eclipse Plus C18 column (150 × 4.6 mm, 5 µm). Eluent: 0.1% formic acid (solvent A)/methanol (solvent B): 30% B (0-10 min), from 30 to 80% B (10-35 min). Detection wavelength: 254 nm. CA indicates residual coniferyl alcohol. Compound **3** is the β-5-coupled dimer as shown in Figure 4.

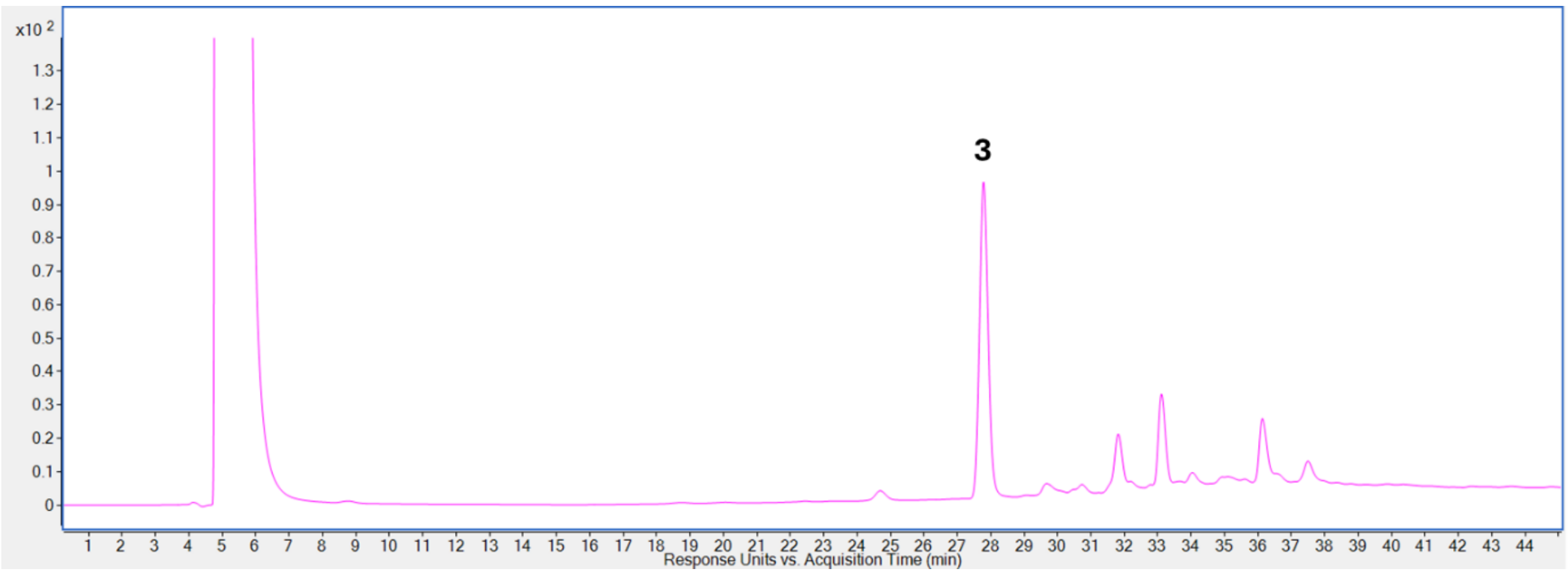

Figure S15. HPLC profile of the reaction mixtures of coniferyl alcohol (CA) with MnO_2 in dichloromethane after 96 h. Analysis was performed under the following conditions: Eclipse Plus C18 column (150 × 4.6 mm, 5 µm). Eluent: 0.1% formic acid (solvent A)/methanol (solvent B): 30% B (0-10 min), from 30 to 80% B (10-35 min). Detection wavelength: 254 nm. Compound **3** is the β-5-coupled dimer as shown in Figure 4.

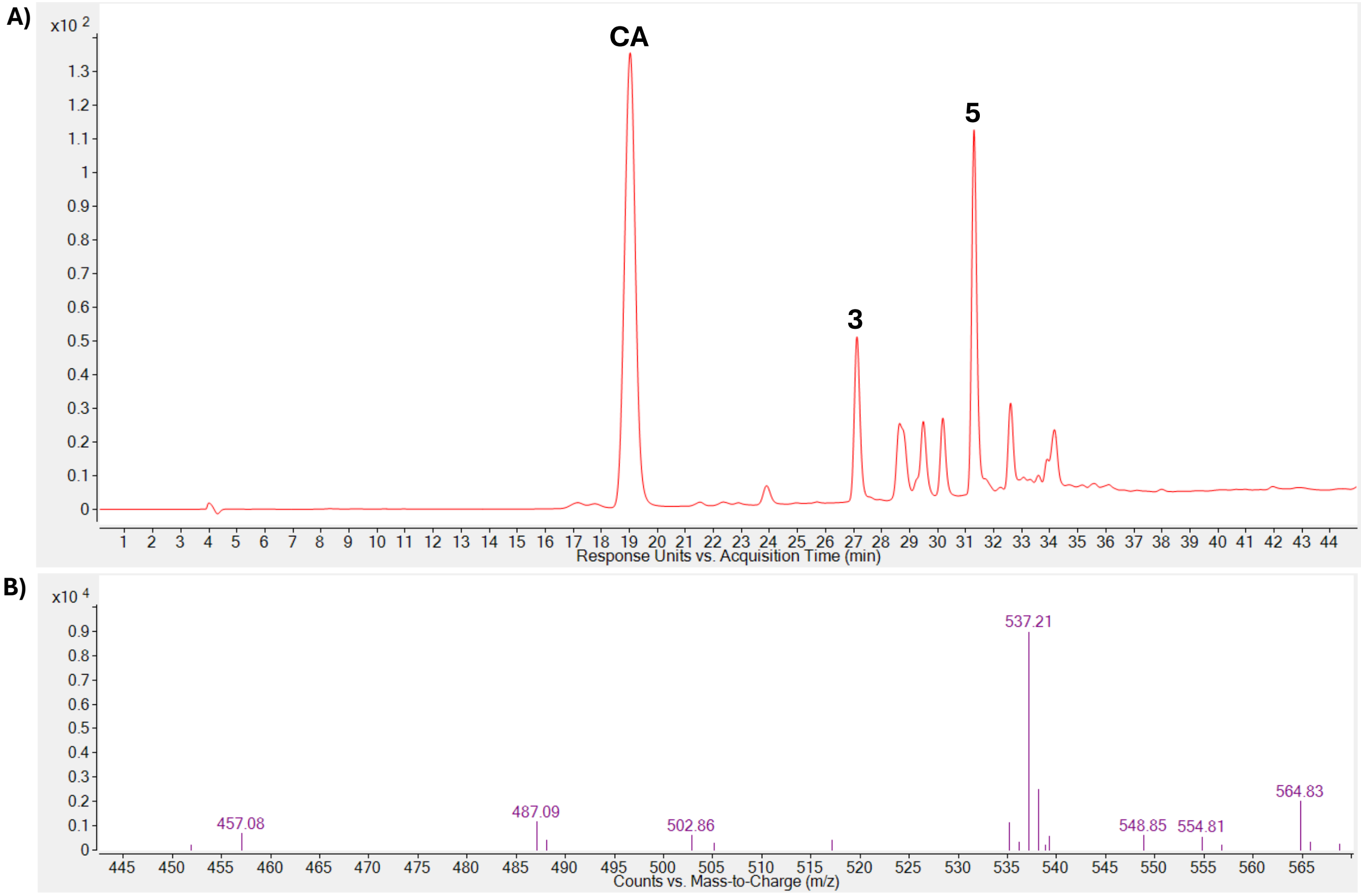

Figure S16. A) HPLC profile of the reaction mixture obtained by solid-state oxidation of coniferyl alcohol (CA) for 30 min in the presence of $K_3Fe(CN)_6$ (1 molar eq.) and sodium bicarbonate (5 molar eqs). Analysis was performed under the following conditions: Eclipse Plus C18 column (150 × 4.6 mm, 5 µm). Eluent: 0.1% formic acid (solvent A)/methanol (solvent B): 30% B (0-10 min), from 30 to 80% B (10-35 min). Detection wavelength: 254 nm. CA indicates residual coniferyl alcohol. Compound **3** is the β-5-coupled dimer whereas compound **5** is the trimeric derivative of CA as shown in Figure 4 and 7, respectively. B) Mass spectrum of compound **5**.

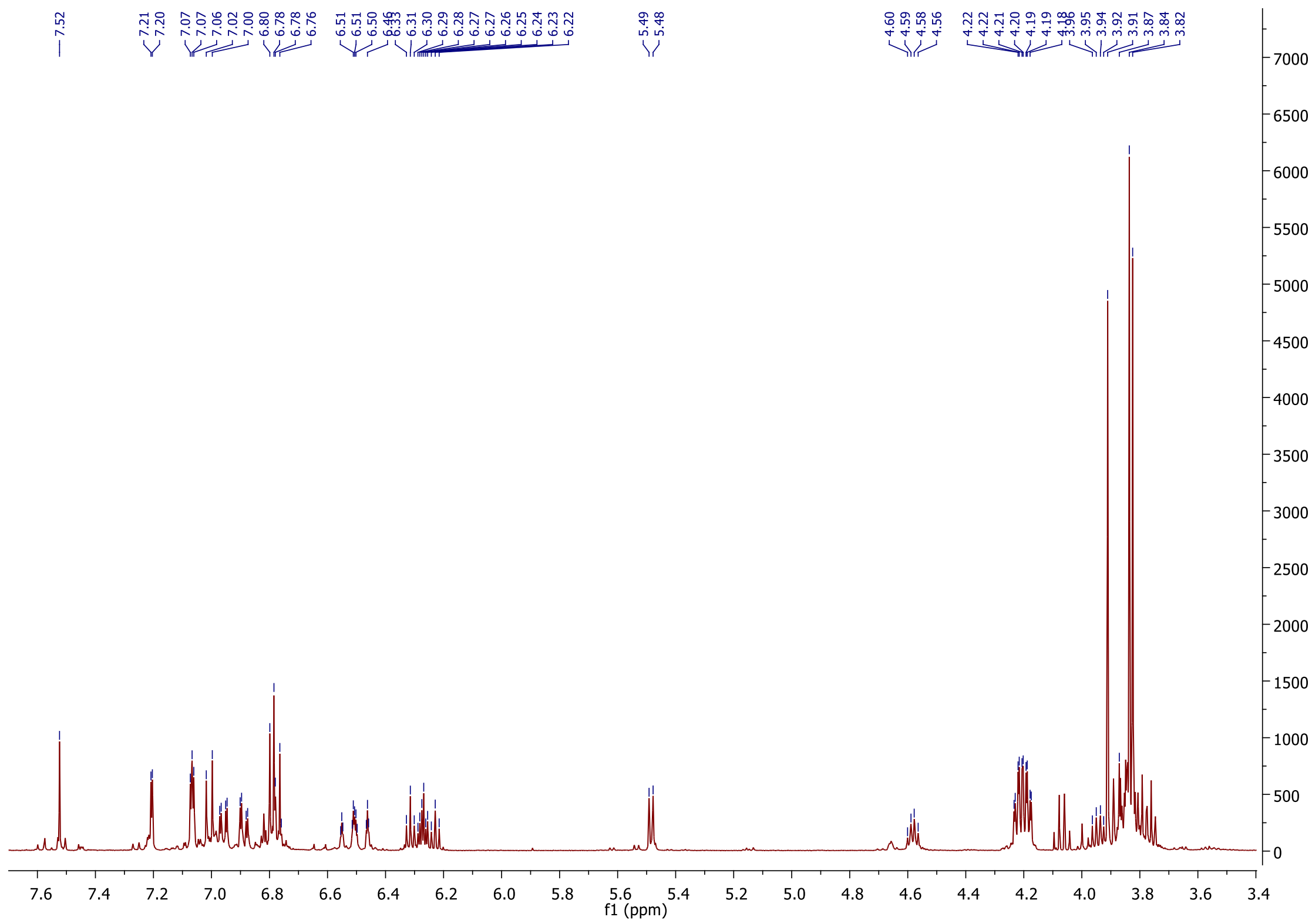

Figure S17. ^{1}H NMR spectrum of **5** in acetone-d_6.

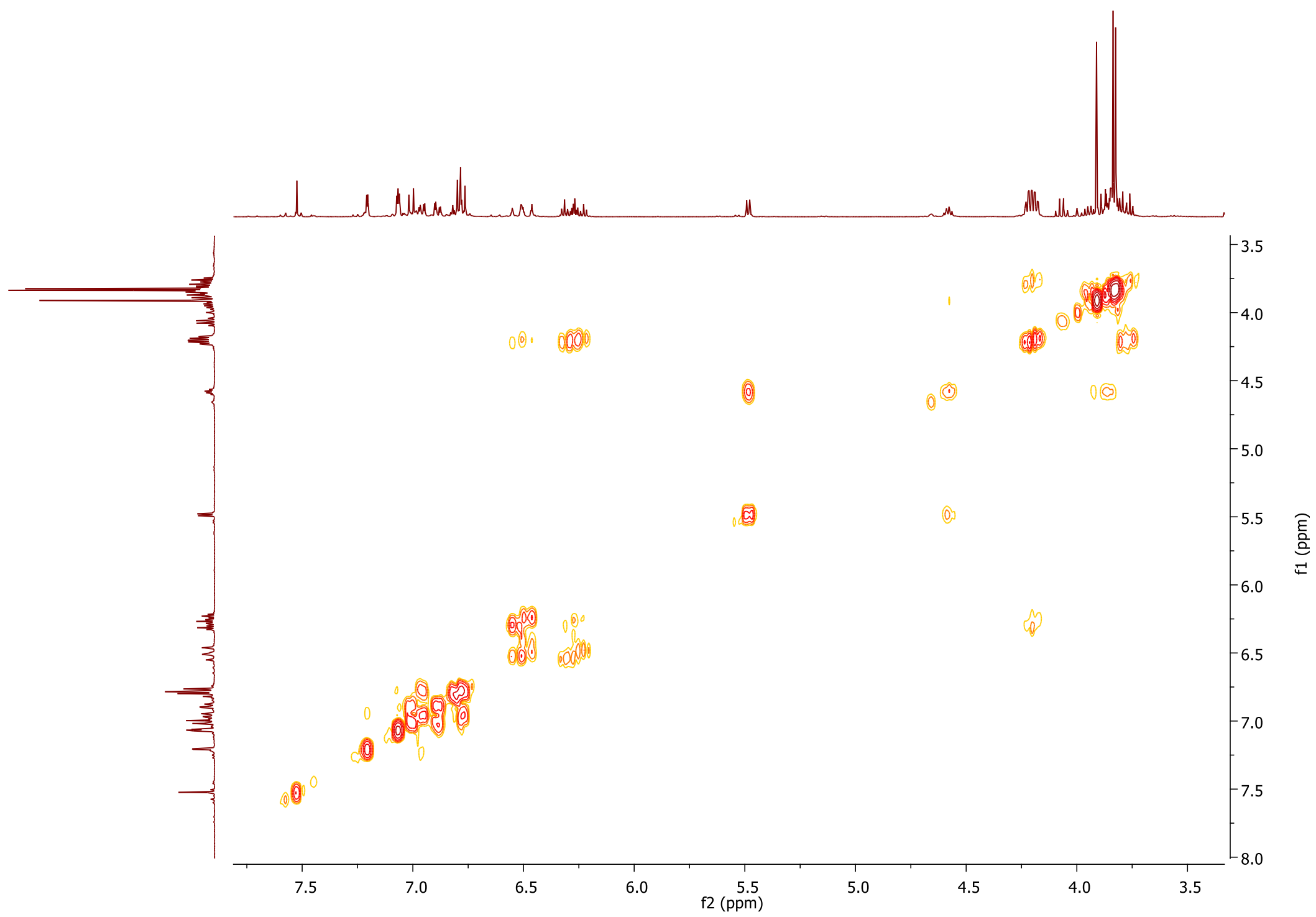

Figure S18. ^{1}H,^{1}H COSY spectrum of **5** in acetone-d_6.

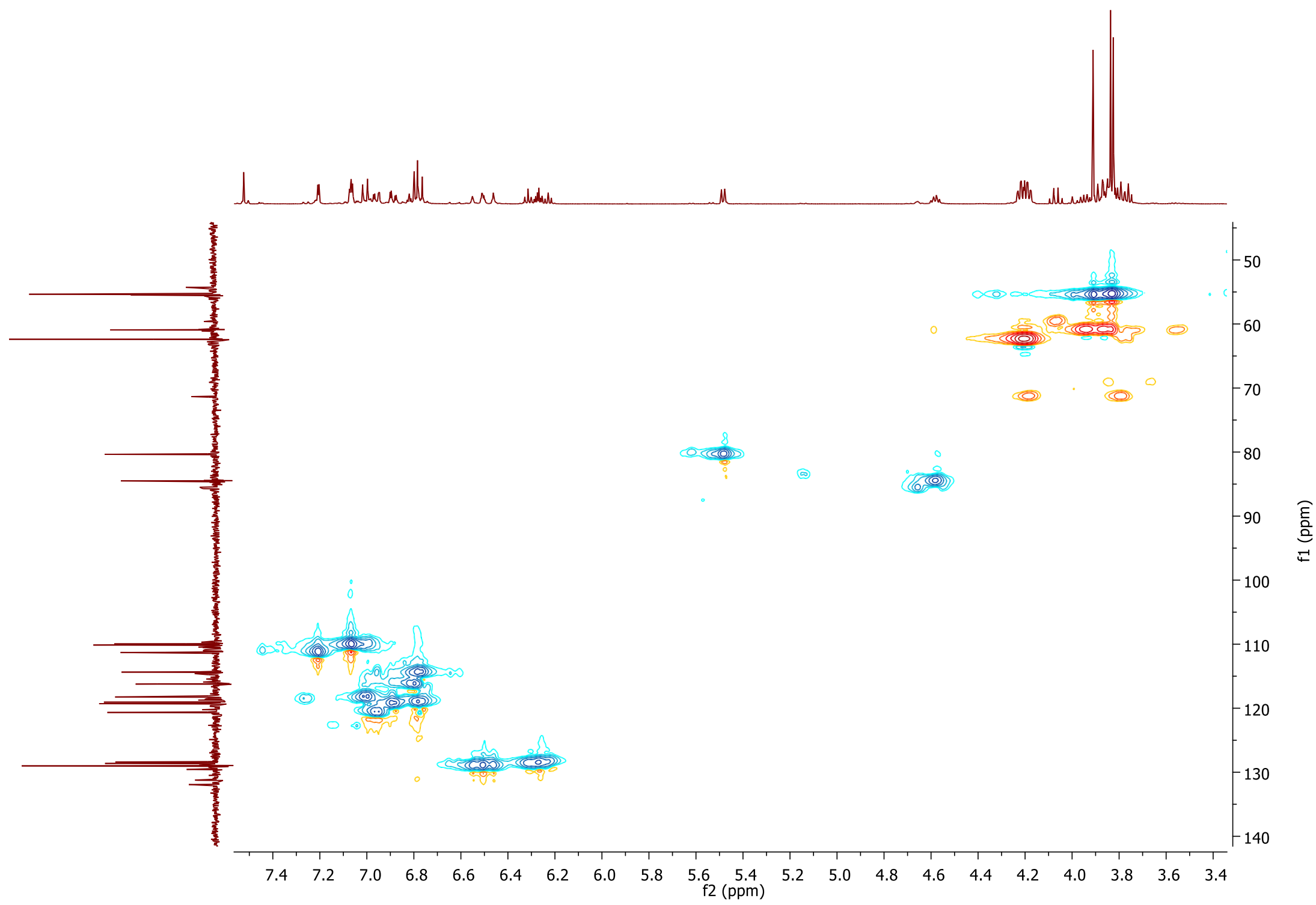

Figure S19. $^{1}H,^{13}C$ HSQC spectrum of **5** in acetone-d_6.

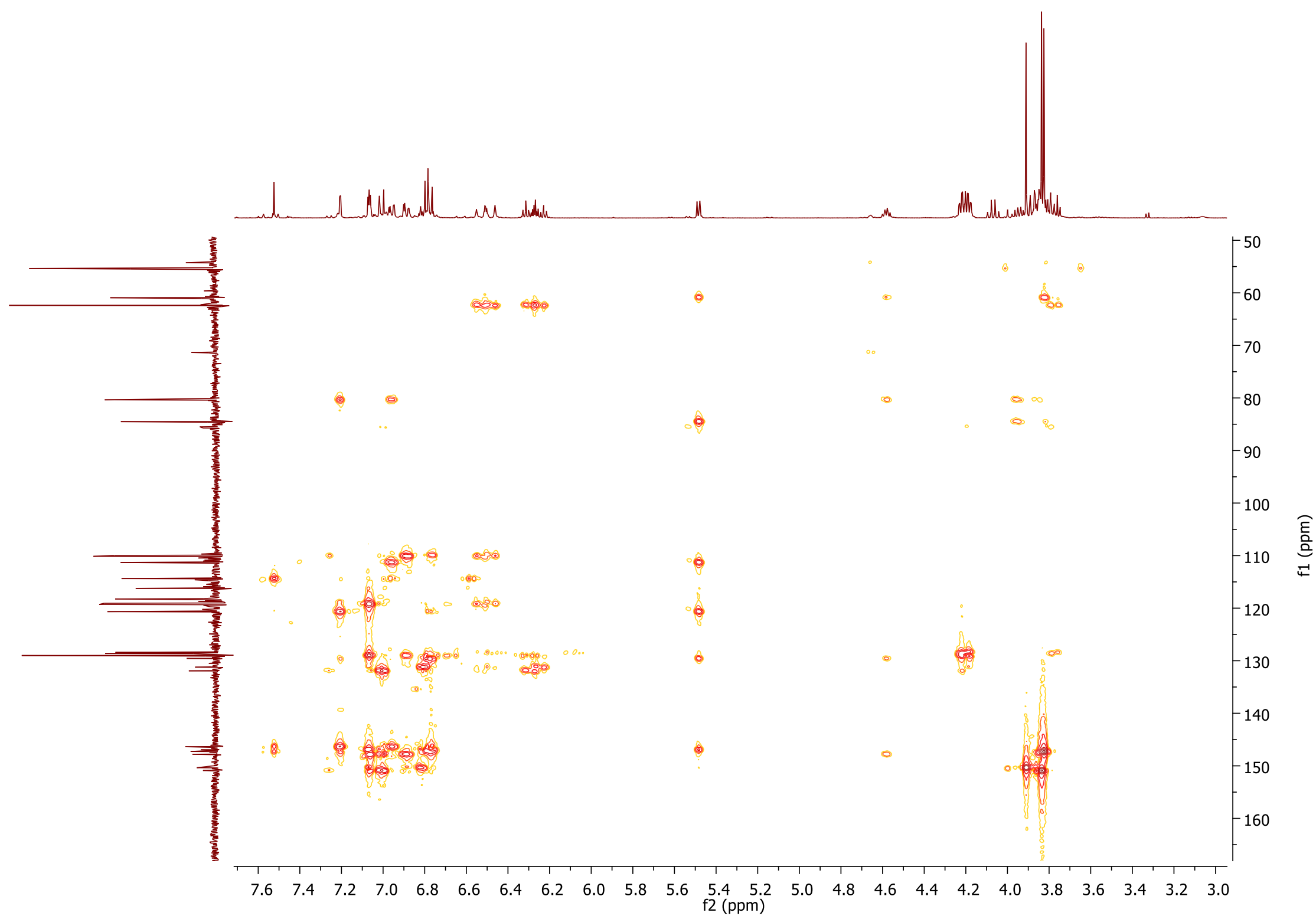

Figure S20. $^{1}H,^{13}C$ HMBC spectrum of **5** in acetone-d_6.

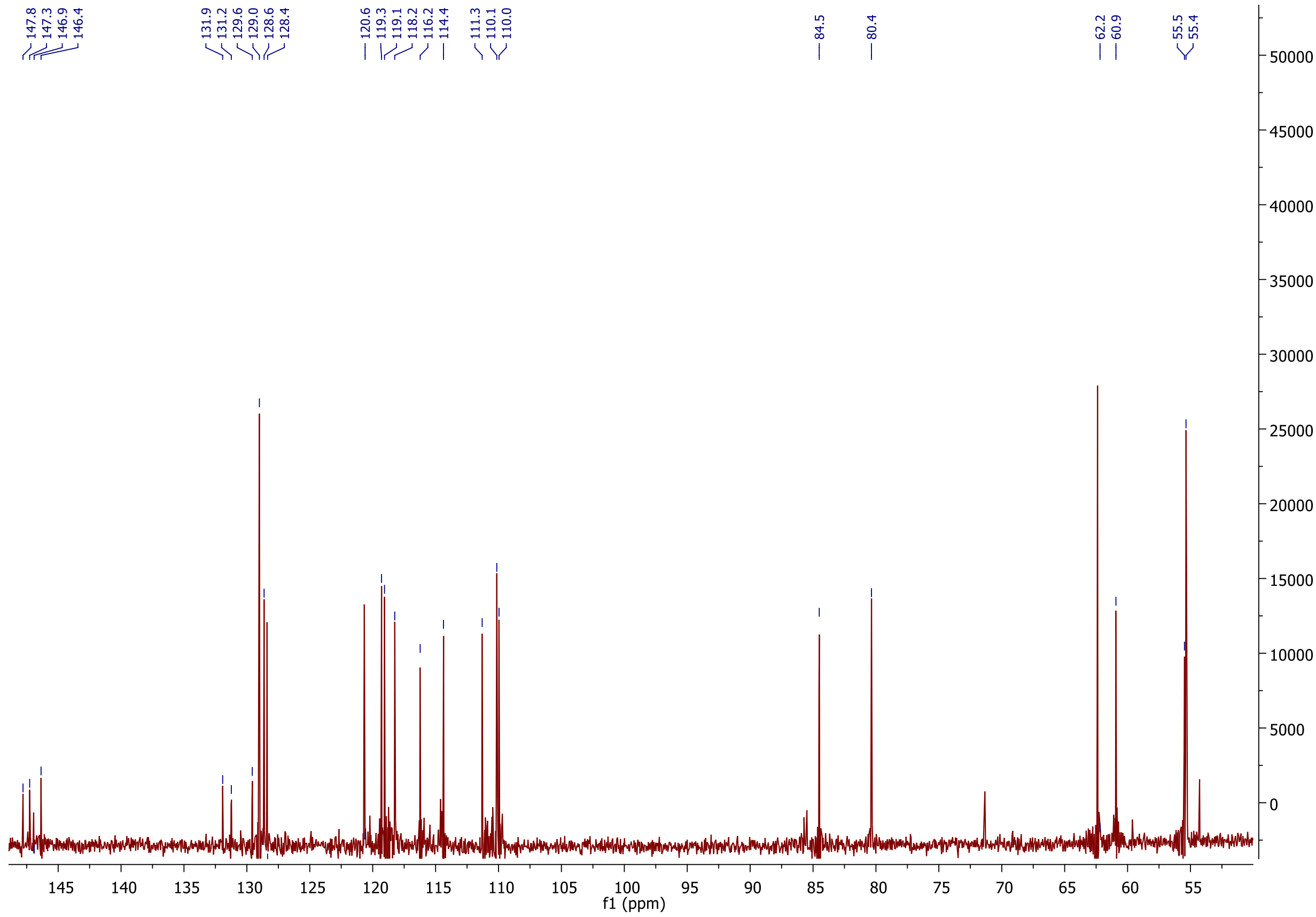

Figure S21. ^{13}C NMR spectrum of **5** in acetone-d_6.

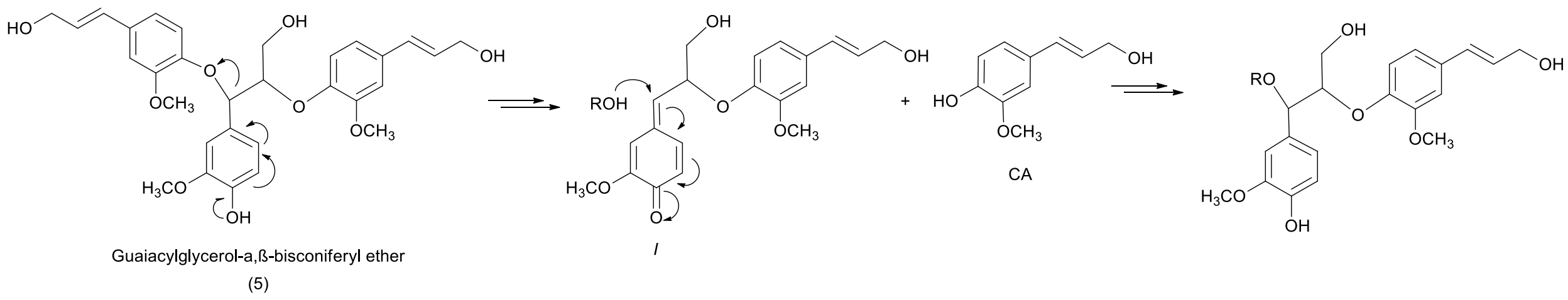

Figure S22. Proposed pathway for the degradation of **5** in the presence of methanol or water (ROH).

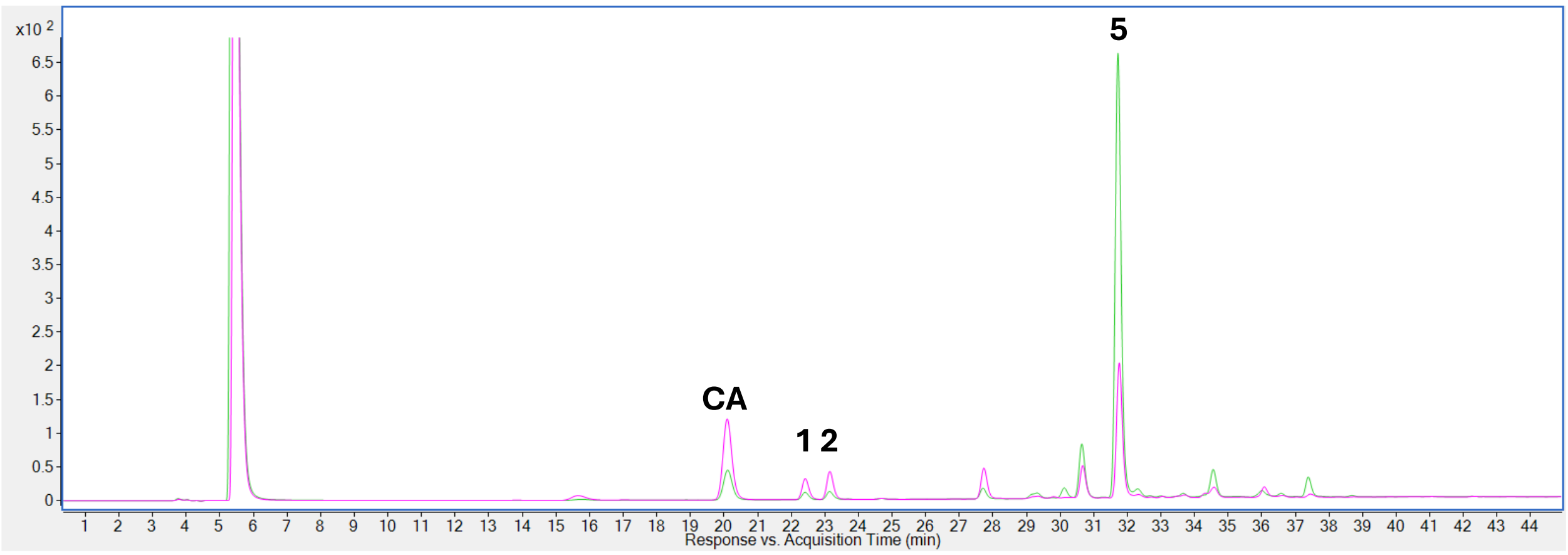

Figure S23. HPLC profiles of compound **5** in phosphate buffer at pH 6.8 at time 0 (green) and 1 h (pink). Analysis was performed under the following conditions: Eclipse Plus C18 column (150 × 4.6 mm, 5 µm). Eluent: 0.1% formic acid (solvent A)/methanol (solvent B): 30% B (0-10 min), from 30 to 80% B (10-35 min). Detection wavelength: 254 nm. CA indicates residual coniferyl alcohol. Compounds **1** and **2** are the β-O-4-coupled dimers whereas compound **5** is the trimeric derivative of CA as shown in Figure 4 and 7, respectively.

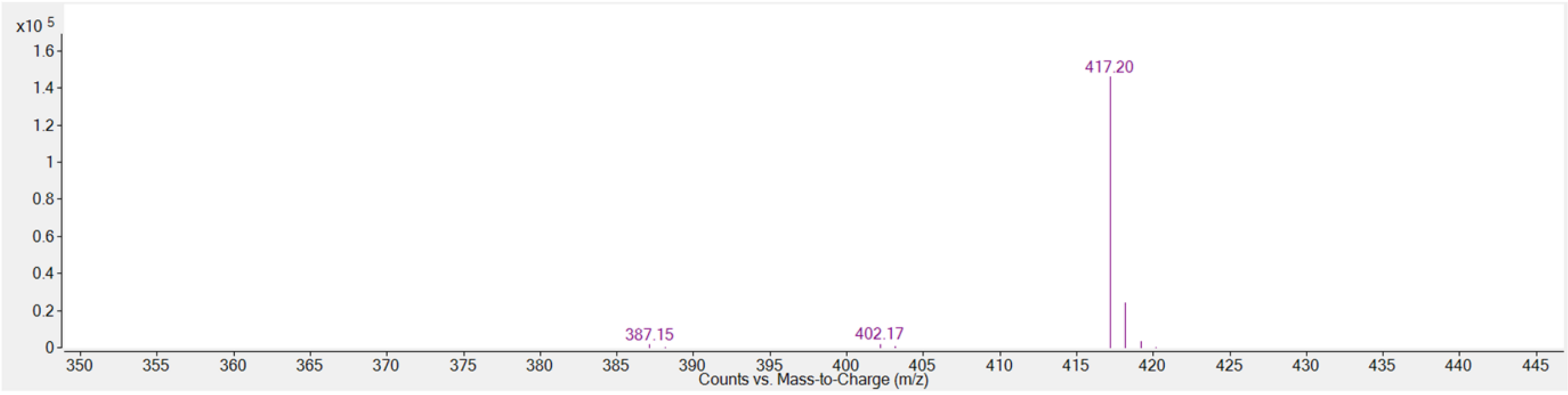

Figure S24. Mass spectrum (negative ion mode) of compound **6**.

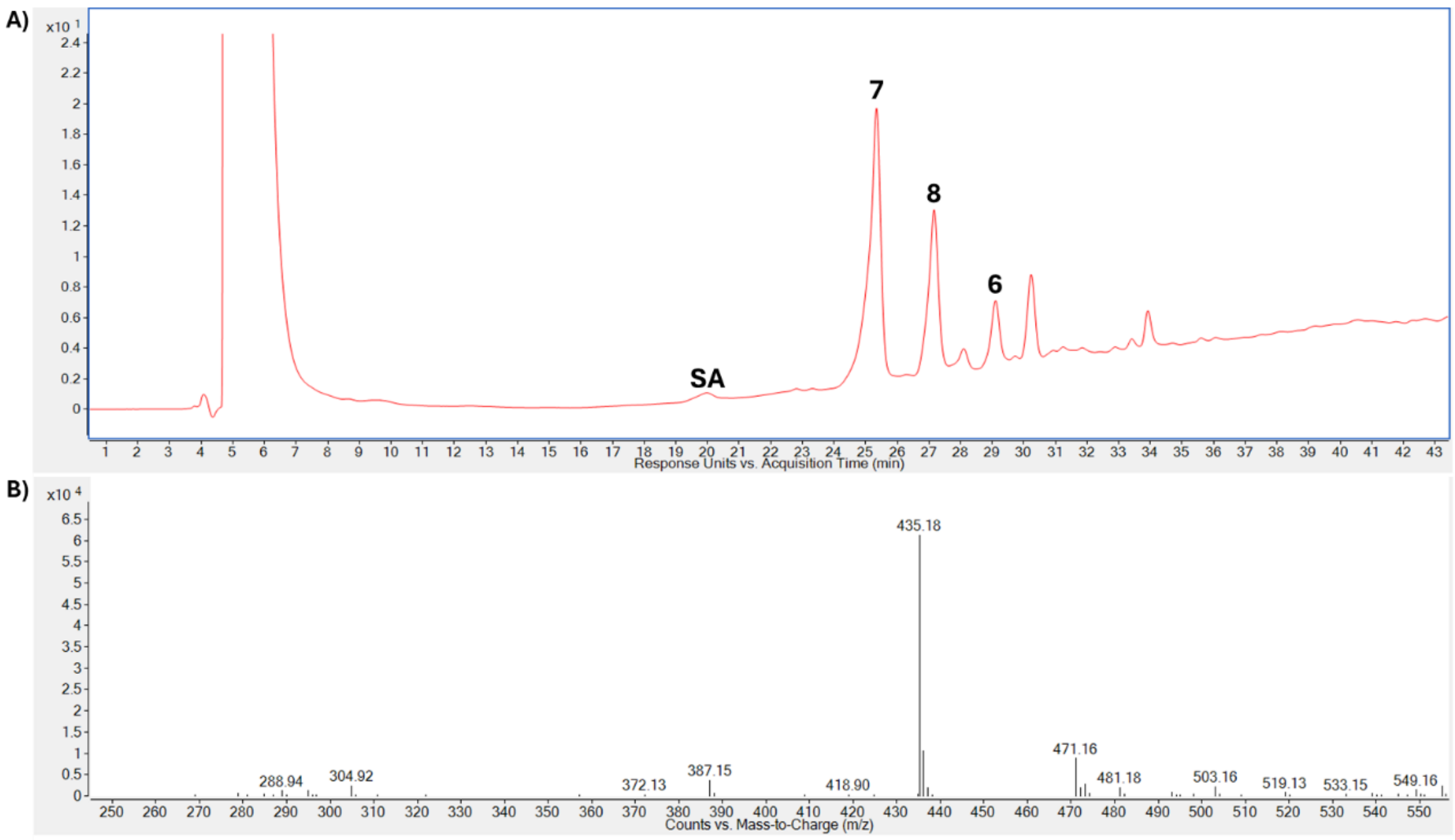

Figure S25. A) HPLC profile of the reaction mixture of sinapyl alcohol (SA) in dioxane/water mixture in the presence of $FeCl_3$ (0.8 molar eqs) after 1 h. Analysis was performed under the following conditions: Eclipse Plus C18 column (150 × 4.6 mm, 5 µm). Eluent: 0.1% formic acid (solvent A)/methanol (solvent B): 30% B (0-10 min), from 30 to 80% B (10-35 min). Detection wavelength: 254 nm. SA indicates residual sinapyl alcohol. Compound **6** is the β-β-coupled dimer whereas compounds **7** and **8** are the β-O-4-coupled dimers of SA as shown in Figure 9. B) Mass spectrum (negative ion mode) of compounds **7** and **8**.

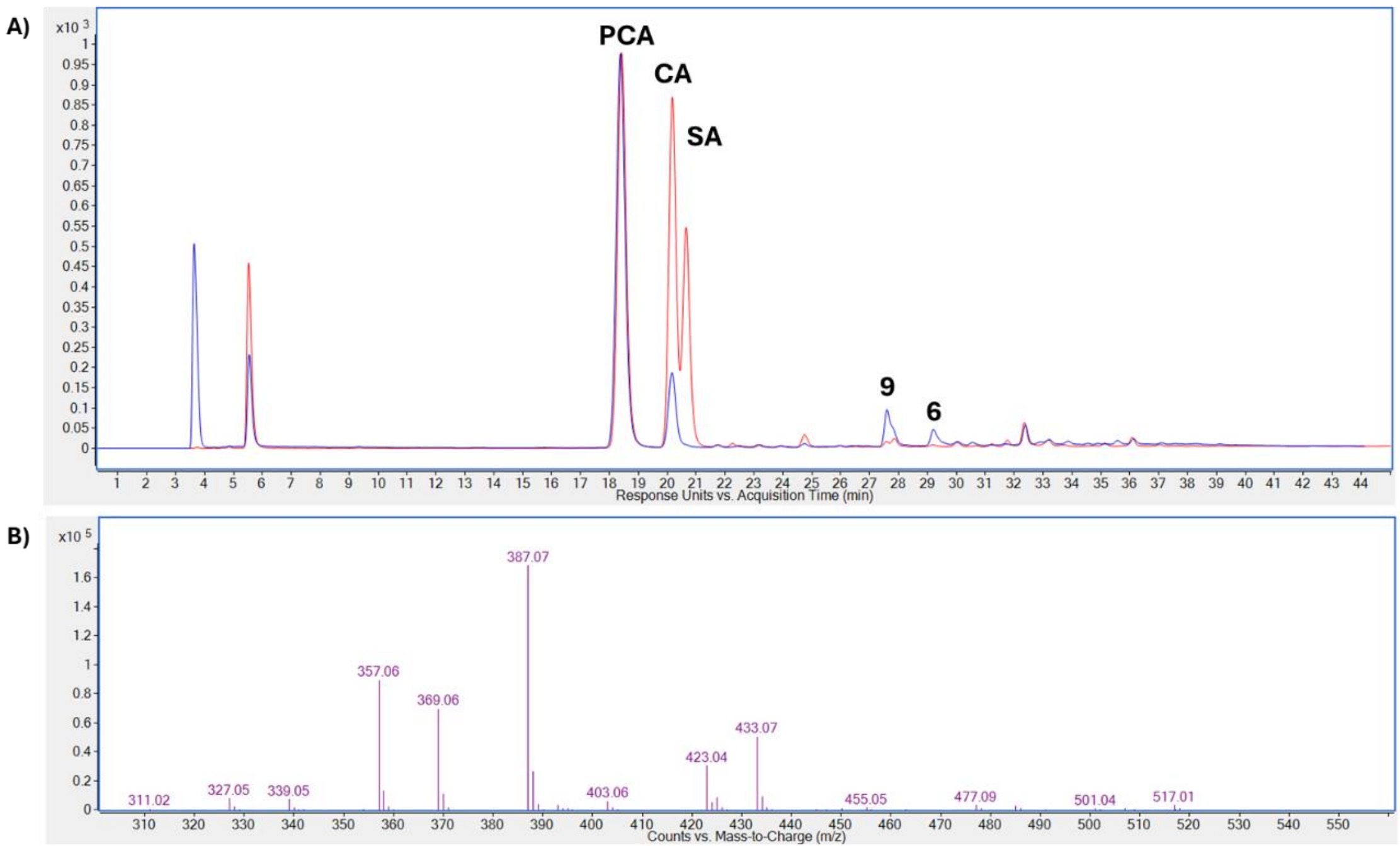

Figure S26. A) HPLC profile of the reaction mixture of coniferyl alcohol (CA), sinapyl alcohol (SA) and *p*-coumaryl alcohol (PCA) in the presence of $K_3Fe(CN)_6$ (3 mM) at time 0 (red) and after 1 h (blue). Analysis was performed under the following conditions: Eclipse Plus C18 column (150 × 4.6 mm, 5 µm). Eluent: 0.1% formic acid (solvent A)/methanol (solvent B): 30% B (0-10 min), from 30 to 80% B (10-35 min). Detection wavelength: 254 nm. PCA, CA and SA indicate residual *p*-coumaryl coniferyl and sinapyl alcohols, respectively. Compound **6** is the β-β-coupled dimer of SA whereas compound **9** is a heterodimer derived from CA and SA as shown in Figure 9 and 10, respectively. B) Mass spectrum (negative ion mode) of compound **9**.

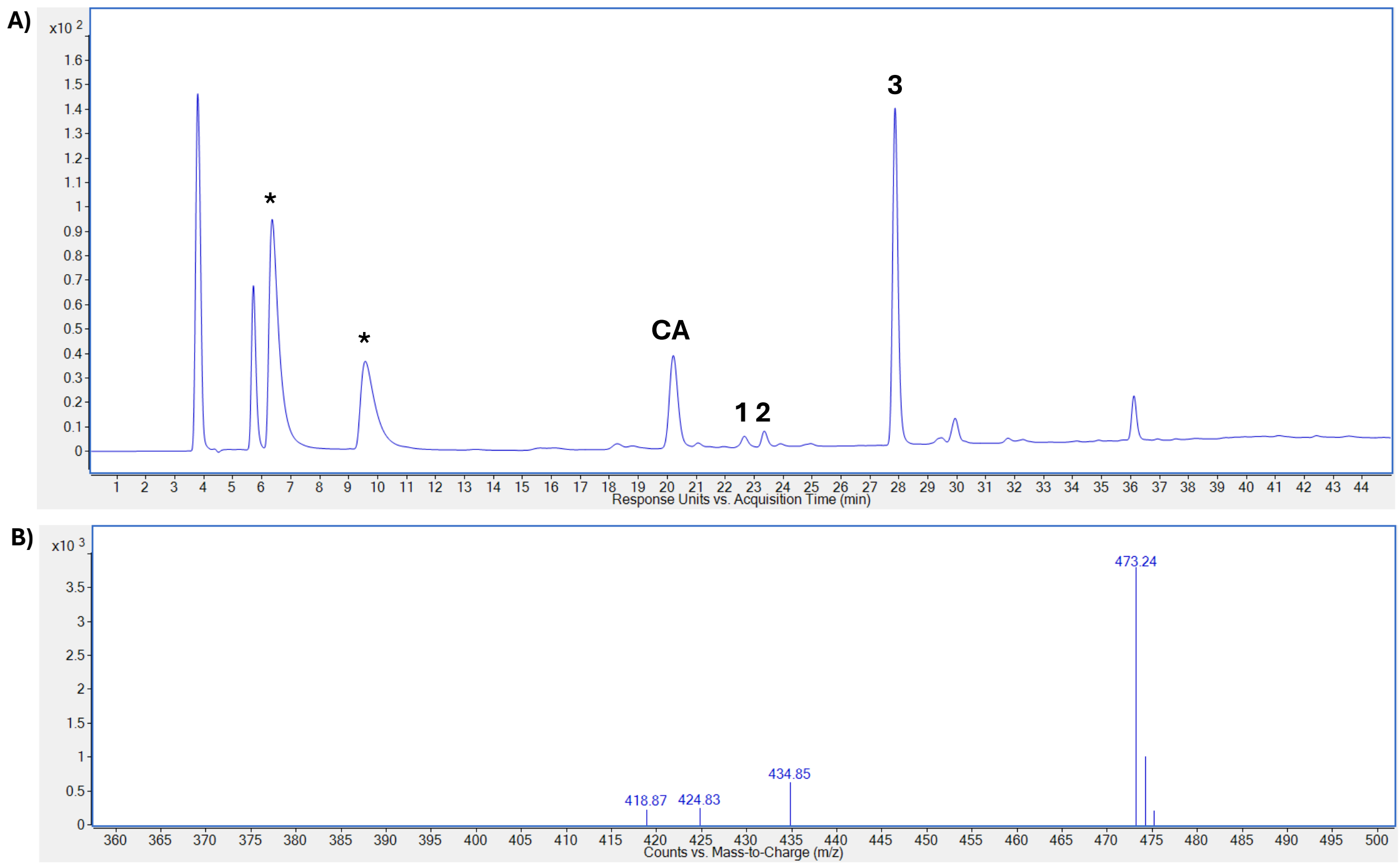

Figure S27. A) HPLC profile of the reaction mixture of coniferyl alcohol (CA) in the presence of HMDA (5 molar eqs) and $K_3Fe(CN)_6$ (1 molar eq). Analysis was performed under the following conditions: Eclipse Plus C18 column (150 × 4.6 mm, 5 µm). Eluent: 0.1% formic acid (solvent A)/methanol (solvent B): 30% B (0-10 min), from 30 to 80% B (10-35 min). Detection wavelength: 254 nm. Asterisks indicate the CA-HMDA conjugates. CA indicates residual coniferyl alcohol. Compounds **1** and **2** are the β-O-4-coupled dimers whereas compound **3** is the β-5-coupled dimer of CA as shown in Figure 4. B) Mass spectrum (negative ion mode) of the compounds eluting at 6.5 and 9.5 min.

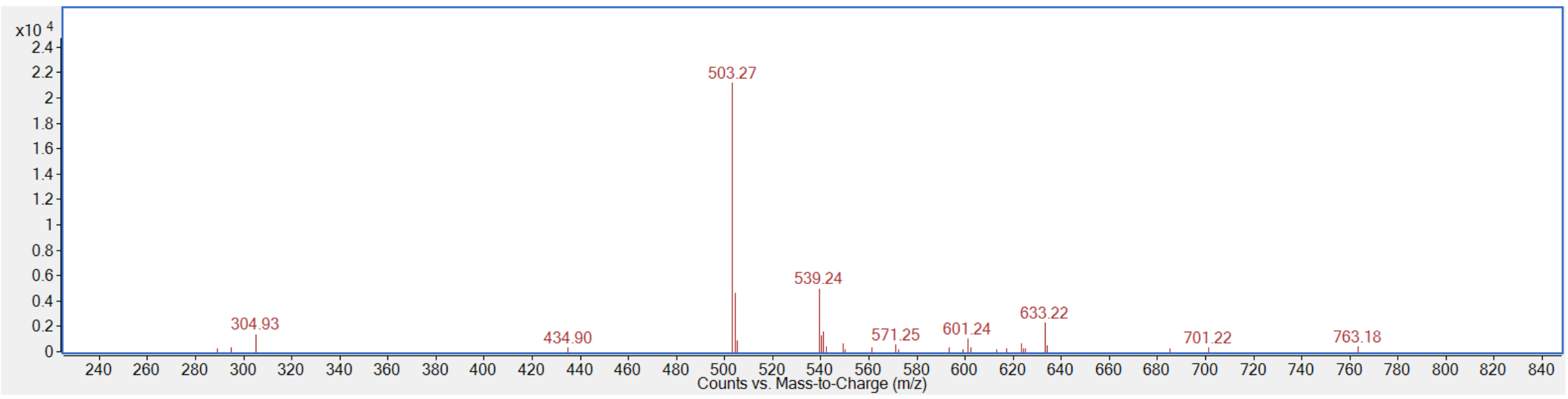

Figure S28. Mass spectrum (negative ion mode) of the compounds eluting between 8 and 17 min formed in the reaction mixture of coniferyl alcohol (CA) with lysine (10 molar eqs) and $K_3Fe(CN)_6$ (1 molar eq) (indicated with asterisks in Figure 11 in the main text).

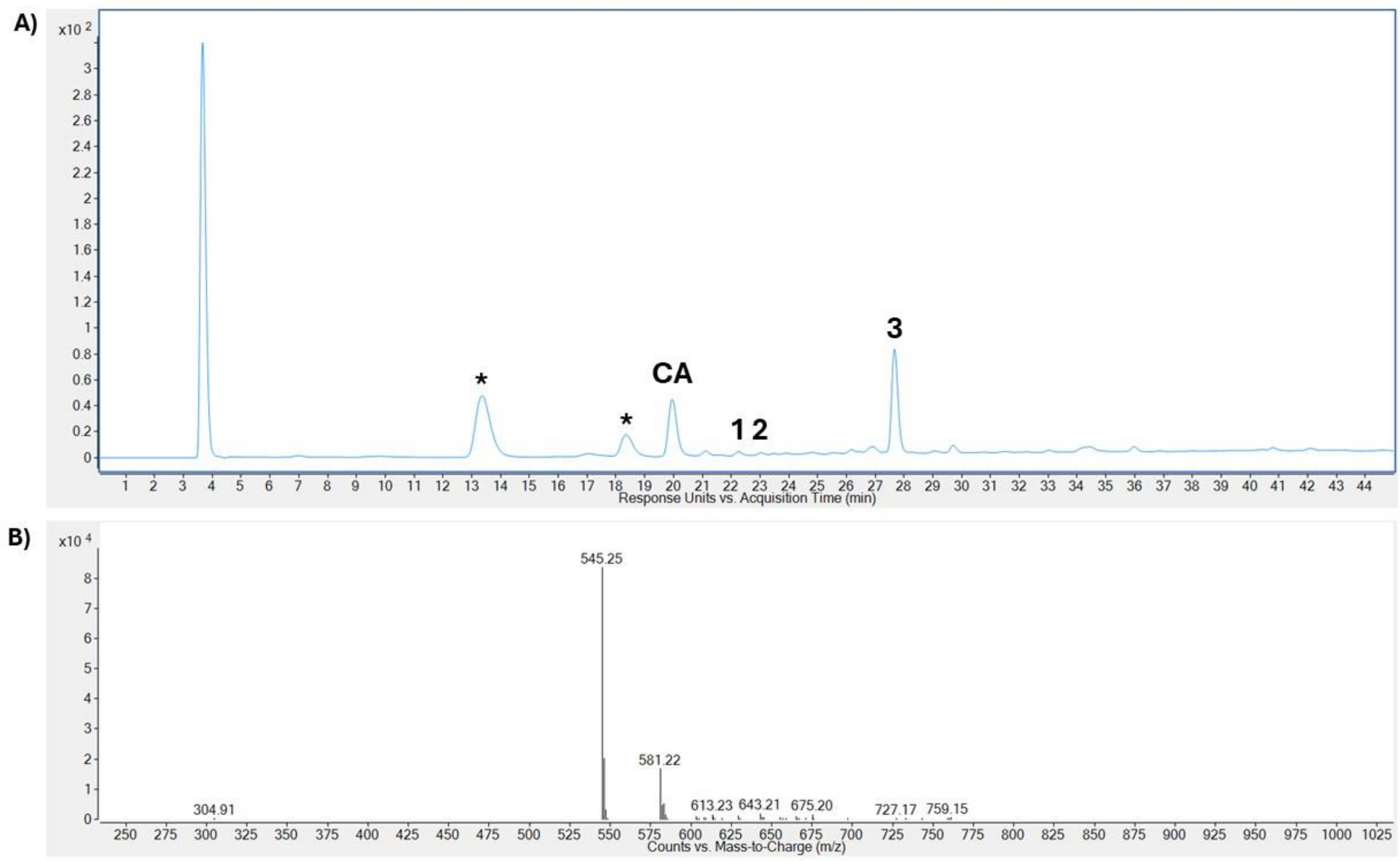

Figure S29. A) HPLC profile of the reaction mixture of coniferyl alcohol (CA) in the presence of Nα-acetyl-L-lysine methyl ester (10 molar eqs) and $K_3Fe(CN)_6$ (1 molar eq). Analysis was performed under the following conditions: Eclipse Plus C18 column (150 × 4.6 mm, 5 μm). Eluent: 0.1% formic acid (solvent A)/methanol (solvent B): 30% B (0-10 min), from 30 to 80% B (10-35 min). Detection wavelength: 254 nm. Asterisks indicate the CA-lysine derivative conjugates. CA indicates residual coniferyl alcohol. Compounds **1** and **2** are the β-O-4-coupled dimers whereas compound **3** is the β-5-coupled dimer of CA as shown in Figure 4. B) Mass spectrum (negative ion mode) of the compounds eluting at 13.5 and 18.9 min.

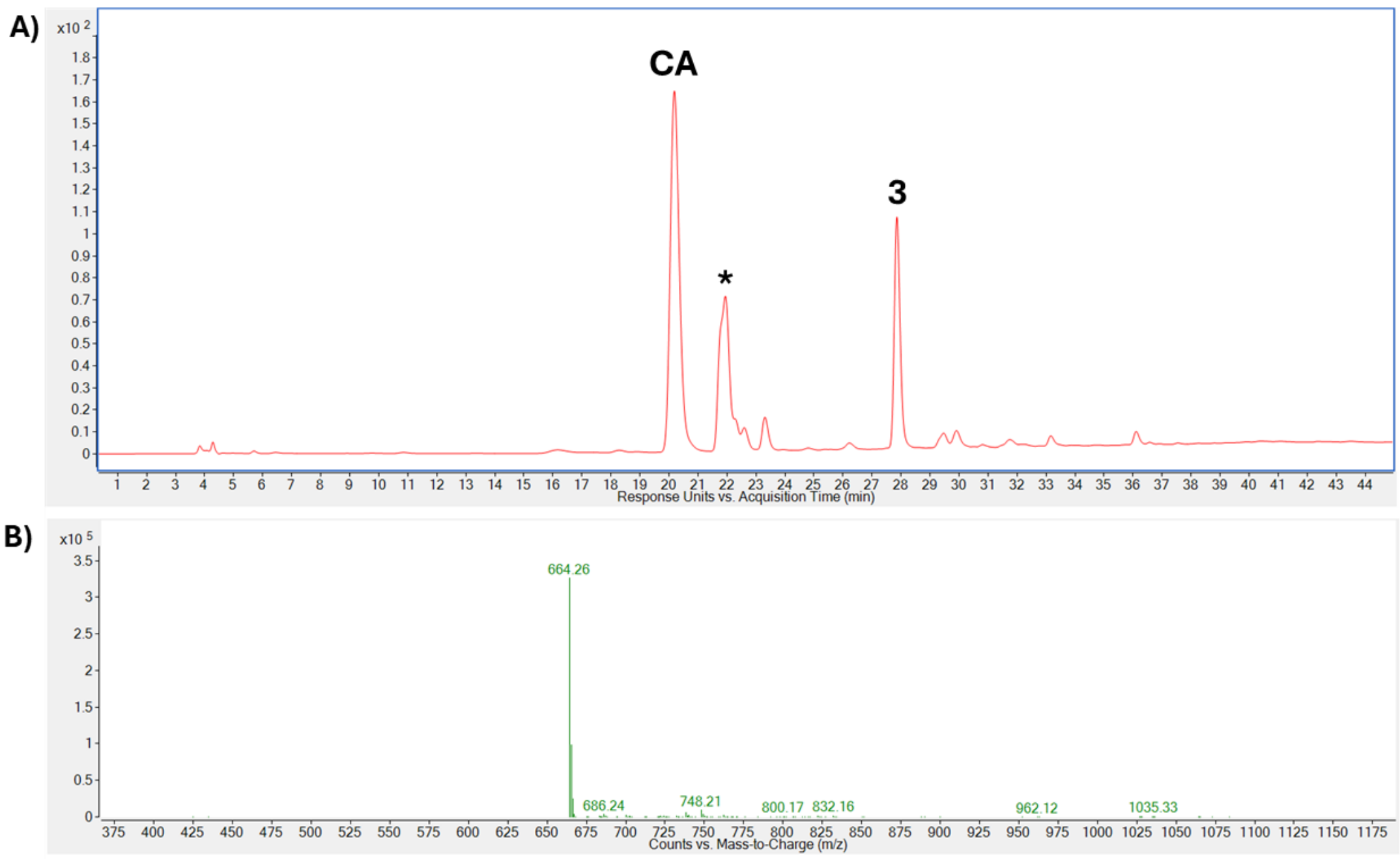

Figure S30. A) HPLC profile of the reaction mixture of coniferyl alcohol (CA) in the presence of GSH (5 molar eqs) and HRP (1 U/mL)/H_2O_2 (0.4 molar eqs). Analysis was performed under the following conditions: Eclipse Plus C18 column (150 × 4.6 mm, 5 µm). Eluent: 0.1% formic acid (solvent A)/methanol (solvent B): 30% B (0-10 min), from 30 to 80% B (10-35 min). Detection wavelength: 254 nm. CA indicates residual coniferyl alcohol. Asterisks indicate the CA-GSH conjugates. Compound **3** is the β-5-coupled dimer of CA as shown in Figure 4. B) Mass spectrum (negative ione mode) of the compounds eluting at 22 min.

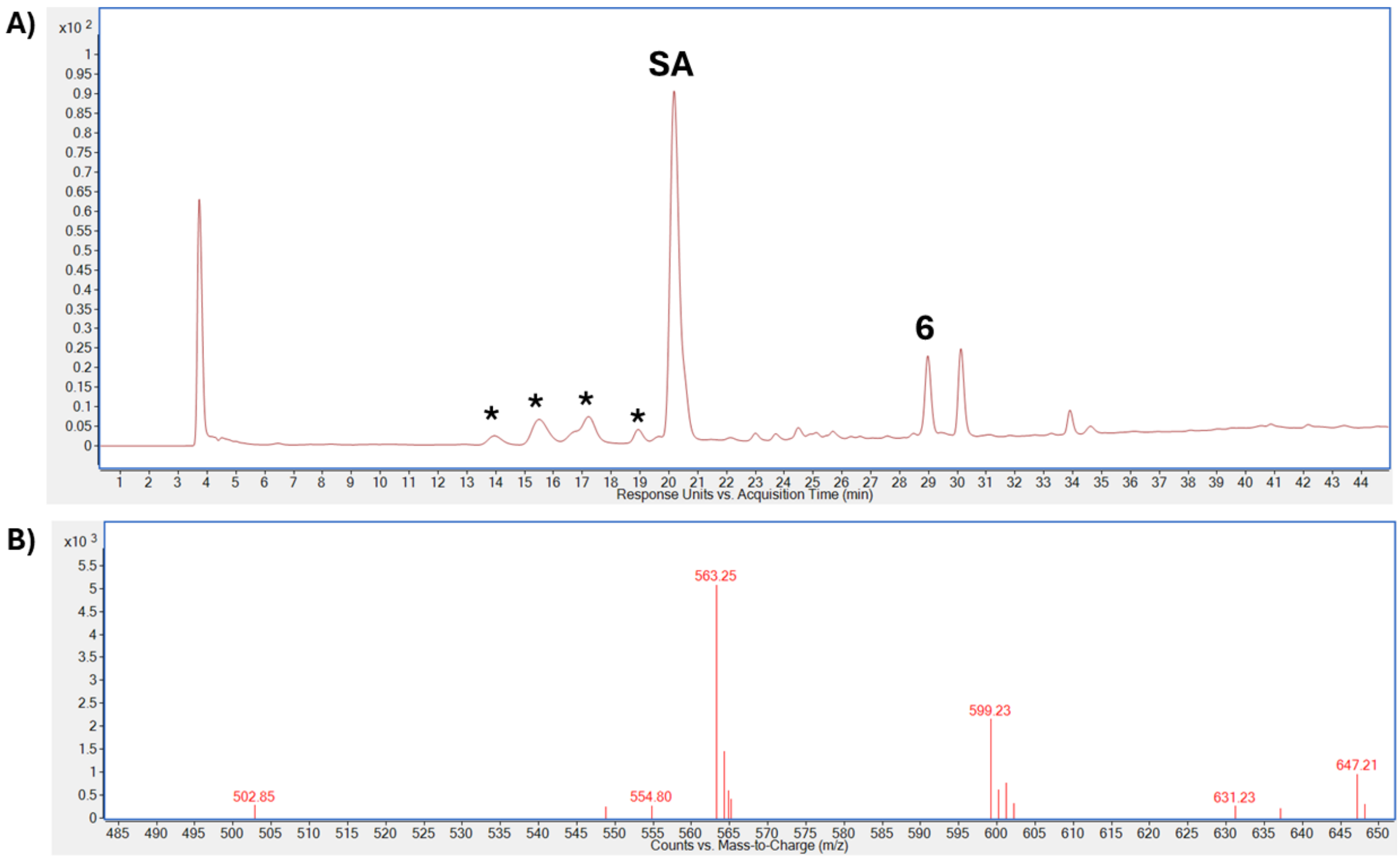

Figure S31. A) HPLC profile of the reaction mixture of sinapyl alcohol (SA) in the presence of lysine (10 molar eqs) and $K_3Fe(CN)_6$ (1 molar eq). Analysis was performed under the following conditions: Eclipse Plus C18 column (150 × 4.6 mm, 5 µm). Eluent: 0.1% formic acid (solvent A)/methanol (solvent B): 30% B (0-10 min), from 30 to 80% B (10-35 min). Detection wavelength: 254 nm. Asterisks indicate the SA-lysine conjugates. SA indicates residual sinapyl alcohol. Compound **6** is the β-β-coupled dimer of SA as shown in Figure 9. B) Mass spectrum (negative ion mode) of the compounds eluting between 14 and 19 min.

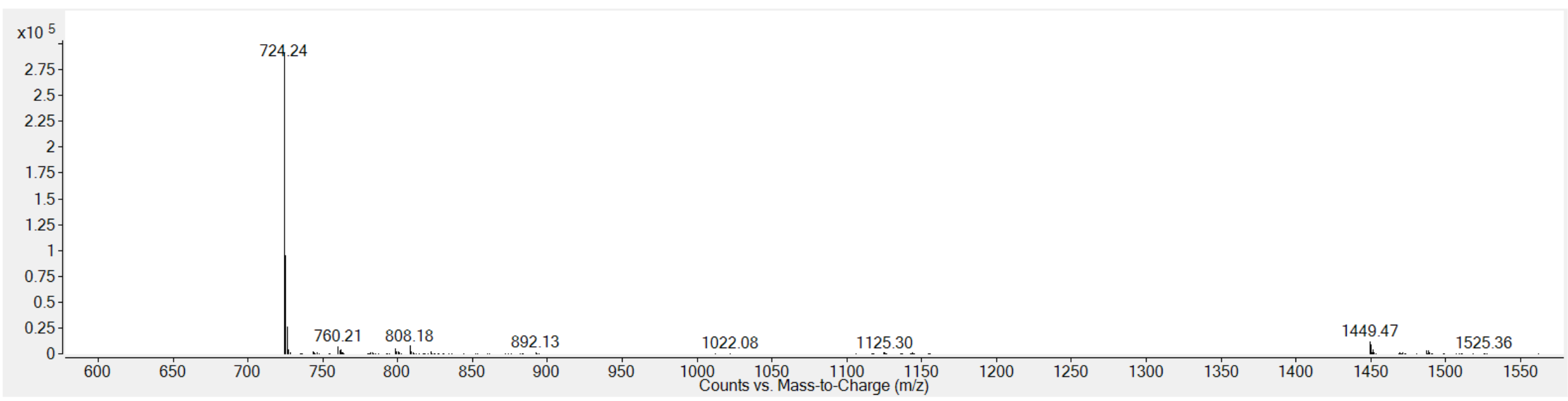

Figure S32. Mass spectrum (negative ion mode) of the compound eluting at 23 and 24 min from the reaction mixture of sinapyl alcohol (SA) in the presence of GSH (5 molar eqs) and HRP (1 U/mL)/H_2O_2 (0.2 molar eqs) (indicated with asterisks in Figure 12 in the main text).
